# Novel *β*-Cyclodextrin-Based Heptavalent Glycyrrhetinic Acid Conjugates: Synthesis, Characterization, and Anti-Influenza Activity

**DOI:** 10.3389/fchem.2022.836955

**Published:** 2022-04-12

**Authors:** Shuobin Liang, Xinyuan Ma, Man Li, Yanliang Yi, Qianqian Gao, Yongmin Zhang, Lihe Zhang, Demin Zhou, Sulong Xiao

**Affiliations:** ^1^ State Key Laboratory of Natural and Biomimetic Drugs, School of Pharmaceutical Sciences, Peking University, Beijing, China; ^2^ Sorbonne Université, Institut Parisien de Chimie Moléculaire, CNRS UMR 8232, Paris, France; ^3^ Institute of Chemical Biology, Shenzhen Bay Laboratory, Shenzhen, China; ^4^ Ningbo Institute of Marine Medicine, Peking University, Ningbo, China

**Keywords:** glycyrrhetinic acid, β-cyclodextrin, influenza virus, multivalency, hemagglutinin

## Abstract

In our continuing efforts toward the design of novel pentacyclic triterpene derivatives as potential anti-influenza virus entry inhibitors, a series of homogeneous heptavalent glycyrrhetinic acid derivatives based on *β*-cyclodextrin scaffold were designed and synthesized by click chemistry. The structure was unambiguously characterized by NMR, IR, and MALDI-TOF-MS measurements. Seven conjugates showed sufficient inhibitory activity against influenza virus infection based on the cytopathic effect reduction assay with IC_50_ values in the micromolar range. The interactions of conjugate **37**, the most potent compound (IC_50_ = 2.86 *μ*M, CC_50_ > 100 *μ*M), with the influenza virus were investigated using the hemagglutination inhibition assay. Moreover, the surface plasmon resonance assay further confirmed that compound **37** bound to the influenza HA protein specifically with a dissociation constant of 5.15 × 10^−7^ M. Our results suggest the promising role of *β*-cyclodextrin as a scaffold for preparing a variety of multivalent compounds as influenza entry inhibitors.

## 1 Introduction

Influenza A virus is a highly contagious respiratory pathogen that can cause seasonal epidemics and irregular pandemics because of its rapid transmission and frequent genetic alteration (Neumann et al., 2009; Lagacé-Wiens et al., 2010). The structure of the virus consists of a lipid envelope that is generated from the host cell to which two dominant membrane proteins, hemagglutinin (HA) (80%, ∼300 copies of trimer) and neuraminidase (NA) (17%, ∼50 copies of tetramer), are anchored (Leser and Lamb, 2005). Based on the antigenicity of HA and NA, they can be classified into different subtypes. To date, 18 major antigenic variants of HA and 11 antigenic variants of NA have been recognized, which are found in numerous combinations. In addition, newly mutated forms of the influenza virus appear every year. The currently available options to combat influenza A viral infections include prophylactic, yearly reformulated vaccines and two classes of anti-influenza drugs (the M2 ion-channel inhibitors and the NA inhibitors) (Das, 2012). Since the currently available vaccines offer a very limited breadth of protection (van Dongen et al., 2019), the antiviral drugs are still the first-line protection to treat acute influenza infection. Unfortunately, all circulating influenza A viral strains appear to be resistant to M2 ion-channel inhibitors. Recently, the continually emerging resistant virus against NA inhibitors has also been reported (Hurt et al., 2006; van der Vries et al., 2010; Hurt, 2014). For these reasons, novel anti-influenza viral agents with new targets and mechanisms of action are urgently required to overcome the rapid emergence of drug resistance (Heo, 2018; van Dongen et al., 2019).

In the first step of infection, the virion adheres to the host cell surface through the binding of HA to sialic acid-terminated carbohydrates present on the cell membranes (Mammen et al., 1998; Skehel and Wiley, 2000). Therefore, HA plays a pivotal role in virus entry, making it a potential target for antiviral intervention. HA is a homotrimeric integral membrane glycoprotein, which has ca. 300–400 copies on the viral surface (Mammen et al., 1998). The monovalent interaction between HA and sialic acid is mediated essentially by hydrogen bonds, and their dissociation constant shows a relative weak binding (*K*_D_ ∼ 2 mM) (Sauter et al., 1989). Therefore, a multitude of HA is involved in order to increase the overall strength of the interaction between the virus and the host cell. The binding between multiple pairs of HA trimer and sialic acid ligand increases substantially as characterized by a multivalent affinity constant of estimated 10^13^ M^−1^ (Mammen et al., 1998). This process provides important clues for the design of efficient multivalent ligands to mimic the binding of HA to sialic acid to block the viral protein–host receptor interactions (Lu and Pieters, 2019). Over the past few years, many efforts have been made for the design and synthesis of carbohydrate-based multivalent HA binders, including polymers, dendrimers, nanoparticles, and so on, serving as potent influenza A virus inhibitors (Chaudhary et al., 2019; Lu and Pieters, 2019).

**FIGURE 1:**
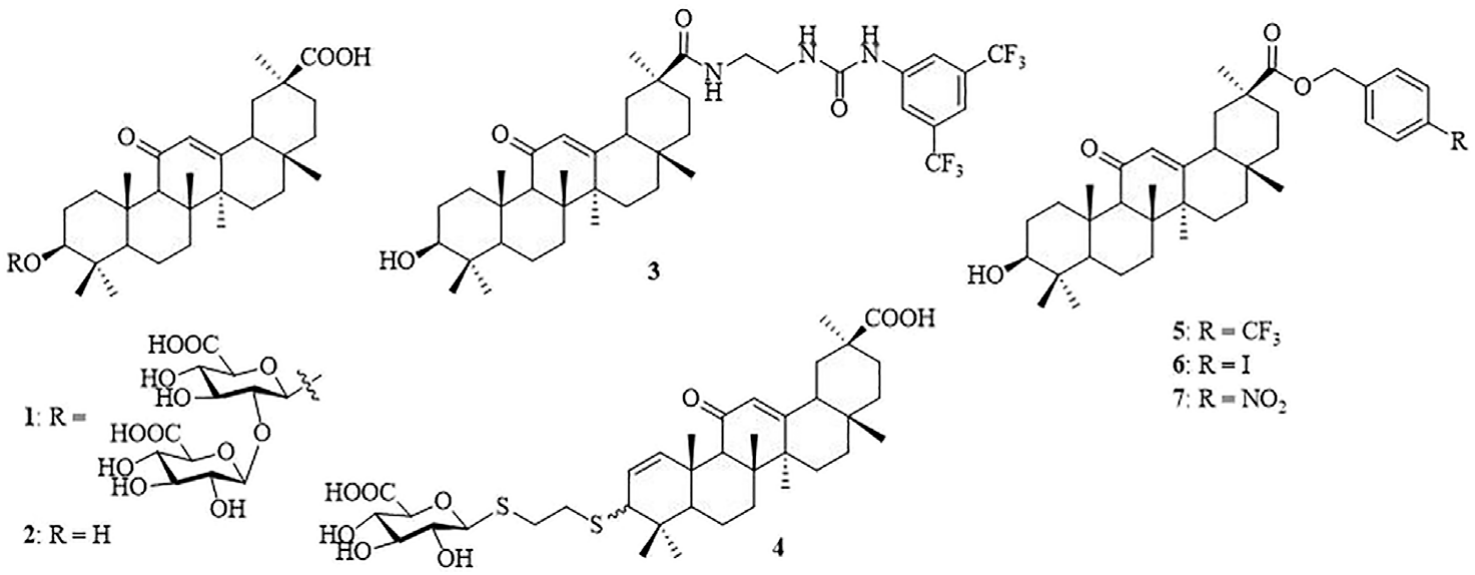
Chemical structures of glycyrrhizic acid (**1**), GA (**2**), and its derivatives (**3**–**7**).

*β*-Cyclodextrin (*β*-CD) is a torus-shaped cyclic oligosaccharide consisting of seven (*α*-1,4)-linked D-glucopyranose units, and it is well-known for its ability to form inclusion complexes with various organic and inorganic compounds (Crini, 2014). It is widely used in pharmaceutical formulations to enhance the solubility of poorly soluble drugs, increase drug permeability through biological membranes, and improve the bioavailability of drugs (Jansook et al., 2018). The unique steric accessibility and acidity of the three types of hydroxyls in *β*-CD have been taken into account to conceive efficient position-selective and face-selective chemical functionalization methodologies (Khan et al., 1998). It has been shown to serve as a multivalent scaffold in the design of glycoconjugates (Martinez et al., 2013), glycoclusters (Gomez-Garcia et al., 2005; Gomez-Garcia et al., 2012), glycodendrimers (Ortega-Caballero et al., 2001; Vargas-Berenguel et al., 2002), and star polymers (Zhang et al., 2012; Gonzalez-Gaitano et al., 2017). In addition, target-specific multivalent drug delivery systems have shown significant improvement of anti-tumor drug delivery and hence antitumor efficacy and prevention of clinical multi-drug resistance (Kim et al., 2017; Zhou et al., 2019). In spite of the progress, the design of multivalent ligands with optimized biological properties still remains a challenge considering the complexity of the multivalent ligand–receptor interactions (Lepage et al., 2015). Glycyrrhizic acid (also known as glycyrrhizin, **1**) (Figure 1), an oleanane-type pentacyclic triterpene saponin, is the primary bioactive constituent of the roots and rhizomes of *Glycyrrhiza glabra* (licorice) which is employed as an herbal medicine in both Western and Eastern countries (Fiore et al., 2005; Asl and Hosseinzadeh, 2008). Compound **1** and its aglycone, glycyrrhetinic acid (GA, **2**), have been reported to possess antitumor, anti-inflammatory, and other pharmacological activities (Yang et al., 2015; Hussain et al., 2018). In a notable example, Lallemand *et al.* have reported that compound **3**, with a 2-(3-(3,5-bis(trifluoromethyl)phenyl)ureido)ethyl group at C-30 of **2**, shows strong antitumor activity with IC_50_ in signal-digit micromolarity in a panel of eight cancer cell lines (Lallemand et al., 2011). Recently, attention to GAs has greatly increased because of their broad spectrum of antiviral activities, such as the anti-hepatitis B virus (HBV) (Wang et al., 2012), anti-Epstein–Barr virus (EBV) (Lin et al., 2008), anti-rotavirus (Hardy et al., 2012), anti-influenza virus (Stanetty et al., 2012), and anti-herpes simplex virus (HSV) (Zigolo et al., 2018). 3-Thioglucuronide derivative **4** and several C-30 ester derivatives, such as 4-(trifluoromethyl) benzyl ester (**5**), 4-iodobenzyl ester (**6**), and 4-nitrobenzyl ester (**7**), show promising antiviral activity (Stanetty et al., 2012; Wang et al., 2012). In our previous study, a series of monovalent *β*-CD-GA conjugates have been synthesized and several conjugates showed weak antiviral activity at high concentration (∼50 *μ*M) (Liang et al., 2019). As a natural step in our efforts toward the design of new pentacyclic triterpene conjugates with antiviral activity (Yu et al., 2014; Xiao et al., 2016; Si et al., 2018), we described herein the design and synthesis of a series of heptavalent GA functionalized *β*-CD conjugates and evaluated their *in vitro* anti-influenza activity.

## 2 Materials and Methods

### 2.1 Materials

GA was kindly supplied by Nanjing Zelang Medical Technology Co., Ltd. (China). *β*-CD, *O*-(benzotriazol-1-yl)-*N,N,N',N'*-tetramethyluroniumtetrafluoroborate (TBTU), *N,N*-Diisopropylethylamine (DIPEA), iodine, triphenylphosphine, sodium azide, 4-dimethylaminopyridine (DMAP), and cupric sulfate were purchased from Shanghai Aladdin Bio-Chem Co., Ltd. (China). Sodium ascorbate, sodium methoxide, 5-hexyn-1-amine, and 4-ethynylaniline were supplied by Shanghai Energy Chemicals (China). Silica gel 60 (200–300 mesh) was provided by Qingdao Haiyang Chemical Co., Ltd. (China). Sodium carbonate, *N,N*-dimethylformamide, tetrahydrofuran, methanol, pyridine, acetic anhydride, and all other chemicals and reagents used were of analytical grade and purchased from Sinopharm Chemical Reagent Co., Ltd. (China) and used throughout this research without further purification.

Thin-layer chromatography (TLC) was conducted on a pre-coated silica gel 60 F_254_ plate (E. Merck, Darmstadt, Germany) and eluted with a mixture of methanol/dichloromethane (15:1–10:1) for per-*O*-acetyl-*β*-CD-GA conjugates or methanol for the deacetylated *β*-CD-GA conjugates. For spot detection, the plates were immersed in a yellow solution of Ce(NH_4_)_2_(NO_3_)_6_ (0.5 g)/(NH_4_)_6_Mo_7_O_24_^**.**^4H_2_O (24.0 g)/6% H_2_SO_4_ (500 ml). The plate was heated by using a hot gun to visualize the spots. FT-IR spectra were obtained using a Nicolet Nexus 470 FTIR spectrometer (Thermo Electron Scientific Instruments LLC, United States). MALDI-TOF mass spectra were obtained on an AB Sciex TOF/TOF™ 72115 spectrometer using methanol or chloroform as solvents and *α*-cyano-4-hydroxy-cinnamic acid (HCCA) as matrix. NMR spectra were recorded on a Bruker DRX 400 or DRX 600 spectrometer at ambient temperature.

Madin Darby canine kidney (MDCK) cells were donated by Crown Bioscience Inc. (United States). Dulbecco’s Modified Eagle Medium (DMEM) was purchased from Gibco BRL, Inc. (United States). The CellTiter-Glo^®^ reagent was purchased from Promega Corp., Inc. (United States). Recombinant influenza HA was purchased from Sino Biological Inc. (China). Deionized double-distilled water was used throughout the biological study. The surface plasmon resonance (SPR) assay was analyzed using the Biacore 8k system (GE Healthcare, Uppsala, Sweden).

### 2.2 Synthesis of Heptavalent β-CD-GA Conjugates

Heptakis (2,3-di-*O*-acetyl-6-deoxy-6-azide-)-*β*-CD **11** was prepared in a three-step procedure as previously reported (Gadelle and Defaye, 1991). The GA intermediate **12** was synthesized based on the method of Schwarz *et al.* with minor modifications (Schwarz et al., 2014). The terminal alkynyl substituted amines **13–16** were prepared as previously described (Murelli et al., 2009; Zhang et al., 2010; Tran et al., 2013; Sanhueza et al., 2017). Alkynyl-functionalized GA intermediates **17**–**20** and **30–32** were synthesized as previously described (Liang et al., 2019). Heptavalent GA functionalized *β*-CD conjugates were synthesized through a two-step process involving the copper-catalyzed alkyne–azide cycloaddition reaction (CuAAC) under microwaves (step 1) and de-*O*-acetylation reaction under Zemblén conditions (step 2).

#### 2.2.1 General Procedure A for the Click Reaction (Step 1)

To a solution of heptakis (2,3-di-*O*-acetyl-6-deoxy-6-azide-)-*β*-CD **11** (189.8 mg, 0.10 mmol) and alkynyl-functionalized GA derivatives (0.84 mmol) in 50% of THF and H_2_O (5 ml) was added CuSO_4_ (15.7 mg, 0.10 mmol) and sodium ascorbate (30.7 mg, 0.15 mmol). The resulting solution was heated in a microwave reactor at 100°C until the azide was completely consumed (typically about 1 h) as determined by TLC. Then, the reaction mixture was extracted with CH_2_Cl_2_ (10 ml × 3). The CH_2_Cl_2_ fraction was dried over Na_2_SO_4_, filtered, evaporated, and purified by silica gel column chromatography using CH_2_Cl_2_/CH_3_OH (15:1–10:1 v/v) as the eluent to give the acetylated intermediates as white foam.

#### 2.2.2 General Procedure B for the Deacetylation Reaction (Step 2)

The multivalent GA functionalized per-*O*-acetylated *β*-CD conjugates were dissolved in dry methanol (5 ml per 100 mg of compound), and a solution of sodium methoxide (30% in methanol, 0.1 eq per mol of acetate) was added. The solution was stirred (180 rpm) at room temperature for 4–6 h in a N_2_ atmosphere. After completion (TLC), the reaction mixture was neutralized with Amberlite IR-120 (H^+^) ion exchange resin, then filtered and concentrated. The crude product was purified by RP column chromatography using CH_3_OH as the eluent to give the final products in 73–95% yields.

The ^1^H and ^13^C NMR and ESI-HRMS or MALDI-TOF MS data of the synthesized new compounds **21–28** and **33–38** are available in Supplementary Data S6–S13.

### 2.3 Fourier Transform Infrared Spectroscopy Analysis

FT-IR spectra of the heptavalent *β*-CD-GA conjugates and their parent compounds **11** and **17** were obtained using a Nicolet Nexus 470 FTIR spectrometer (Thermo Electron Scientific Instruments LLC, United States) over a scanning range of 500–4,000 cm^−1^. In addition, 1.0 mg of different samples was mixed with KBr pellets.

### 2.4 Nuclear Magnetic Resonance Spectroscopy Study

^1^H and ^13^C NMR spectra of GA derivatives **17**–**20** and **29**–**32** were acquired on a Bruker DRX 400 MHz spectrometer. ^1^H NMR, ^13^C NMR, ^1^H–^1^H COSY NMR, and ^1^H-^13^C HSQC NMR spectra of *β*-CD-GA conjugates **21**–**28** and **33**–**38** were acquired on a Bruker DRX 600 MHz spectrometer. Sample solutions were prepared with CDCl_3_ or CDCl_3_/CD_3_OD (2:1) as solvent. CDCl_3_ or CD_3_OD were also used as reference: proton (*δ* 7.26 ppm) and carbon (*δ* 77.00 ppm) for CDCl_3_, proton (*δ* 3.31 ppm) and carbon (*δ* 49.00 ppm) for CD_3_OD. The detection temperature was set at 25°C.

### 2.5 Cell Culture and Viruses

The MDCK cells were donated by Crown Bioscience Inc. (United States) and grown in DMEM supplemented with 10% (v/v) fetal bovine serum (FBS) (PAA Laboratories, Linz Austria) at 37°C under 5% CO_2_. The A/WSN/33 (H1N1) influenza virus used in this study was artificially generated from the 12-plasmid influenza virus rescue system.

### 2.6 Cytotoxicity Assay

Cell viability assays were performed using the CellTiter-Glo^®^ reagent following the manufacturer’s instructions. Briefly, the cells (1×10^4^ per well) were seeded into 96-well tissue culture plates and incubated for 16 h at 37°C with 5% CO_2_ to allow the cells to adhere to the surface of the wells. The culture medium was then replaced with a fresh medium containing compound or paclitaxel (PTX) at 10 and 25 *μ*M in triplicate, and the negative control wells contained the equivalent volume of the medium with 1% DMSO, after which they were incubated for 48 h at 37°C with 5% CO_2_. The CellTiter-Glo^®^ reagent was added, and the plates were read using a Tecan Infinite M2000 PRO™ plate reader.

### 2.7 Cytopathic Effect Reduction Assay

The MDCK cells (1 × 10^4^ per well) were seeded into 96-well plates, incubated at 37°C with 5% CO_2_ for 24 h, and infected with the influenza virus (MOI = 0.1). The cells were suspended in DMEM supplemented with 1% FBS, containing different concentrations of the test compound or Oseltamivir (OSV) and 2 *μ*g/ml TPCK-treated trypsin, and a final DMSO concentration of 1% was added to each well. After 48 h of incubation at 37°C with 5% CO_2_, the CellTiter-Glo reagent (Promega Corp., Madison, WI, United States) was added, and the cells were assessed with the CellTiter-Glo^®^ assay according to the manufacturer’s instructions.

### 2.8 Hemagglutination Inhibition Assay

HI assay was performed as described previously (Yu et al., 2014). Briefly, compound from a 2-fold serial dilution in saline was mixed with an equal volume of the influenza virus (2 HA units) in the V-bottomed 96-well microplates. Subsequently, 50 μL of freshly prepared chicken red blood cells (cRBCs) (1% v/v in saline) was added to each well. The mixture was incubated at room temperature for 30 min before observing cRBC aggregation on the plate.

### 2.9 Surface Plasmon Resonance Assay

Interactions between influenza HA and the compounds were analyzed using the Biacore 8k system (GE Healthcare, Uppsala, Sweden) at 25°C. Recombinant influenza HA (Sino Biological Inc., Beijing, China) was immobilized on a sensor chip (CM5) using EDC and NHS for activation. The final HA immobilized levels were typically ∼9,000 RU. Subsequently, compounds were injected as analytes at various concentrations, and PBS-T (10 mM phosphate buffer with 5% DMSO, 0.05% Tween, pH 7.4) was used as the running buffer. For binding studies, the analytes were applied at corresponding concentrations in the running buffer at a flow rate of 30 μL/min with a contact time of 60 s and a dissociation time of 180 s. Chip platforms were washed with the running buffer and 50% DMSO. Data were analyzed with the Biacore insight evaluation software (version 1.05) by curve fitting using a binding model of 1:1.

## 3 Results and Discussion

### 3.1 Synthesis of Heptavalent β-CD-GA Conjugates

**SCHEME 1:**
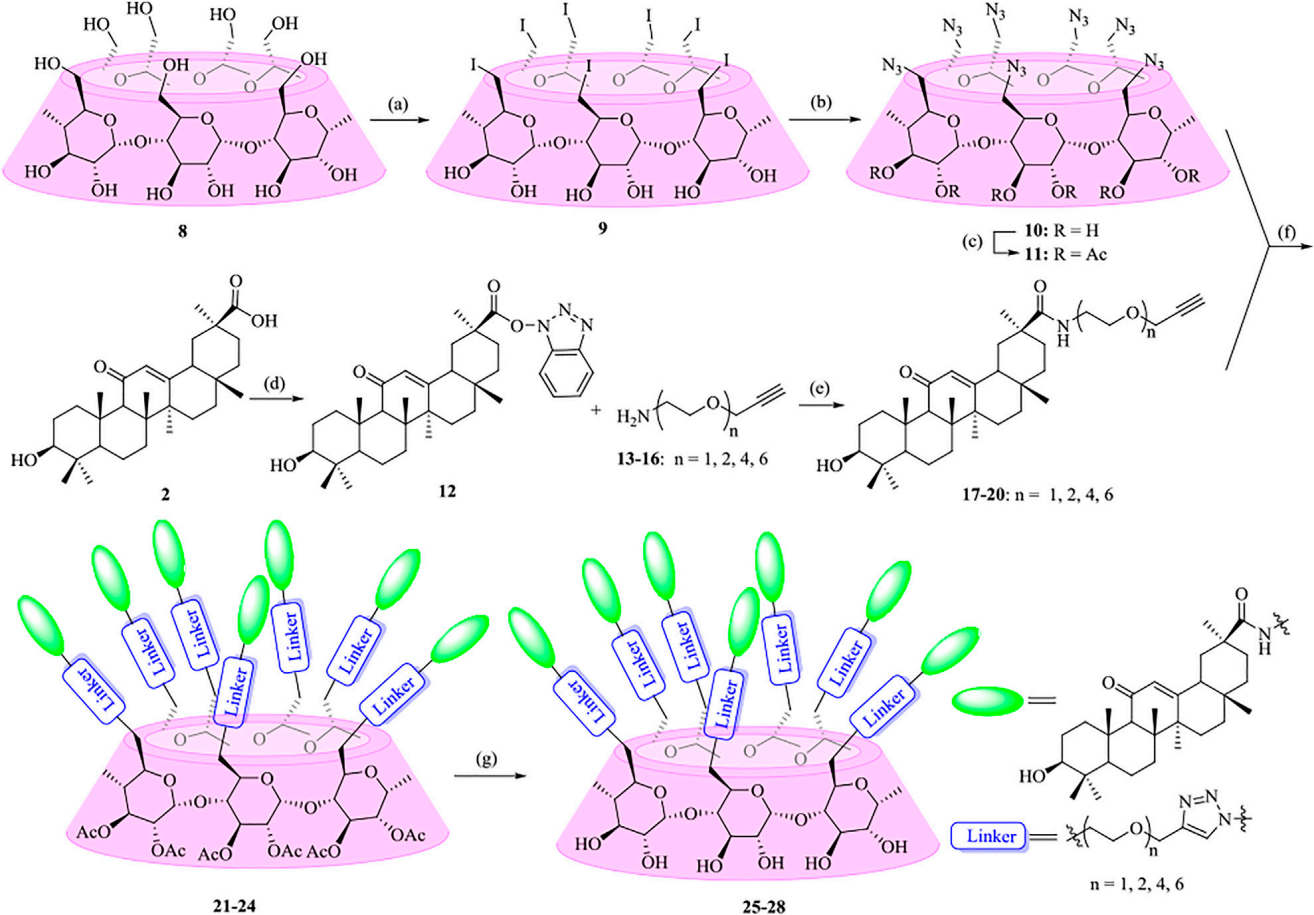
Synthesis of heptavalent GA-functionalized *β*-CD conjugates (**21**–**28**). Reagents and conditions: (a) I_2_, Ph_3_P, DMF, 80^o^C, 44%; (b) NaN_3_, DMF, 60^o^C, 90%; (c) Py, Ac_2_O, DMAP, 92%; (d) TBTU, DIPEA, THF, 90%; (e) Na_2_CO_3_, DMF, 60^o^C, 46–82%; (f) Sodium ascorbate, CuSO_4_, THF-H_2_O (1:1, v/v), 54–88%; (g) MeOH, MeONa, 73–86%.

As shown in Scheme 1, the synthetic strategy was based on coupling of alkynyl-functionalized gas with heptaazide-substituted *β*-CD via click chemistry. At first, oligo (ethylene glycols) (OEGs) were chosen as the linkers to enhance and optimize the properties of the conjugates. Various heterobifunctional OEGs with amine and alkynyl terminals **13–16** were synthesized as described previously (Murelli et al., 2009; Zhang et al., 2010; Tran et al., 2013; Sanhueza et al., 2017). The coupling of OEGs **13–16** with compound **12**, which was obtained from commercially available GA **2** by the activation of the carboxylic acid at C-30, was performed to provide the alkynyl-functionalized GAs **17**–**20**. Then **17**–**20** underwent a “click chemistry” reaction with heptakis (2,3-di-*O*-acetyl-6-deoxy-6-azide)-*β*-CD **11**, which was prepared from *β*-CD **8** in three steps using the conventional method as previously described (Gadelle and Defaye, 1991; Ashton et al., 1996; Karginov et al., 2006) in the presence of copper sulfate and sodium ascorbate as the reducing agent to give **21–24** with yields ranging from 54% to 88%. At last, the acetyl groups of multivalent conjugates **21**–**24** were removed under Zemblén conditions, followed by neutralization with H^+^ resin to afford **25–28** in 73–86% yields.

**SCHEME 2:**
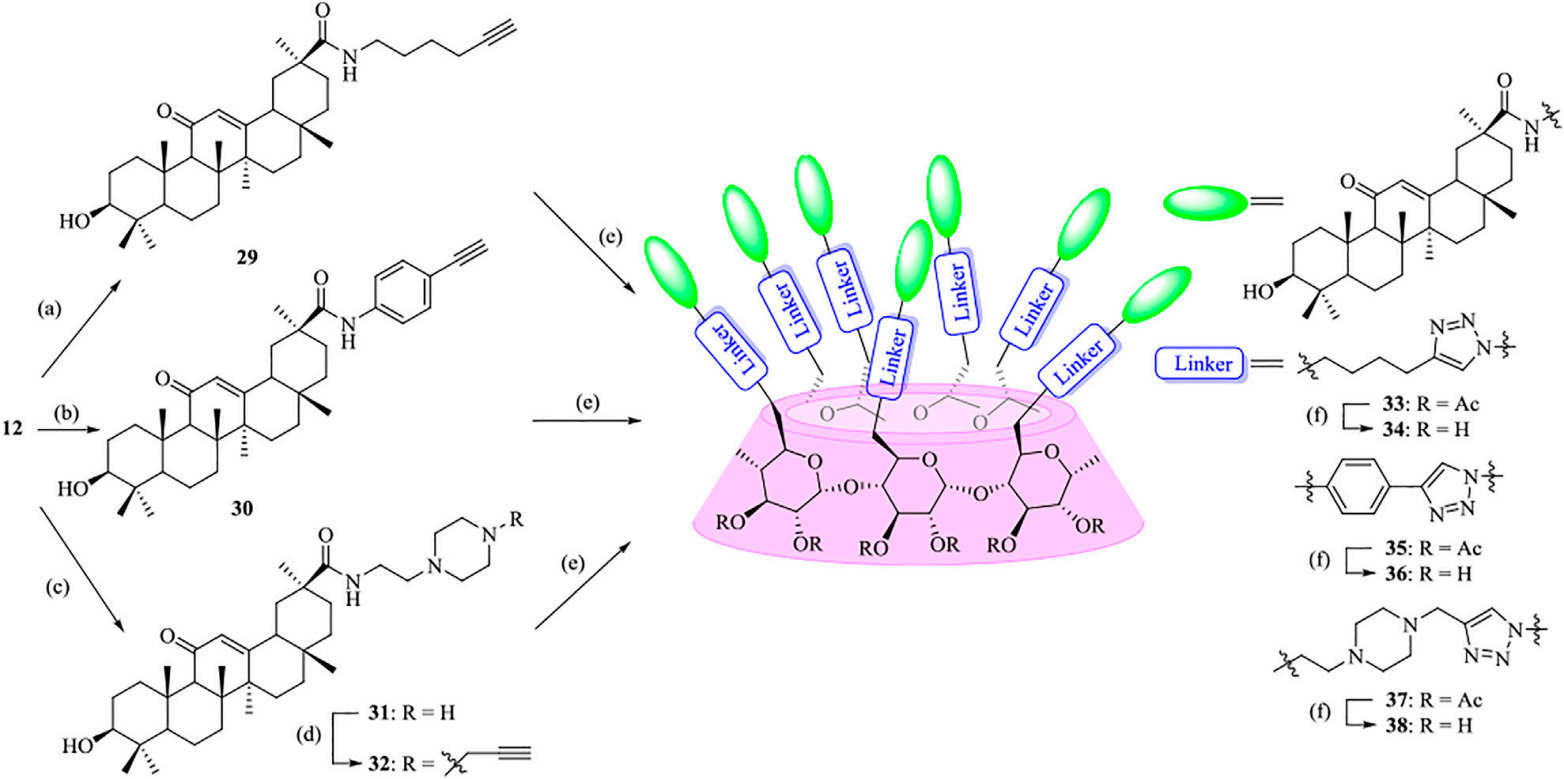
Synthesis of heptavalent GA-functionalized *β*-CD conjugates (**33**–**38**). Reagents and conditions: (a) 5-hexyn-1-amine, Na_2_CO_3_, DMF, 60^o^C, 90%; (b) 4-ethynylaniline, Na_2_CO_3_, DMF, 60^o^C, 30%; (c) 2-(piperazin-1-yl)ethan-1-amine, Na_2_CO_3_, DMF, 60^o^C, 45%; (d) propargyl bromide, Na_2_CO_3_, DMF, 60^o^C, 76%; (e) **11**, sodium ascorbate, CuSO_4_, THF-H_2_O (1:1, v/v), 42–59%; (f) MeOH, MeONa, 90–95%.

To highlight the effect of the linkers on the anti-influenza virus activity, a parallel experiment was carried out to conjugate GA with *β*-CD moiety through an alkyl chain or a more rigidness chain, such as a benzene ring or piperazine ring, rather than an OEG linker (Scheme 2). In a similar pathway, the coupling of compounds **29**, **30,** and **32** with heptaazide *β*-CD intermediate **11** was performed *via* click reaction, followed by de-*O*-acetylation under Zemplén conditions to give the corresponding conjugates **34**, **36,** and **38**, respectively, as the final products.

### 3.2 Structure Characterization of Heptavalent β-CD-GA Conjugates

The structures and symmetrical substitution of the heptavalent *β*-CD-GA conjugates **21–28** and their precursors **33–38** were characterized by IR, NMR, and MALDI-TOFMS spectroscopy.

#### 3.2.1 Fourier Transform Infrared Spectroscopy Analysis

**FIGURE 2:**
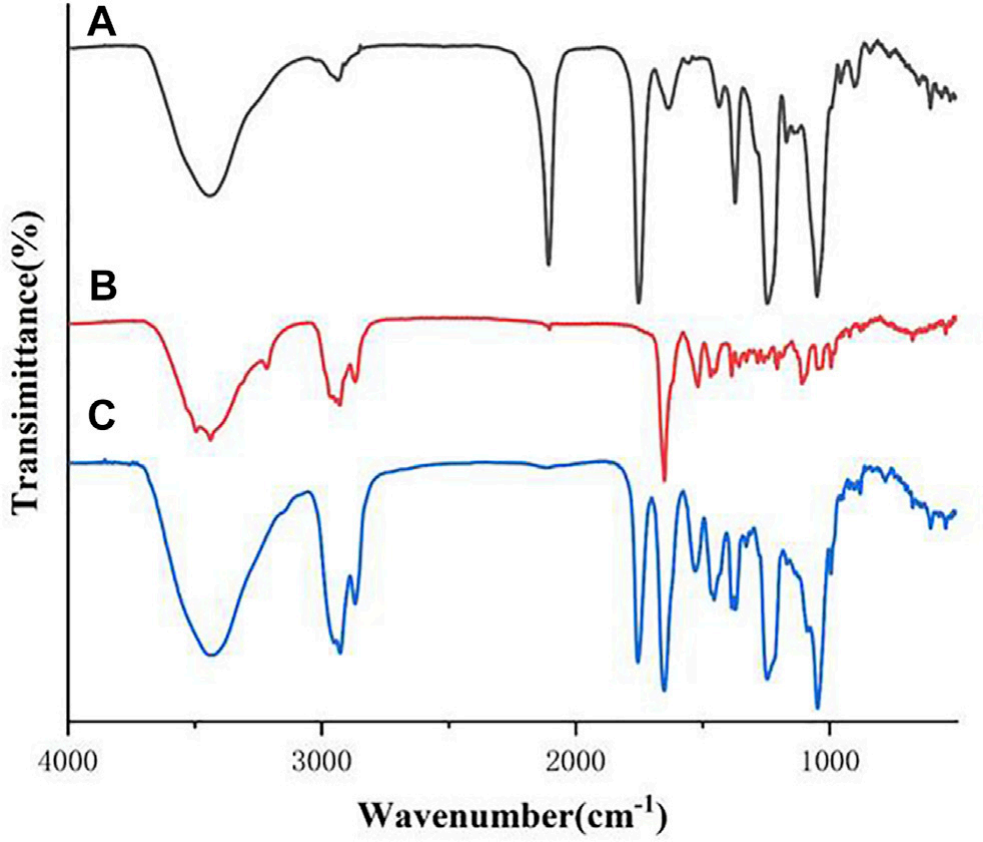
FTIR spectra of compounds **11** [**(A)**, black], **17** [**(B)**, red], and their conjugate **21** [**(C)**, blue]. Spectra were acquired between 500 and 4,000 cm^−1^.

The FTIR spectra of the all synthesized heptavalent *β*-CD-GA conjugates were recorded over the range of 400–4,000 cm^−1^ in a Nicolet Nexus 470 FT-IR thermo-scientific spectro-photometer. The spectrum of *β*-CD intermediate **11** contained the characteristic absorption band for the spectrum with OH stretching at 3,386 cm^−1^, CH stretching around 2,924 cm^−1^, and CO stretching around 1,030 cm^−1^ (Supplementary Figure S1). For GA (**2**), several vibration characteristics of the triterpenic moiety were present. The characteristic band attributed to the stretching vibration of the OH group at C3 was found at 3,443 cm^−1^. The bands at 2,930 and 2,870 cm^−1^ were assigned to the stretching vibration of the aliphatic CH (Supplementary Figure S2). The spectra of these heptavalent *β*-CD-GA conjugates were very similar. In a typical example, Figure 2 shows the IR spectra of the two intermediates **11** and **17** and their conjugate **21**. It was noteworthy that the strong absorption band located at 2,107 cm^−1^ shown in Figure 2A and the weak absorption band located at 2,120 cm^−1^ shown in Figure 2B, which should be assigned to the stretching vibration of azide and terminal alkyne, respectively, were diminished in the spectrum of **21** (Figure 2C). In addition, a few representative absorption bands ascribable to 1,2,3-triazole units were shown around 1,529, 1,455, and 1,047 cm^−1^ (Rao and Venkataraghavan, 1964).

#### 3.2.2 NMR and MALDI-TOF MS Analysis

**FIGURE 3:**
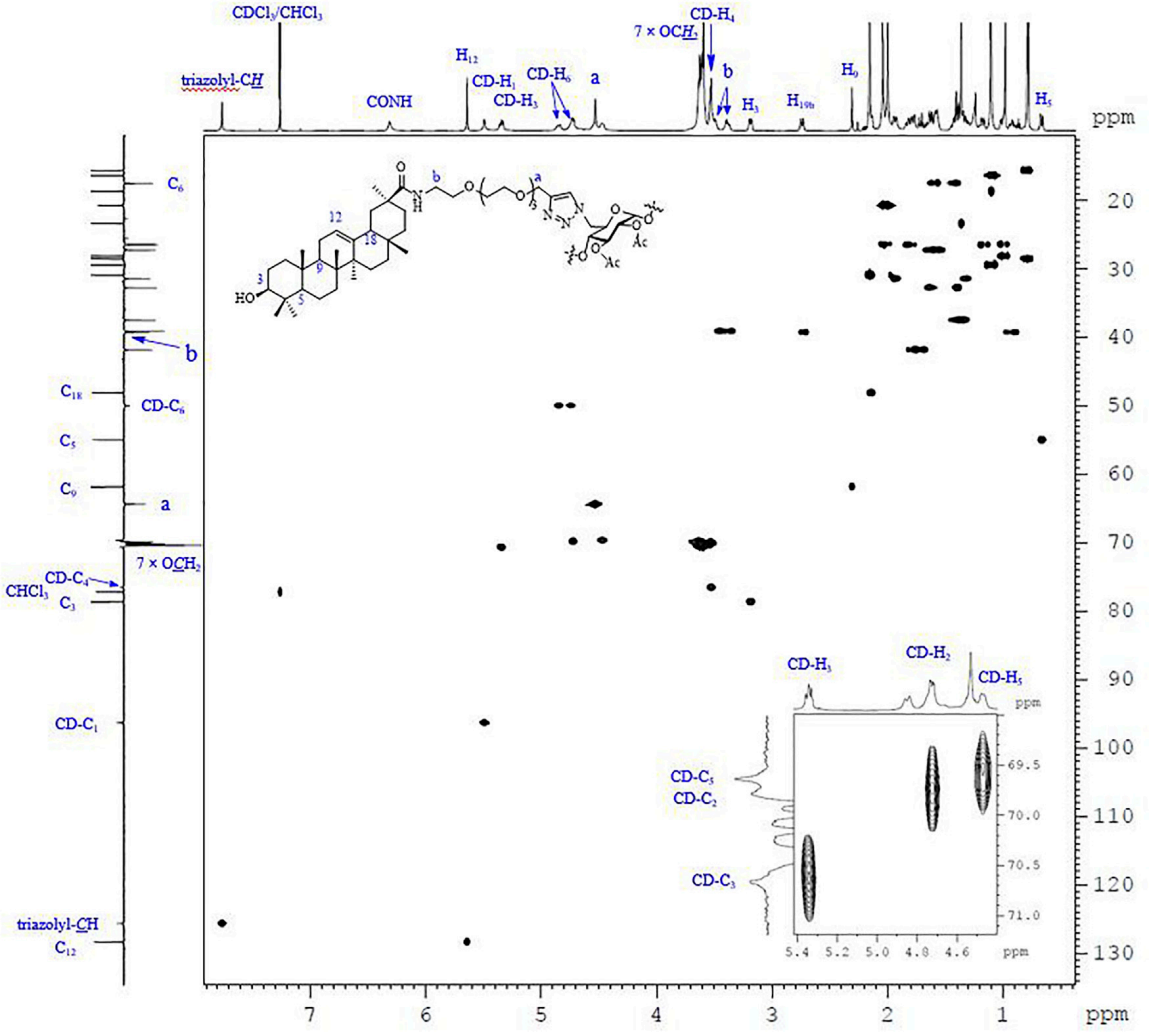
The 600 MHz HSQC spectrum of **23** recorded in CDCl_3_ at 298 K, with the 1D ^1^H and ^13^C NMR spectra along the top and the side, respectively.

Except for those signals of the linkers, the heptavalent conjugates were similar in their NMR spectra, and Figure 3 represents the HSQC spectrum of conjugate **23** as an example. The low-field regions of the conjugate were relatively well-resolved and fully assigned. In the aromatic region, the signal at *δ* 7.76 ppm was assigned to triazole-CH according to the ^1^H-^13^C correlation spectra, indicating that it was indeed connected by the triazole linker. The assignment of NH proton at *δ* 6.31 ppm was confirmed as there was no correlation for NH by an HSQC spectral analysis. The low-field peak at *δ* 5.64 ppm, referring to 1H, was assigned to H_12_, and one carbon appearing at *δ* 128.35 ppm should be assigned to C_12_ of GA. With a *C*_7_-symmetry in the molecule, compound **23** showed only one set of CD-H_1_ signal appearing at 5.49 ppm and one CD-C_1_ signal appearing at 96.29 ppm. A similar observation was also made for other proton and carbon signals of the *β*-CD unit, and the chemical shift data are summarized in Table 1. As C_b_ was connected with nitrogen rather than oxygen to which other carbons were bonded in the tetraethylene glycol linker, the chemical shift of C_b_ in ^13^C NMR was at the highest field (NH *C* H_2_ *vs* O *C* H_2_). In addition, as C_a_ was connected with triazole, the chemical shift of H_a_ in ^1^H NMR was at the lowest field among the eight -OCH_2_- groups. The MALDI-TOF MS of compound **23** showed a sodium adduct ion [M + Na]^+^ at *m/z* 6709.6 (Calcd. For C_357_H_546_N_28_NaO_91_, 6709.4), which also confirmed that it was a heptavalent conjugate.

**TABLE 1:** Chemical shifts of ^1^H and ^13^C NMR of *β*-CD unit in conjugate **23**.

^1^H NMR (ppm)		^13^C NMR (ppm)	
CD-H_1_	5.64	CD-C_1_	96.29
CD-H_2_	4.73	CD-C_2_	69.81
CD-H_3_	5.34	CD-C_3_	70.67
CD-H_4_	3.53	CD-C_4_	76.51
CD-H_5_	4.47	CD-C_5_	69.64
CD-H_6_	4.85, 4.75	CD-C_6_	49.97

### 3.3 *In Vitro* Cytotoxicity of Heptavalent β-CD-GA Conjugates

It has been reported that GA **2** and some of its derivatives are cytotoxic in cancer cells (Satomi et al., 2005; Lallemand et al., 2011). In this study, the cytotoxicity of heptavalent *β*-CD-GA conjugates **21–28** and **33–38** was first determined in MDCK cells using the CellTiter-Glo^®^ luminescent cell viability assay. A decrease in the viability of the MDCK cells was observed in a dose-dependent manner upon treatment of PTX (Supplementary Figure S3). Treatment of cells with **2** for 48 h resulted in decreased viability to 82.8% for the 25 *μ*M concentration and no viability for the 10 μM concentration, indicating that **2** had weak cytotoxicity to cells and agreed with previously reported results that **2** shows weak cytotoxicity to eight cancer cell lines with IC_50_ values between 70.48 and 136.40 μM (Gao et al., 2014). For all the tested conjugates, except that **25** and **28** showed weak cytotoxicity to uninfected MDCK cells with the reduced viability to 85.6% and 87.9%, respectively, at a high concentration of 25 *μ*M, no significant cytotoxicity for the other conjugates was observed at concentrations of 10 and 25 μM.

### 3.4 Structure-Activity Relationships of Heptavalent β-CD-GA Conjugates

Concerning the fact that the use of multivalent sialopeptides as potential inhibitors of influenza infections has been proved to be a useful approach (Mammen et al., 1998; Marra et al., 2008), our interest was aimed at evaluating the inhibitory effect of the heptavalent GA conjugates **21–28** and **33–38** by microscopic examination of the viral CPE at two days post infection (Noah et al., 2007). Oseltamivir (OSV) and DMSO were used as positive and negative controls, respectively.

**FIGURE 4:**
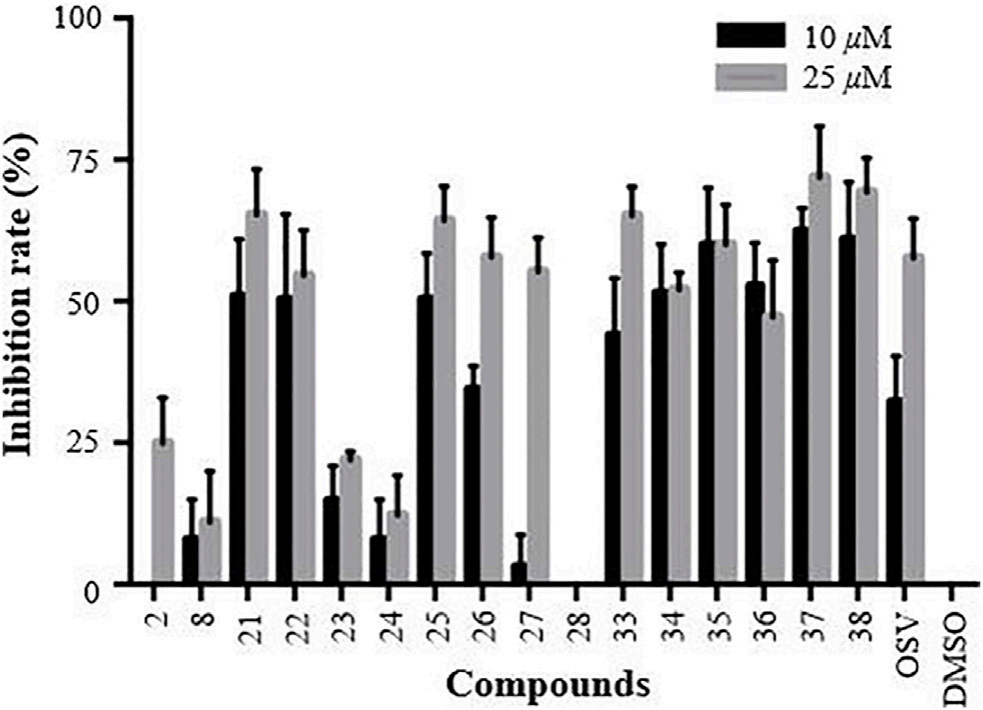
CPE-based primarily screening of GA (**2**), *β*-CD (**8**), and their heptavalent *β*-CD-GA conjugates **21–28** and **33–38**. MDCK cells were utilized as the host cells to test A/WSN/33 virus infection; OSV and DMSO acted as positive and negative controls, respectively. Error bars indicate standard deviations of triplicate experiments.

The primarily screening results are shown in Figure 4. A careful analysis and comparison of the data allowed us to reveal some interesting SAR trends. Regarding the OEG linkers between the triazole ring-substituted GA and *β*-CD scaffold, we found that the anti-influenza activity of conjugates **21–24** was decreased in the order as follows: **21** > **22**>>**23** > **24**. Similar results were also observed for conjugates **25–28**, indicating that a length of four atoms was found to be optimal for the inhibitory activity (**21** and **25**). Especially for conjugate **28**, the insertion of two more ethylene glycol spacers in **26** resulted in the loss of activity under 25 μM, suggesting the importance of the length of the linker between GA and *β*-CD for their anti-influenza activity. Concerning the nature of the linker’s heteroatom, the replacement of the oxygen of ethylene glycol in conjugates **21** and **25** by methylene (**33** and **34**) showed a slightly weaker or similar antiviral activity. Interestingly, the increase of the rigidity of the linker by further replacement of the four methylene with aromatic benzene at positions at 1,4 (**35** and **36**) still retained reasonable anti-influenza activity. Surprisingly, the inserting of a piperazine space in the alkyl liner caused a substantial increase in the activity of conjugates **37** and **38** (relative to **33** and **34**, respectively), which were proved to be the most two potent multivalent conjugates.

After the preliminary screening at two concentrations of 10 and 25 μM, 11 conjugates (**21–22**, **25–27** and **33–38**) with the inhibition rate of more than 50% at a concentration of 25 μM were selected to perform in dose response assays, and the concentrations required to inhibit viral replication by 50% (IC_50_) are summarized in Table 2. In addition, no significant toxicity was observed for the 10 conjugates at concentrations up to 100 *μ*M. Based on these data, we investigated in detail the potential of a representative heptavalent conjugate **37**.

**TABLE 2:** *In vitro* anti-influenza A virus activity of GA **(2)**, β-CD **(8)** and their heptavalent conjugates **21–22, 25–27** and **33–38** ^a^.

Compound	IC_50_, μM^b^	CC_50_, μM	SI	Compound	IC_50_, μM	CC_50_, *μ*M	SI
**2**	NA^c^	85 ± 6.8	**—**	**33**	12.1 ± 0.31	>100	**>**8.3
**8**	NA	ND^d^	**—**	**34**	9.03 ± 0.50	>100	**>**11.1
**21**	6.64 ± 0.31	>100	**>**15.1	**35**	20.7 ± 1.02	>100	**>**4.8
**22**	7.70 ± 0.74	>100	**>**13.0	**36**	11.0 ± 0.87	>100	**>**9.1
**25**	6.96 ± 0.39	>100	**>**14.4	**37**	2.86 ± 0.22	>100	**>**35.0
**26**	8.14 ± 0.11	>100	**>**12.3	**38**	4.89 ± 0.15	>100	**>**20.4
**27**	27.02 ± 0.11	>100	**>**3.7				

a Using influenza A/WSN/33 (H1N1) strain.

b The concentration required for a test compound to reduce the virus-induced CPE, by 50% relative to the virus control was expressed as IC_50_.

c No activity.

d Not detectable.

The CPE produced by the influenza A/WSN/33 virus in MDCK cells and its abrogation by treatment with **37** were further confirmed by direct observation under a microscope. No cytotoxicity was observed for **37** when incubated with the MDCK cells for 48 h (Supplementary Figures S4A vs S4B), while **37** significantly reduced the CPE induced by influenza A/WSN/33 virus infection (Supplementary Figures S4C vs S4D).

### 3.5 Hemagglutination Inhibition Assay

**FIGURE 5:**
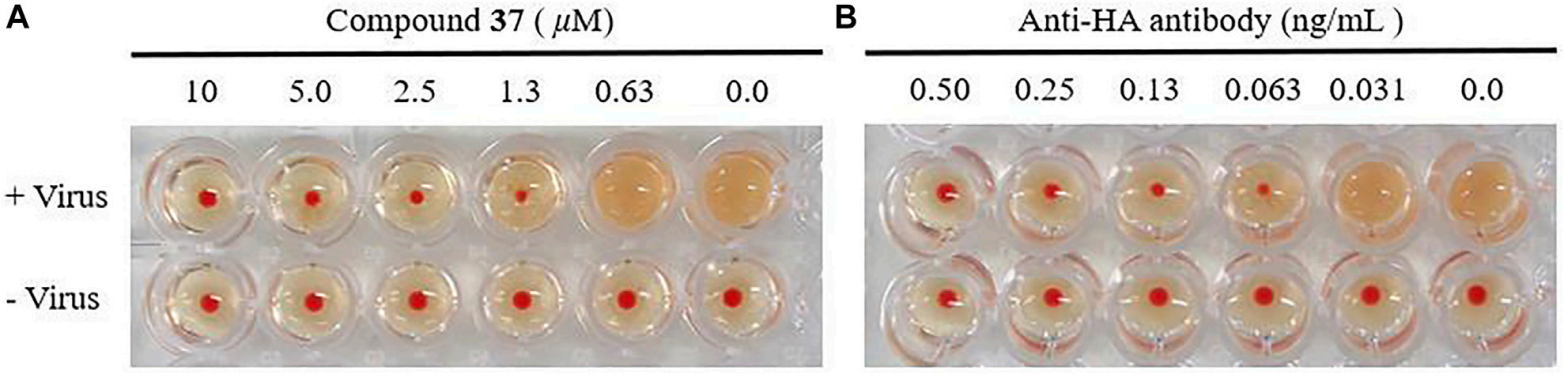
Effect of the heptavalent compound **37 (A)** and anti-HA antibody **(B)** on HA by HI assay. The chicken red blood cells (cRBCs) (1% v/v in saline) were incubated with influenza A/WSN/33 at 2 × HA with various concentrations of **37** (two-fold serial dilution from 10 μM) or anti-HA antibody (two-fold serial dilution from 0.50 ng/ml).

With the confirmation of the antiviral activity of the heptavalent conjugates, we were interested in identifying their molecular target. It is well known that the viral envelop glycoprotein HA plays a critical role in the initial step of influenza virus entry into host cells. Therefore, the HI assay was designed to detect whether the heptavalent conjugates could interfere with the interaction between HA and its sialic acid receptors. Twofold dilutions of the heptavalent conjugate **37** (from 10 μM) or anti-HA antibody (from 0.50 ng/ml)-treated virus were incubated with cRBC, and agglutination was observed (Figure 5). In addition, **37** completely inhibited influenza A/WSN/33 (H1N1) virus-induced agglutination of cRBC in a dose-dependent manner at concentrations of 1.3 μM or more. Similar results were observed for the anti-HA antibody at concentrations of 0.063 ng/ml or more. These results suggested that the heptavalent conjugates **37** shared the same target of the anti-HA antibody, thus interfering with virus–receptor interaction.

### 3.6 Determination of the K_D_ for the Interaction Between the Heptavalent Conjugate 37 With HA Protein

**FIGURE 6:**
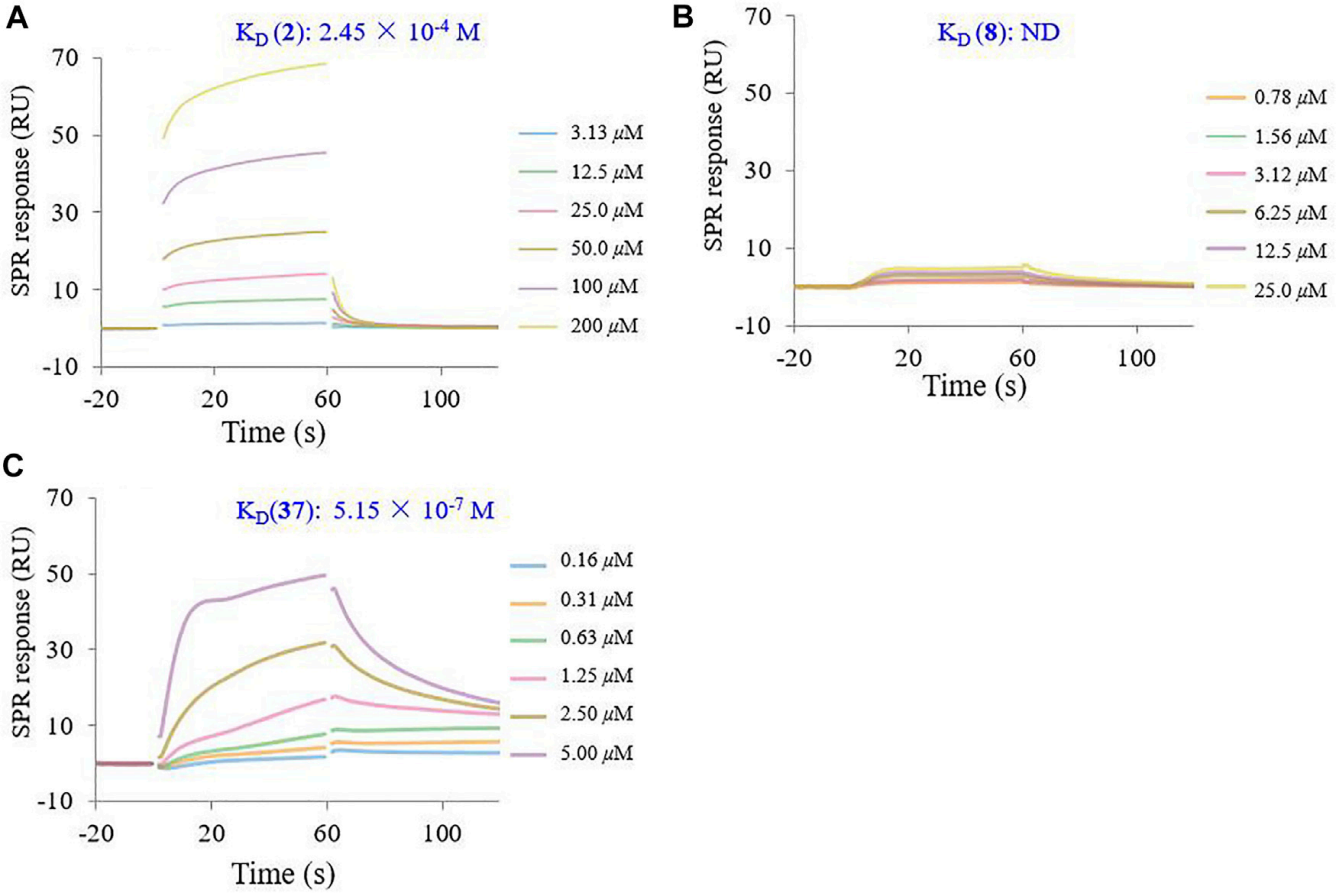
SPR inhibition assay sensor grams showing response to influenza HA protein in the presence of varying concentrations of GA (**2**) **(A)**, *β*-CD (**8**) **(B)**, and their heptavalent conjugates **37** **(C)**. The binding kinetics was analyzed by the BIA evaluation 1.0.5 software with 1:1 (Langmuir) binding mode.

To determine the binding affinity of conjugate **37** for the HA protein, SPR experiments were performed on a Biacore 8K instrument. The SPR response was reported in the resonance unites (RU). Sensor grams were analyzed with a 1:1 (Langmuir) binding mode. As shown in Figure 6, the binding of recombinant influenza HA to GA (**2**) exhibited typical weak affinity binding with a calculated equilibrium dissociation constant (*K*_D_) value of 2.45 × 10^−4^ M, while *β*-CD (**8**) did not bind to the HA protein at the same concentration. However, the multivalent conjugate **37** showed a strong binding affinity to the HA protein within the concentration range 0.16–5.00 μM. The calculated K_D_ value for conjugate **37** was 5.15 × 10^−7^ M, which seemed to be too large by about 470-fold of magnitude to that of **2** (Figures 6A vs 6C). In addition, kinetic analysis revealed that changes in the multivalent conjugate and HA protein not only affected the association rate constant (*K*_a_), but also affected the dissociation rate constant (*K*_d_) (Supplementary Table S1).

Compared with our previous study in which multiple pentacyclic triterpenes attached directly onto the CD scaffold *via* 1,2,3-triazolyl group (Xiao et al., 2016), the insertion of a suitable linker between them will be helpful for the binding of multiple pentacyclic triterpenes with their target. To test the feasibility of the strategy, various types of linkers including flexible linear alkyl/ether chain combinations between 4 and 19 atoms, a rigid aromatic space chain and semi-rigid charged piperzine chain have been successfully introduced in monovalent β-CD-GA conjugates in our recent study (Liang et al., 2019), which allowed us to further design and synthesis of the second-generation multivalent pentacyclic triterpene-β-CD conjugates. This well-established method will provide an alternative approach to the construction of novel multivalent derivatives bearing different linkers based on natural or chemically modified CD scaffolds. Combined with our previous studies, we found that the pentacyclic triterpene pharmacophore, the CD scaffold, and the linkers between the two parts were all affected by their anti-influenza activity.

## 4 Conclusion

In this work, a series of multiple GA-functionalized *β*-CD conjugates with different linkers were synthesized and characterized. Except those two conjugates (**25** and **28**) which showed weak cytotoxicity to MDCK cells at a concentration of 25 μM, all other conjugates showed no significant cytotoxicity based on an alamarBlue assay. When presented multivalently, seven *β*-CD-GA conjugates (**21**, **22**, **25**, **26**, **34**, **37**, and **38**) showed strong anti-influenza properties with IC_50_ values at a micromolar level (IC_50_: 2.86–9.03 μM). By using HI and SPR assays, conjugate **37** showed specific binding to the HA protein with the *K*_D_ value at 515 nM, which was about 470-fold potent to its parent compound GA (**2**). Taken together, the results reported here for multivalent *β*-CD-GA conjugates demonstrated that multivalent displays of the anti-influenza entry agent afford macromolecules with a remarkable activity and a strong binding to the HA protein relative to their parent compound alone, and multivalency based on *β*-CD scaffold is a promising alternative to the currently available treatments for the management of viral infection by blocking virus entry into host cells.

## Data Availability

The original contributions presented in the study are included in the article/Supplementary Material; further inquiries can be directed to the corresponding author.
