# Supplementary material for: Novel β-Cyclodextrin-Based Heptavalent Glycyrrhetinic Acid Conjugates: Synthesis, Characterization, and Anti-Influenza Activity
Source: Front Chem. 2022 Apr 12;10:836955. doi: 10.3389/fchem.2022.836955 (PMC9039011; doi:10.3389/fchem.2022.836955)

Supplementary Data

Novel *β*-cyclodextrin-based heptavalent glycyrrhetinic acid conjugates: Synthesis, characterization and anti-influenza activity

Shuobin Liang[1†], Xinyuan Ma[1†], Man Li[1], Yanliang Yi[1], Qianqian Gao[1], Yongmin Zhang[4], Lihe Zhang[1], Demin Zhou[1,2,3], Sulong Xiao[1*]

[1] *State Key Laboratory of Natural and Biomimetic Drugs, School of Pharmaceutical Sciences, Peking University, Beijing 100191, China*

[2] *Institute of Chemical Biology, Shenzhen Bay Laboratories, Shenzhen 518132, China*

[3] *Ningbo Institute of Marine Medicine, Peking University, Ningbo 315010, China*

[4] *Sorbonne Université, Institut Parisien de Chimie Moléculaire, CNRS UMR 8232, 4 place Jussieu, 75005 Paris, France*

1. **Figure S1** and **Figure S2**. FTIR spectra of compounds **2** and **11** S3
2. **Figure S3**. The *in vitro* cytotoxic screening of GA (**2**), *β*-CD (**8**) as well as their heptavalent *β*-CD-GA conjugates **21-28** and **33-38** S4
3. **Figure S4.** Compound **37** inhibited virus-induced CPE in MDCK cells. S4
4. **Table S1**. Binding kinetics of compounds **2**, **8** and their conjugate **37** to HA protein S5
5. Synthesis and structure characterization of the new compounds **21-28** and **33-38** S6
6. Selected NMR and MS spectra of conjugates **21-28** and **33-38** S15

Spectra	Page
^{1}H NMR of compound **21**	S14
^{13}C NMR of compound **21**	S14
HSQC of compound **21**	S15
MALDI-TOF of compound **21**	S15
^{1}H NMR of compound **22**	S16
^{13}C NMR of compound **22**	S16
HSQC of compound **22**	S17

MALDI-TOF of compound **22**	S17
^{1}H NMR of compound **23**	S18
^{13}C NMR of compound **23**	S18
HSQC of compound **23**	S19
MALDI-TOF of compound **23**	S19
^{1}H NMR of compound **24**	S20
^{13}C NMR of compound **24**	S20
HSQC of compound **24**	S21

MALDI-TOF of compound **24**	S21
^{1}H NMR of compound **25**	S22
^{13}C NMR of compound **25**	S22
HSQC of compound **25**	S23
MALDI-TOF of compound **25**	S23
^{1}H NMR of compound **26**	S24
^{13}C NMR of compound **26**	S24
HSQC of compound **26**	S25
MALDI-TOF of compound **26**	S25
^{1}H NMR of compound **27**	S26
^{13}C NMR of compound **27**	S26
HSQC of compound **27**	S27
MALDI-TOF of compound **27**	S27
^{1}H NMR of compound **28**	S28
^{13}C NMR of compound **28**	S28
HSQC of compound **28**	S29
MALDI-TOF of compound **28**	S29
^{1}H NMR of compound **33**	S30
^{13}C NMR of compound **33**	S30
HSQC of compound **33**	S31
MALDI-TOF of compound **33**	S31
^{1}H NMR of compound **34**	S32

^{13}C NMR of compound **34**	S32
HSQC of compound **34**	S33
MALDI-TOF of compound **34**	S33
^{1}H NMR of compound **35**	S34
^{13}C NMR of compound **35**	S34
HSQC of compound **35**	S35
MALDI-TOF of compound **35**	S35
^{1}H NMR of compound **36**	S36
^{13}C NMR of compound **36**	S36
HSQC of compound **36**	S37
MALDI-TOF of compound **36**	S37
^{1}H NMR of compound **37**	S38
^{13}C NMR of compound **37**	S38
HSQC of compound **37**	S39
MALDI-TOF of compound **37**	S39
^{1}H NMR of compound **38**	S40
^{13}C NMR of compound **38**	S40
HSQC of compound **38**	S41
MALDI-TOF of compound **38**	S41

1. **Figure S1** and **Figure S2.** FTIR spectra of compounds **2** and **11**

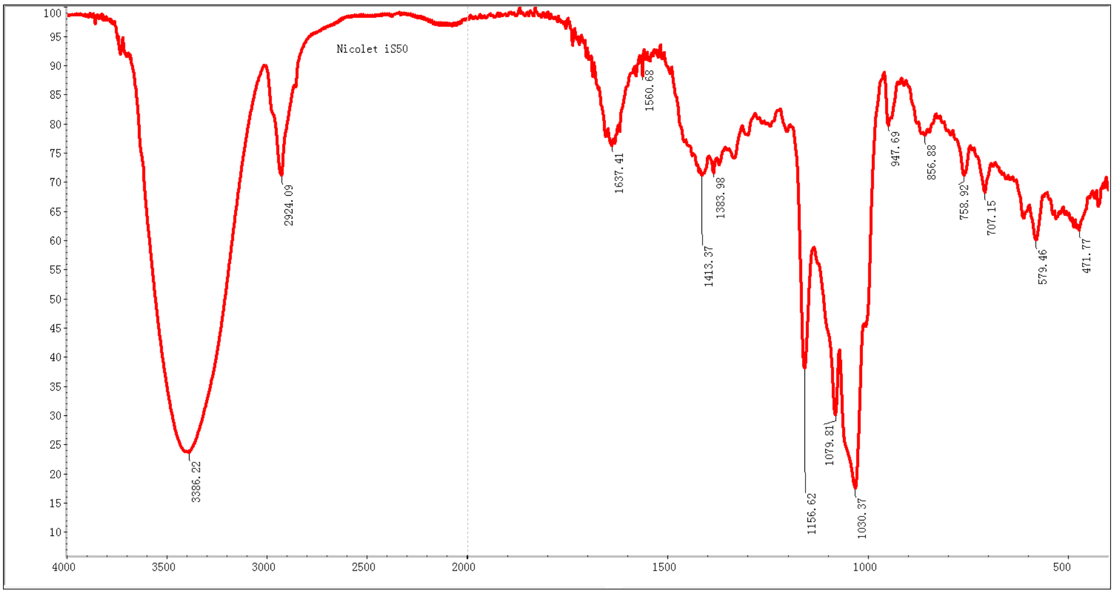

Figure S1. FTIR spectrum of compound **11**

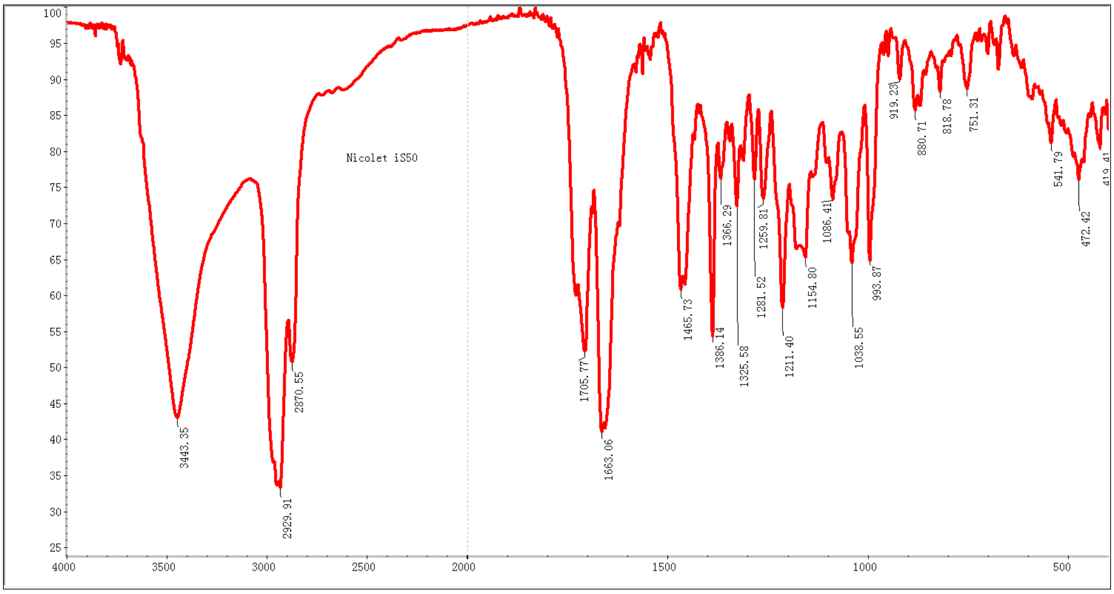

Figure S2. FTIR spectrum of compound **2**

2. **Figure S3**. The *in vitro* cytotoxic screening of GA (**2**), β-CD (**8**) as well as their heptavalent β-CD-GA conjugates **21-28** and **33-38**.

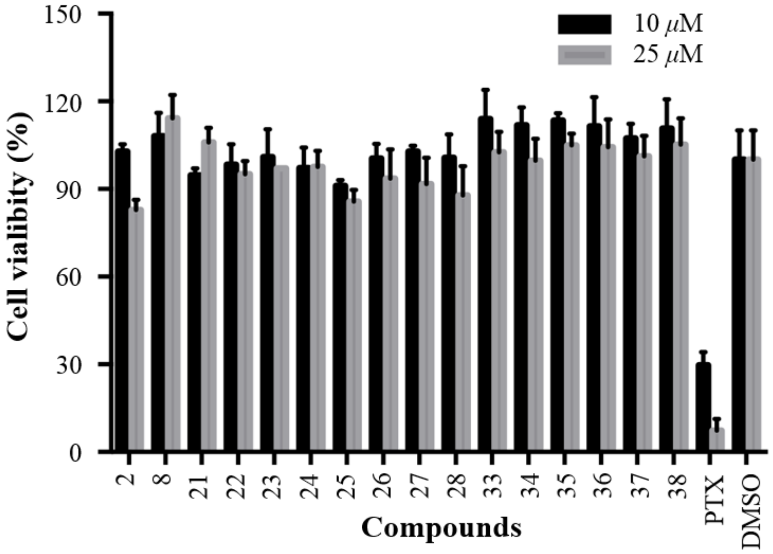

Fig. S3. The *in vitro* cytotoxic screening of GA (**2**), β-CD (**8**) as well as their heptavalent β-CD-GA conjugates **21-28** and **33-38** against MDCK cells using CellTiter-Glo® luminescent cell viability Assay. DMSO and PTX were used as negative and positive control, respectively. Error bars indicate standard deviations of triplicate experiments.

3. **Figure S4.** Compound **37** inhibited virus-induced CPE in MDCK cells.

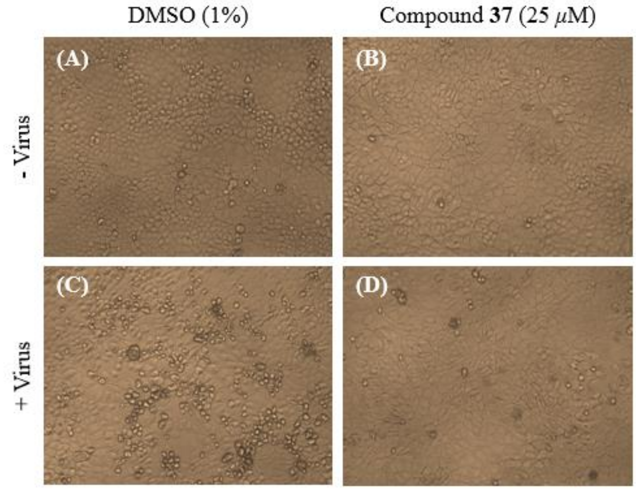

Fig. S4. Compound **37** inhibited virus-induced CPE in MDCK cells. Cells were seeded into 96-well plates, incubated overnight and infected with influenza virus (MOI = 0.1) suspended in DMEM supplemented with 1% FBS, 2 μg/mL TPCK-treated trypsin, 25 μM compound **37**, and a final DMSO concentration of 1% in each well. The CPE reduction was observed 48 h post-infection under a microscope. DMSO was used as a negative control.

4. **Table S1**. Binding kinetics of compounds **2**, **8** and their conjugate **37** to HA protein

Compd	K_a (M^{-1} sec^{-1})	K_d (sec^{-1})	K_D(M)
**2**	1.98×10^{3}	4.86×10^{-1}	2.45×10^{-4}
**8**	-	-	-
**37**	2.31×10^{4}	1.19×10^{-2}	5.15×10^{-7}

5.Synthesis and structure characterization of the new compounds **21-29** and **33-38**

*5.1 Synthesis of heptakis (2,3-di-O-acetyl-6-deoxy-6-(4-(((3β-hydroxyolean-11-oxo-12-en-30-oyl)-aminoethyl)oxymethyl)-1H-1,2,3- triazol-1-yl))-β-CD (**21**)*

Prepared from **11** and **17** according to general procedure A, the residue was purified by flash chromatography (eluent: CH_2Cl_2:MeOH = 15:1) to afford **21** as a white solid in 59% yield. R_f= 0.45 (eluent: CH_2Cl_2:MeOH = 8:1); ^{1}H NMR (600 MHz, $CDCl_3$): δ 8.08 (s, 7H), 7.03 (br s, 7H), 5.63 (s, 7H), 5.43 (s, 14H), 4.76–4.46 (m, 12H), 3.61–3.46 (m, 10H), 3.03 (s, 7H), 2.72 (s, 7H), 2.29 (s, 7H), 2.07 (m, 7H), 2.03 (m, 28H), 2.00 (m, 21H), 1.98 (m, 7H) 1.82 (m, 14H), 1.74–1.70 (m, 14H), 1.62–1.57 (m, 14H), 1.45 (m, 7H), 1.41–1.40 (m, 14H), 1.37 (s, 21H, 7 × CH_3), 1.32–1.30 (m, 14H), 1.18 (d, J = 11.8 Hz, 7H), 1.11 (s, 42H, 14 × CH_3), 1.08 (s, 21H, 7 × CH_3), 1.01–0.99 (m, 7H), 0.96 (s, 21H, 7 × CH_3), 0.78 (s, 42H, 14 × CH_3), 0.77 (m, 7H), 0.58 (d, 7H, J = 14.2 Hz); ^{13}C NMR (150 MHz, $CDCl_3$): δ 199.84, 175.82, 170.39, 169.15, 144.17, 127.75, 126.67, 96.90, 78.86, 77.18, 70.59, 70.05, 69.25, 64.24, 61.91, 55.37, 50.66, 48.49, 45.43, 43.35, 43.15, 41.63, 40.19, 39.20, 37.44, 37.05, 32.72, 31.81, 31.33, 28.95, 28.58, 28.30, 27.26, 26.58, 26.32, 23.39, 20.74, 18.70, 17.43, 16.56, 15.63; MALDI-TOF MS m/z: 5784.8 $[M+Na]^+$.

*5.2 Synthesis of heptakis (2,3-di-O-acetyl-6-deoxy-6-(4-((((3β-hydroxyolean-11-oxo-12-en-30-oyl)-aminoethyl)oxyethyl)oxymethyl)-1H-1,2,3- triazol-1-yl))-β-CD (**22**)*

Prepared from **11** and **18** according to general procedure A, the residue was purified by flash chromatography (eluent: CH_2Cl_2:MeOH = 15:1) to afford **22** as a white solid in 88% yield. R_f= 0.35 (eluent: CH_2Cl_2:MeOH = 10:1); ^{1}H NMR (600 MHz, $CDCl_3$): δ 7.83 (s, 7H), 6.68 (s, 7H), 5.64 (s, 7H), 5.47 (s, 7H), 5.41 (t, 7H, J = 8.9 Hz), 4.85 (s, 7H), 4.80 (s, 7H), 4.71 (d, 7H, J = 7.7 Hz), 4.53 (s, 7H), 3.62–3.46 (m, 56H), 3.29 (s, 7H), 3.14 (dd, 7H, J = 11.2, 4.1 Hz), 2.71 (td, 7H, J = 13.0, 2.7 Hz), 2.31 (s, 7H), 2.12 (br d, 7H, J = 13.8 Hz), 2.04 (m, 7H), 2.03, 2.01 (s, each 21H, 2 × $COCH_3$), 1.96 (br d, 7H, J = 11.9 Hz), 1.83–1.81 (m, 14H), 1.70 (t, 7H, J = 13.6 Hz), 1.63–1.57 (m, 14H), 1.45–1.33 (m, 35H), 1.36 (s, 21H, CH_3), 1.28 (dt, 7H, J = 13.7, 2.3 Hz), 1.17 (br d, 7H, J = 12.4 Hz), 1.10 (2 × s, 63H, 21 × CH_3), 1.00 (br d, 7H, J = 10.6 Hz), 0.98 (s, 21H, 7 × CH_3), 0.87 (t, 7H, J = 12.9 Hz), 0.78 (s, 42H, 14 × CH_3), 0.64 (d, 7H, J = 11.6 Hz); ^{13}C NMR (150 MHz, $CDCl_3$): δ 199.86, 175.85, 170.35, 169.54, 169.26, 144.59, 128.18, 125.86, 96.50, 78.69, 76.82, 70.47, 70.11, 70.02, 69.91, 69.82, 69.64, 64.24, 61.83, 55.10, 50.01, 48.18, 45.40, 43.48, 43.14, 41.64, 39.63, 39.17, 39.05, 37.46, 37.05, 32.72, 31.83, 31.35, 29.33, 28.49, 28.22, 27.14, 26.53, 26.35, 23.33,

20.75, 20.71, 18.66, 17.44, 16.48, 15.62; MALDI-TOF MS m/z: 6093.1 $[M+Na]^+$.

*5.3 Synthesis of heptakis (2,3-di-O-acetyl-6-deoxy-6-(4-(((3β-hydroxy-11-oxo-olean-12-en-30-oyl)-amino)-3,6,9,12-tetraoxatridecan-13-yl)-1H-1,2,3-triazol-1-yl))-β-CD (**23**)*

Prepared from **11** and **19** according to general procedure A, the residue was purified by flash chromatography (eluent: CH_2Cl_2:MeOH = 10:1) to afford **23** as a white solid in 54% yield. R_f = 0.15 (eluent: CH_2Cl_2:MeOH = 10:1); ^1H NMR (600 MHz, $CDCl_3$): δ 7.76 (s, 7H), 6.31 (br s, 7H), 5.64 (s, 7H), 5.49 (s, 7H), 5.34 (t, 7H, J = 8.7 Hz), 4.85 (d, 7H, J = 13.0 Hz), 4.75–4.71 (m, 14H), 4.53 (t, 14H, J = 12.8 Hz), 4.47 (d, 7H, J = 8.1 Hz), 3.63–3.49 (m, 112H), 3.40–3.36 (m, 7H), 3.19 (dd, 7H, J = 11.3, 4.6 Hz), 2.74 (td, 7H, J = 13.5, 3.3 Hz), 2.31 (s, 7H), 2.14-2.13 (m, 7H), 2.04 (s, 21H), 2.03 (m, 7H), 2.00 (s, 27H), 1.93 (d, 7H, J = 11.3 Hz), 1.82 (dt, 7H, J = 13.6, 4.2 Hz), 1.78 (d, 14H, J = 14.3 Hz), 1.71 (d, 7H, J = 13.7 Hz), 1.68–1.57 (m, 28H), 1.45–1.30 (m, 35H), 1.36 (s, 21H, 7 × CH_3), 1.18 (d, 7H, J = 12.7 Hz), 1.11 (s, 63H, 21 × CH_3), 1.01 (d, 7H, J = 12.8 Hz), 0.98 (s, 21H, 7 × CH_3), 0.93 (dt, 7H, J = 12.9, 2.9 Hz), 0.79, 0.78 (s, each 21H, 14 × CH_3), 0.67 (d, 7H, J = 11.5 Hz); ^{13}C NMR (150 MHz, $CDCl_3$): δ 199.82, 175.74, 170.33, 169.33, 169.14, 144.78, 128.35, 125.62, 96.29, 78.61, 76.51, 70.67, 70.45, 70.39, 70.35, 70.16, 70.00, 69.88, 69.81, 69.64, 64.38, 61.81, 54.97, 49.97, 48.10, 45.34, 43.53, 43.16, 41.81, 39.26, 39.10, 39.09, 37.46, 37.03, 32.74, 31.85, 31.42, 29.42, 28.51, 28.13, 27.24, 26.48, 26.39, 23.37, 20.72, 20.68, 18.65, 17.45, 16.34, 15.61; MALDI-TOF MS m/z: 6709.6 $[M+Na]^+$.

*5.4 Synthesis of Heptakis (2,3-di-O-acetyl-6-deoxy-6-(4-(((3β-hydroxy-11-oxo-olean-12-en-30-oyl)-amino)-3,6,9,12,15,18-hexaoxanonadecan-19-yl)-1H-1,2,3-triazol-1-yl))-β-CD (**24**)*

Prepared from **11** and **20** according to general procedure A, the residue was purified by flash chromatography (eluent: CH_2Cl_2:MeOH = 10:1) to afford **24** as a white solid in 62% yield. R_f= 0.20 (eluent: CH_2Cl_2:MeOH = 8:1); ^1H NMR (400 MHz, $CDCl_3$): δ 7.78 (s, 7H), 6.63 (s, 7H), 5.65 (s, 7H), 5.49 (s, 7H), 5.34 (s, 7H), 4.84 (s, 7H), 4.73–4.71 (m, 14H), 4.55 (s, 14H), 4.48 (s, 7H), 3.62–3.47 (m, 168H), 3.43–3.39 (m, 7H), 3.14 (dd, 7H, J = 11.0, 5.0 Hz), 2.75 (br d, 7H, J = 13.0 Hz), 2.32 (s, 7H), 2.15 (d, 7H, J = 12.2 Hz), 2.05 (s, 21H, 7 × $COCH_3$), 2.03 (m, 7H), 2.00 (s, 21H, 7 × $COCH_3$), 1.94 (d, 7H, J = 11.8 Hz), 1.85–1.57 (m, 49H), 1.46–1.31 (m, 35H), 1.37 (s, 21H, 7 × CH_3), 1.18 (d, 7H, J = 13.9 Hz), 1.11 (s, 63H, 21 × CH_3), 1.02 (m, 7H), 0.99 (s, 21H, 7 × CH_3), 0.94 (m, 7H), 0.79 (s, 42H, 21 × CH_3), 0.68 (d, 7H, J = 11.8 Hz); ^{13}C NMR (100 MHz, $CDCl_3$): δ 199.91, 175.81, 170.35, 169.37, 169.19, 144.75, 128.37, 125.67, 96.33, 78.62, 76.40, 70.63, 70.57,

70.40, 70.13, 69.78, 69.76, 69.66, 69.59, 64.35, 61.80, 54.94, 49.98, 48.06, 45.33, 43.54, 43.15, 41.80, 39.21, 39.11, 39.09, 37.46, 37.02, 32.73, 31.84, 31.41, 29.6, 28.53, 28.11, 27.24, 26.46, 26.38, 23.37, 20.74, 20.70, 18.64, 17.45, 16.35, 15.61; MALDI-TOF MS m/z: 7326.5 $[M+Na]^+$.

*5.5 Synthesis of heptakis (6-deoxy-6-(4-(((3β-hydroxyolean-11-oxo-12-en-30-oyl)-aminoethyl)oxymethyl)-1H-1,2,3-triazol-1-yl))-β-CD (**25**)*

Prepared from **21** according to general procedure B, the residue was purified by RP flash chromatography (eluent: methanol) to afford **25** as a white solid in 73% yield. ^{1}H NMR (600 MHz, CD_3OD:$CDCl_3$ = 2:1): δ 7.96 (s, 7H), 5.62 (s, 7H), 5.12 (d, 7H, J = 2.5 Hz), 4.55–4.47 (m, 21H), 4.38 (dd, 7H, J = 8.4, 5.5 Hz), 4.16 (br s, 7H), 3.87 (t, 7H, J = 9.2 Hz), 3.53–3.52 (m, 14H), 3.47–3.41 (m, 14H), 3.30 (m, 7H, overlap with CD_3OD), 3.28 (dd, 7H, J = 18.3, 9.3 Hz), 3.16 (dd, 7H, J = 11.4, 4.4 Hz), 2.70 (d, 7H, J = 12.9 Hz), 2.38 (s, 7H), 2.13–2.07 (m, 14H), 1.94 (d, 7H, J = 5.0 Hz), 1.89 (d, 7H, J = 13.0 Hz), 1.83 (t, 7H, J = 12.3 Hz), 1.70–1.64 (m, 21H), 1.61 (d, 7H, J = 12.8 Hz), 1.53–1.52 (m, 7H), 1.43–1.36 (m, 35H), 1.39 (s, 21H, 7 × CH_3), 1.21 (d, 7H, J = 12.8 Hz), 1.11, 1.10, 1.09 (s, each 21H, 21 × CH_3), 1.02 (d, 7H, J = 12.3 Hz), 0.98 (s, 21H, 7 × CH_3), 0.97 (m, 7H), 0.78, 0.77 (s, each 21H, 14 × CH_3), 0.71 (d, 7H, J = 11.5 Hz); ^{13}C NMR (150 MHz, CD_3OD:$CDCl_3$ = 2:1): δ 201.86, 178.18, 171.77, 145.13, 130.54, 128.83, 126.98, 103.09, 83.81, 79.05, 73.67, 73.18, 71.30, 70.01, 64.25, 62.79, 55.86, 51.48, 49.20, 46.38, 44.45, 44.21, 42.25, 40.21, 39.94, 39.78, 38.41, 37.96, 33.54, 32.63, 31.83, 29.65, 29.30, 28.58, 27.47, 27.29, 27.15, 23.88, 19.33, 18.29, 16.98, 16.24; MALDI-TOF MS m/z: 5195.4 $[M+Na]^+$, 5210.9 $[M+K]^+$

*5.6 Synthesis of heptakis (6-deoxy-6-(4-((((3β-hydroxyolean-11-oxo-12-en-30-oyl)-aminoethyl)oxyethyl)oxymethyl)-1H-1,2,3- triazol-1-yl))-β-CD (**26**)*

Prepared from **22** according to general procedure B, the residue was purified by RP flash chromatography (eluent: methanol) to afford **26** as a white solid in 82% yield. ^{1}H NMR (600 MHz, CD_3OD:$CDCl_3$ = 2:1): δ 7.99 (s, 7H), 5.65 (s, 7H), 5.16 (d, 7H, J = 2.9 Hz), 4.56–4.92 (m, 21H), 4.42 (dd, 7H, J = 9.7, 5.2 Hz), 4.21 (brs, 7H), 3.89 (t, 7H, J = 9.2 Hz), 3.66–3.61 (m, 14H), 3.59–3.57 (m, 14H), 3.52–3.48 (m, 14H), 3.45 (dd, 7H, J = 9.7, 3.6 Hz), 3.42 (t, 7H, J = 5.5 Hz), 3.32 (t, 7H, J = 5.6 Hz), 3.29 (m, 7H, overlap with CD_3OD), 3.17 (dd, 7H, J = 11.7, 4.4 Hz), 2.72 (dt, 7H, J = 13.4, 3.2 Hz), 2.40 (s, 7H), 2.16 (dd, 7H, J = 14.3, 4.0 Hz), 2.11 (dt, 7H, J = 13.3, 3.7 Hz), 1.95 (d, 7H, J = 6.6 Hz), 1.90 (d, 7H, J = 13.4 Hz), 1.85 (dt, 7H, J = 13.3, 3.5 Hz), 1.72–1.66 (m, 21H), 1.62 (br d, 7H, J = 13.6 Hz), 1.56–1.53 (m, 7H), 1.48–1.38 (m, 35H), 1.40 (s, 21H,), 1.23 (d, 7H, J

= 12.5 Hz), 1.12 (s, 42H, 14 × CH_3), 1.10 (s, 21H, 7 × CH_3), 1.03 (d, 7H, *J* = 13.3 Hz), 0.98 (s, 21H, 7 × CH_3), 0.97 (m, 7H), 0.81, 0.79 (s, each 21H, 14 × CH_3), 0.73 (d, 7H, *J* = 11.6 Hz);^{13}C NMR (150 MHz, CD_3OD:$CDCl_3$ = 2:1): *δ* 202.00, 178.29, 171.93, 145.32, 129.00, 127.17, 103.16, 83.84, 79.16, 73.92, 73.37, 71.35, 71.01, 70.78, 70.56, 64.81, 62.91, 55.99, 51.31, 46.48, 44.58, 44.33, 42.40, 40.25, 40.04, 40.03, 38.56, 38.09, 33.66, 32.73, 31.93, 29.63, 29.36, 28.64, 27.63, 27.41, 27.30, 23.91, 19.38, 18.42, 17.02, 16.30; MALDI-TOF MS m/z: 5504.9 $[M+Na]^+$, 5520.4 $[M+K]^+$.

*5.7 Synthesis of heptakis (6-deoxy-6-(4-(((3β-hydroxy-11-oxo-olean-12-en-30-oyl)-amino)-3,6,9,12-tetraoxatridecan-13-yl)-1H-1,2,3-triazol-1-yl))-β-CD (**27**)*

Prepared from **23** according to general procedure B, the residue was purified by RP flash chromatography (eluent: methanol) to afford **27** as a white solid in 86% yield. ^{1}H NMR (600 MHz, MeOD:$CDCl_3$ = 2:1): *δ* 7.91 (s, 7H), 5.64 (s, 7H), 5.13 (s, 7H), 4.64–4.52 (m, 28H, overlap with H_2O), 4.18 (br s, 7H), 3.87 (br s, 7H), 3.64–3.59 (m, 84H), 3.52 (t, 14H, *J* = 5.3 Hz), 3.45–3.42 (m, 14H), 3.37 (t, 7H, *J* = 5.2 Hz), 3.22 (br s, 7H), 3.16 (dd, 7H, *J* = 11.7, 4.5 Hz), 2.70 (dt, 7H, *J* = 10.3, 2.9 Hz), 2.37 (s, 7H), 2.15 (m, 7H, overlap with acetone), 2.07 (dt, 7H, J = 13.6, 4.1 Hz), 1.92 (d, 7H, *J* = 10.5 Hz), 1.87–1.85 (m, 7H), 1.83 (dt, 7H, *J* = 13.6, 4.2 Hz), 1.67–1.59 (m, 28H), 1.55–1.52 (m, 7H), 1.43–1.35 (m, 35H), 1.38 (s, 21H, 7 × CH_3), 1.21 (d, 7H, *J* = 14.8 Hz), 1.10 (s, 42H, 14 × CH_3), 1.09 (s, 21H, 7 × CH_3), 1.02 (d, 7H, *J* = 13.6 Hz), 0.97 (s, 21H, 7 × CH_3), 0.96 (m, 7H), 0.80, 0.77 (s, each 27H, 14 × CH_3), 0.70 (d, 7H, *J* = 11.5 Hz); ^{13}C NMR (150 MHz, MeOD:$CDCl_3$ = 2:1): *δ* 201.74, 177.79, 171.43, 144.99, 126.68, 102.68, 83.21, 78.83, 78.39, 73.45, 72.88, 70.95, 70.92, 70.64, 70.43, 70.35, 64.54, 62.54, 55.60, 50.95, 49.86, 48.80, 46.11, 44.21, 43.95, 42.06, 39.87, 39.75, 39.69, 38.12, 37.70, 33.33, 32.44, 32.38, 31.67, 29.54, 29.08, 28.43, 27.26, 27.04, 26.96, 23.77, 19.13, 18.04, 16.77, 16.06; MALDI-TOF MS m/z: 6122.1 $[M+Na]^+$.

*5.8 Synthesis of heptakis (6-deoxy-6-(4-(((3β-hydroxy-11-oxo-olean-12-en-30-oyl)-amino)-3,6,9,12,15,18-hexaoxanonadecan-19-yl)-1H-1,2,3-triazol-1-yl))-β-CD (**28**)*

Prepared from **24** according to general procedure B, the residue was purified by RP flash chromatography (eluent: methanol) to afford **28** as a white solid in 83% yield. ^{1}H NMR (600 MHz, MeOD:$CDCl_3$ = 2:1): *δ* 8.10 (s, 7H), 5.66 (s, 7H), 5.19 (br s, 7H), 4.56–4.47 (m, 21H), 4.36 (m, 7H), 4.20 (s, 7H), 3.92 (t, 7H, *J* = 8.9 Hz), 3.67–3.57 (m, 140H), 3.55 (t, 14H, *J* = 5.6 Hz), 3.51 (d, 7H, *J* = 10.0 Hz), 3.45 (td, 7H, *J* = 13.9, 5.3 Hz), 3.39–3.36 (m, 7H), 3.34 (t, 7H, *J* = 5.7 Hz) 3.18 (dd, 7H, *J* = 11.6, 4.1 Hz), 2.73 (td, 7H, *J* = 13.4, 3.0 Hz), 2.44 (s, 7H), 2.19–2.14 (m, 14H), 1.98–

1.96 (m, 7H), 1.91–1.85 (m, 14H), 1.72–1.68 (m, 21H), 1.64 (d, 7H, *J* = 14.9 Hz), 1.56–1.54 (m, 7H), 1.49–1.39 (m, 42H), 1.43 (s, 21H, 7 × CH_3), 1.25 (d, 7H, *J* = 11.7 Hz), 1.14 (s, 42H, 14 × CH_3), 1.12 (s, 21H, 7 × CH_3), 1.03 (t, 7H, *J* = 12.9 Hz), 1.01 (m, 7H), 1.00, 0.83, 0.81 (s, each 27H, 21 × CH_3), 0.77 (d, 7H, *J* = 11.6 Hz); ^{13}C NMR (150 MHz, MeOD:$CDCl_3$ = 2:1): δ 202.14, 178.67, 172.24, 145.35, 129.19, 127.89, 103.43, 84.46, 79.37, 74.18, 73.65, 71.77, 71.55, 71.50, 71.26, 71.18, 70.75, 64.70, 63.15, 56.21, 52.08, 49.57, 46.69, 44.82, 44.57, 42.66, 40.42, 40.36, 40.27, 38.82, 38.35, 33.86, 32.95, 32.07, 29.59, 29.42, 28.79, 27.89, 27.64, 27.50, 23.91, 19.46, 18.68, 17.11, 16.47; MALDI-TOF MS m/z: 6738.0 $[M+Na]^+$.

*5.9 Synthesis of N-(hex-5-yn-1-yl)-3β-hydroxy-11-oxo-olean-12-en-30-amide (**29**)*

To a solution of compound **12** (453 mg, 0.77 mmol) and hex-5-yn-1-amine (75.0 mg, 0.77 mmol) in DMF (20 mL), Na_2CO_3 (81 mg, 0.77 mmol) was added. The resulting solution was vigorously stirred for 24 h at room temperature. The solvent was removed by steaming. The residue was purified by column chromatography (eluent: petroleum ether:ethyl acetate = 3:1) to afford **29** as a white solid in 92% yield. m.p. 112-114 °C; R_f = 0.35 (petroleum ether:ethyl acetate = 1:1); m.p. 112-114; ^1H NMR (400 MHz, $CDCl_3$): δ 5.78 (t, 1H, *J* = 5.9 Hz), 5.63 (s, 1H), 3.34–3.24 (m, 2H), 3.19 (dd, 1H, *J* = 10.3, 3.8 Hz), 2.77–2.73 (m, 3H), 2.31 (s, 1H), 2.20 (dt, 2H, *J* = 6.8, 2.6 Hz), 2.13 (dd, 1H, *J* = 12.9, 4.2 Hz), 2.02 (dt, 1H, *J* = 13.7, 4.8 Hz), 1.95 (t, 1H, *J* = 2.6 Hz), 1.92–0.90 (m, other aliphatic ring protons), 1.34 (s, 3H, CH_3), 1.10 (s, 9H, 3 × CH_3), 0.97, 0.79, 0.77 (s, each 3H, 3 × CH_3), 0.67 (d, 1H, *J* = 11.7 Hz); ^{13}C NMR (100 MHz, $CDCl_3$): δ 200.03, 175.59, 169.18, 128.39, 83.95, 78.64, 68.81, 61.76, 54.89, 48.11, 45.29, 43.50, 43.15, 41.76, 39.11, 39.08, 38.82, 38.54, 37.43, 37.01, 32.69, 31.84, 31.42, 28.78, 28.44, 28.05, 27.21, 26.40, 26.33, 25.68, 23.30, 18.61, 18.00, 17.41, 16.29, 15.53; ESI-HRMS (m/z) Calcd for $C_{36}H_{55}NO_3$ $[M+H]^+$: 550.4255. Found 550.4247.

*5.10 Synthesis of heptakis (2,3-di-O-acetyl-6-deoxy-6-(4-((3β-hydroxyolean-11-oxo-12-en-30-oyl)aminobutyl)-1H-1,2,3-triazol-1-yl))-β-CD (**33**)*

Prepared from **11** and **29** according to general procedure A, the residue was purified by flash chromatography (eluent: CH_2Cl_2:MeOH = 10:1) to afford **33** as a white solid in 46% yield. R_f= 0.15 (eluent: CH_2Cl_2:MeOH = 10:1); ^1H NMR (600 MHz, $CDCl_3$): δ 7.62 (s, 7H), 6.52 (br s, 7H), 5.59 (s, 7H), 5.46 (s, 7H), 5.31 (s, 7H), 4.76 (br s, 27H), 4.47 (s, 7H), 3.56 (s, 7H), 3.32 (s, 7H), 3.16–3.06 (m, 14H), 2.71–2.61 (m, 21H), 2.30 (s, 7H), 2.13–2.11 (m, 7H), 2.11 (s, 21H, 7 × $COCH_3$),

2.01 (m, 7H), 1.99 (s, 21H, 7 × $COCH_3$), 1.98 (m, 7H), 1.85–1.80 (m, 14H), 1.71–1.32 (m, 84H), 1.36 (s, 21H, 7 × CH_3), 1.17 (d, 7H, *J* = 11.3 Hz), 1.10 (s, 63H, 21 × CH_3), 1.01 (m, 7H), 0.98 (s, 21H, 7 × CH_3), 0.87 (m, 7H), 0.78 (s, 42H, 14 × CH_3), 0.65 (br s, 7H); ^{13}C NMR (150 MHz, $CDCl_3$): δ 199.88, 175.81, 170.36, 169.67, 169.37, 147.66, 128.21, 124.07, 96.54, 78.79, 76.92, 70.50, 68.64, 69.82, 61.81, 55.06, 49.91, 48.29, 45.40, 43.43, 43.21, 41.67, 39.55, 39.21, 39.09, 37.52, 37.06, 32.72, 31.87, 31.38, 29.49, 29.29, 28.51, 28.21, 27.19, 26.71, 26.52, 26.37, 25.14, 23.39, 20.74, 20.68, 18.67, 17.46, 16.45, 15.60; MALDI-TOF MS m/z: 5750.2 $[M+H]^+$; 5771.9 $[M+Na]^+$.

*5.11 Synthesis of heptakis (6-deoxy-6-(4-((3β-hydroxyolean-11-oxo-12-en-30-oyl)aminobutyl)-1H-1,2,3-triazol-1-yl))-β-CD (**34**)*

Prepared from **33** according to general procedure B, the residue was purified by RP flash chromatography (eluent: methanol) to afford **34** as a white solid in 95% yield. ^1H NMR (600 MHz, CD_3OD:$CDCl_3$ = 2:1): δ 7.66 (s, 7H), 5.62 (s, 7H), 5.14 (s, 7H), 4.58 (d, 7H, *J* = 12.5 Hz), 4.45 (d, 7H, *J* = 10.6 Hz), 4.16 (d, 7H, *J* = 7.8 Hz), 3.89 (t, 7H, *J* = 7.7 Hz), 3.41 (d, 7H, *J* = 9.1 Hz), 3.24–3.19 (m, 14H), 3.16 (dd, 7H, *J* = 11.6, 4.2 Hz), 3.14–3.11 (m, 7H), 2.71 (d, 7H, *J* = 12.8 Hz), 2.63–2.58 (m, 7H), 2.56–2.51 (m, 7H), 2.38 (s, 7H), 2.13 (br d, 7H, *J* = 14.9 Hz), 2.09 (t, 7H, *J* = 12.2 Hz), 1.94–1.90 (m, 14H), 1.83 (t, 7, *J* = 11.9 Hz), 1.68–1.52 (m, 63H), 1.43–1.36 (m, 35H), 1.39 (s, 21H, 7 × CH_3), 1.21 (d, 7H, *J* = 11.2 Hz), 1.11 (2 × s, 42H, 14 × CH_3), 1.09 (s, 21H, 7 × CH_3), 1.01 (m, 7H), 0.98 (s, 21H, 7 × CH_3), 0.97 (m, 7H), 0.78 (s, 42H, 14 × CH_3), 0.71 (d, 7H, *J* = 11.5 Hz); ^{13}C NMR (150 MHz, CD_3OD:$CDCl_3$ = 2:1): δ 201.93, 177.94, 171.87, 148.34, 128.85, 125.02, 103.12, 83.71, 79.05, 73.65, 73.28, 71.07, 62.76, 55.85, 50.85, 49.57,48.29, 46.36, 44.39, 44.23, 42.20, 40.14, 39.93, 39.81, 38.46, 37.95, 36.31, 33.55, 32.68, 32.60, 31.79, 30.48, 30.37, 30.27, 30.24, 30.09, 29.89, 29.75, 29.28, 28.57, 27.83, 27.47, 27.43, 27.27, 27.17, 25.62, 23.86, 19.31, 18.29, 16.95, 16.22; MALDI-TOF MS m/z: 5182.0 $[M+Na]^+$.

*5.12 Synthesis of heptakis (2,3-di-O-acetyl-6-deoxy-6-(4-((3β-hydroxyolean-11-oxo-12-en-30-oyl)aminophenyl)-1H-1,2,3-triazol-1-yl))-β-CD (**35**)*

Prepared from **11** and **30** according to general procedure A, the residue was purified by flash chromatography (eluent: CH_2Cl_2:MeOH = 15:1) to afford **35** as a white solid in 70% yield. R_f = 0.30 (eluent: CH_2Cl_2:MeOH = 10:1); ^1H NMR (600 MHz, $CDCl_3$): δ 8.01–7.66 (m, 28H), 7.48 (s, 7H), 5.65 (s, 7H), 5.48 (s, 7H), 5.35 (m, 7H), 4.81–4.59 (m, 28H), 3.66 (m, 7H), 3.22 (d, 7H, *J* = 5.0 Hz), 2.78 (s, 7H), 2.31 (s, 7H), 2.22 (m, 7H), 2.04 (m, 7H), 2.07 (s, 21H, 7 × $COCH_3$), 2.01 (m, 7H),

2.00 (s, 21H, 7 × CH_3), 1.98 (m, 7H), 1.81 (m, 7H), 1.73–1.32 (m, 70H), 1.36, 1.22 (s, each 27H, 14 × CH_3), 1.17 (m, 7H), 1.10 (s, 42H, 21 × CH_3), 1.02 (m, 7H), 1.00 (s, 42H, 14 × CH_3), 0.96 (m, 7H), 0.79 (s, 42H, 21 × CH_3), 0.69 (d, 7H, J = 7.9 Hz); ^{13}C NMR (150 MHz, $CDCl_3$): δ 200.04, 174.24, 170.32, 169.42, 147.16, 138.03, 128.38, 126.17, 122.40, 120.64, 96.73, 78.72, 77.33, 70.56, 69.78, 69.57, 61.84, 54.94, 50.22, 48.06, 45.37, 44.52, 43.22, 41.62, 39.14, 39.11, 37.43, 37.05, 32.74, 31.94, 31.69, 29.09, 28.48, 28.10, 27.28, 26.42, 23.41, 20.73, 18.67, 17.46, 16.32, 15.60; MALDI-TOF MS m/z: 5889.3 $[M+H]^+$.

*5.13 Synthesis of heptakis (6-deoxy-6-(4-((3β-hydroxyolean-11-oxo-12-en-30-oyl)aminophenyl)-1H-1,2,3-triazol-1-yl))-β-CD (**36**)*

Prepared from **35** according to general procedure B, the residue was purified by RP flash chromatography (eluent: methanol) to afford **36** as a white solid in 83% yield. ^{1}H NMR (600 MHz, DMSO-d_6): δ 9.15 (s, 7H), 8.08 (s, 7H), 7.50 (s, 28H), 5.43 (s, 7H), 5.13 (s, 7H), 4.52 (s, 7H), 4.31 (s, 7H), 4.13 (s, 14H), 3.75 (s, 7H), 3.40 (s, 7H), 3.34 (s, 7H), 3.02 (s, 7H), 2.59 (s, 7H), 2.30 (s, 7H), 2.10–2.05 (m, 21H), 1.94 (s, 7H), 1.71 (s, 7H), 1.611.27 (m, 56H), 1.32 (s, each 21H, 7 × CH_3), 1.12 (m, 7H), 1.10, 1.02, 1.00 (s, each 21H, 21 × CH_3), 0.93 (m, 14H), 0.90 (s, 21H, 7 × CH_3), 0.69, 0.68 (s, each 21H, 14 × CH_3), 0.67 (m, 7H); ^{13}C NMR (150 MHz, DMSO-d_6): δ 198.78, 174.13, 169.38, 145.88, 138.58, 127.49, 125.53, 125.16, 122.46, 120.58, 101.83, 82.92, 76.58, 72.45, 71.83, 69.52, 61.14, 54.11, 49.41, 47.61, 44.75, 43.84, 42.82, 40.67, 38.74, 38.50, 37.24, 36.62, 32.09, 31.39, 30.37, 28.33, 28.11, 26.93, 25.98, 25.85, 23.01, 18.28, 17.12, 16.12, 15.94; MALDI-TOF MS m/z: 5298.1 $[M+H]^+$.

*5.14 Synthesis of heptakis (2,3-di-O-acetyl-6-deoxy-6-(4-(((3β-hydroxyolean-11-oxo-12-en-30-oyl) piperazine)ethanamine)-1H-1,2,3-triazol-1-yl))-β-CD (**37**)*

Prepared from **11** and **32** according to general procedure A, the residue was purified by flash chromatography (eluent: CH_2Cl_2:MeOH = 2:1) to afford **37** as a white foam solid in 30% yield. R_f = 0.15 (eluent: CH_2Cl_2:MeOH = 3:1); ^{1}H NMR (600 MHz, $CDCl_3$): δ 7.72 (s, 7H), 6.58 (br s, 7H), 5.68 (s, 7H), 5.51 (s, 7H), 5.35 (t, 7H, J = 8.5 Hz), 4.92 (d, 7H, J = 12.5 Hz), 4.79 (d, 7H, J = 11.7 Hz), 4.70 (dd, 7H, J = 9.5, 2.6 Hz), 4.48 (d, 7H, J = 5.0 Hz), 3.54–3.47 (m, 21H), 3.40–3.26 (m, 14H), 3.19 (dd, 7H, J = 11.2, 4.4 Hz), 2.74 (td, 7H, J = 13.2, 3.2 Hz), 2.47 (s, 70H), 2.15 (dd, 7H, J = 13.5, 3.5 Hz), 2.04 (s, 21H, 7 × $COCH_3$), 2.01–2.00 (m, 14H), 1.99 (s, 21H, 7 × $COCH_3$), 1.98–1.96 (m, 14H), 1.84–1.41 (m, 77H), 1.37 (s, 21H, 7 × CH_3), 1.33–1.18 (m, 21H), 1.12, 1.11, 1.10

(each s, each 21H, 21 × CH_3), 1.02 (d, 7H, J = 11.0 Hz), 0.99 (s, 21H, 7 × CH_3), 0.93–0.90 (m, 7H), 0.79 (s, 42H, 14 × CH_3), 0.67 (d, 7H, J = 11.6 Hz); ^{13}C NMR (150 MHz, $CDCl_3$): δ 199.87, 175.86, 170.37, 144.75, 128.36, 125.62, 96.32, 78.64, 77.20, 70.60, 69.73, 61.83, 56.67, 55.03, 53.22, 53.08, 52.63, 49.94, 48.14, 45.42, 43.56, 43.19, 41.73, 39.39, 39.15, 37.50, 37.05, 35.94, 32.74, 31.88, 31.42, 29.59, 28.60, 28.18, 27.36, 26.49, 26.39, 23.42, 20.75, 20.70, 18.67, 17.46, 16.46, 15.71; MALDI-TOF MS m/z: 6239.4 $[M+H]^+$; 6261.9 $[M+Na]^+$.

*5.15 Synthesis of heptakis (6-deoxy-6-(4-(((3β-hydroxyolean-11-oxo-12-en-30-oyl) piperazine)ethanamine)-1H-1,2,3-triazol-1-yl))-β-CD (**38**)*

Prepared from **37** according to general procedure B, the residue was purified by RP flash chromatography (eluent: methanol) to afford **38** as a white solid in 87% yield. ^{1}H NMR (600 MHz, CD_3OD:$CDCl_3$ = 2:1): δ 7.89 (s, 7H), 5.66 (s, 7H), 5.14 (s, 7H), 4.63 (d, 7H, J = 13.3 Hz), 4.52 (d, 7H, J = 10.9 Hz), 4.20 (d, 7H, J = 7.4 Hz), 3.89 (t, 7H, J = 8.9 Hz), 3.61–3.37 (m, 28H), 3.28–3.22 (m, 14H), 3.17 (dd, 7H, J = 10.1, 3.7 Hz), 2.71 (br d, 7H, J = 12.6 Hz), 2.55 (br s, 28H), 2.49 (s, 28H), 2.40 (s, 7H), 2.11–1.44 (m, 98H), 1.40 (s, 21H, 7 × CH_3), 1.37–1.21 (m, 49H), 1.12 (s, 42H, 14 × CH_3), 1.10 (s, 21H, 7 × CH_3), 1.03 (d, 7H, J = 13.4 Hz), 0.97 (s, 21H, 7 × CH_3), 0.81 (s, 21H, 7 × CH_3), 0.78 (s, 21H, 7 × CH_3), 0.72 (d, 7H, J = 11.6 Hz); ^{13}C NMR (150 MHz, CD_3OD:$CDCl_3$ = 2:1): δ 201.96, 178.10, 171.85, 144.11, 128.99, 127.38, 103.16, 83.60, 73.71, 73.30, 71.07, 62.84, 57.90, 55.91, 54.38, 53.42, 51.00, 47.61, 46.44, 44.51, 44.26, 42.26, 40.17, 39.99, 38.51, 38.02, 37.11, 33.59, 32.74, 32.69, 31.86, 29.71, 29.38, 28.60, 27.57, 27.32, 27.22, 23.92, 19.34, 18.35, 17.00, 16.27; MALDI-TOF MS m/z: 5650.2 $[M+H]^+$; 5672.9 $[M+Na]^+$.

6. Selected NMR and MALDI-TOF MS spectra of conjugates **21-28** and **33-38**

^{1}H NMR of compound **21**

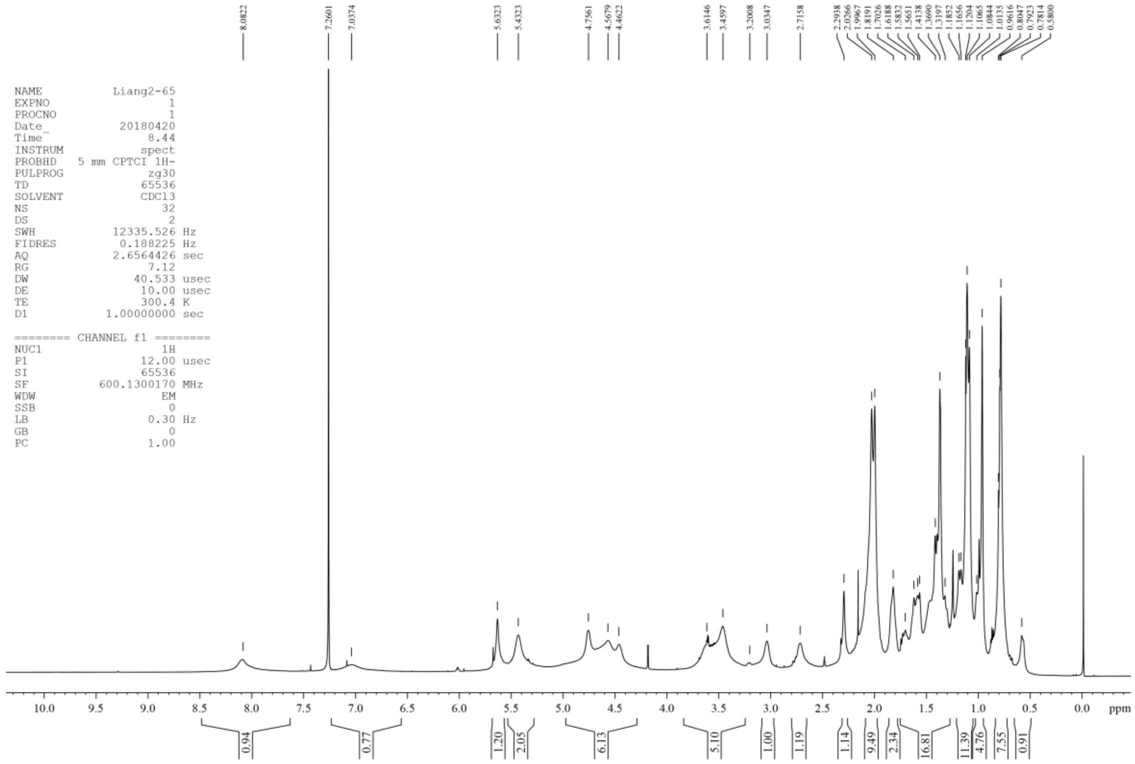

^{13}C NMR of compound **21**

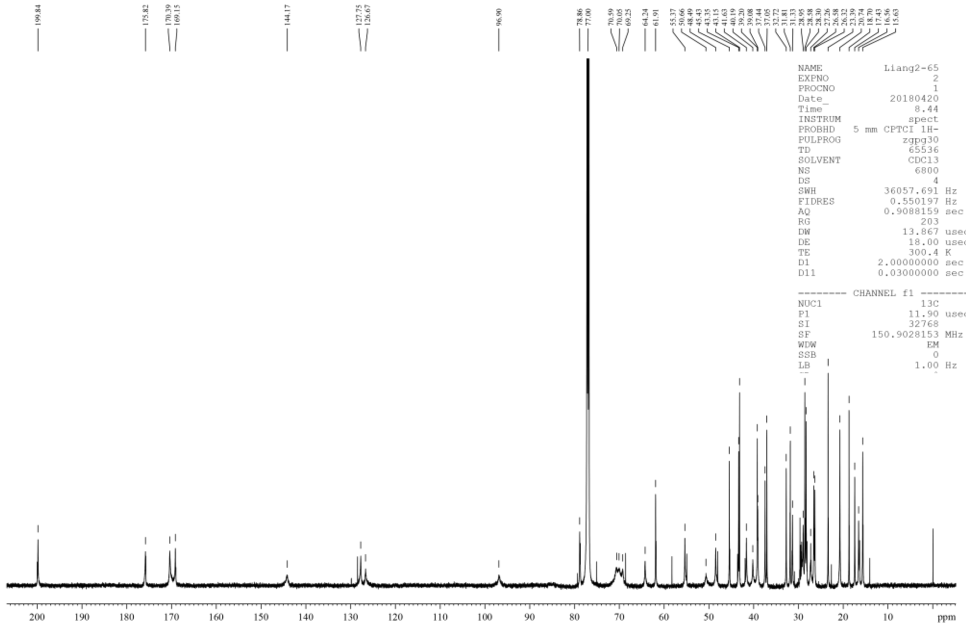

HSQC of compound **21**

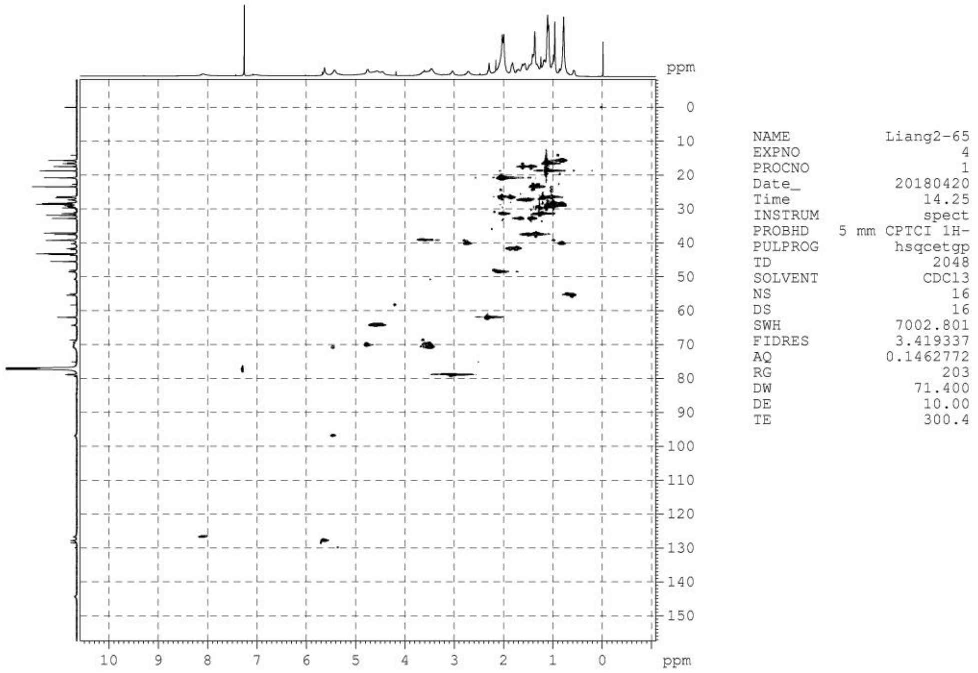

MALDI-TOF of compound **21**

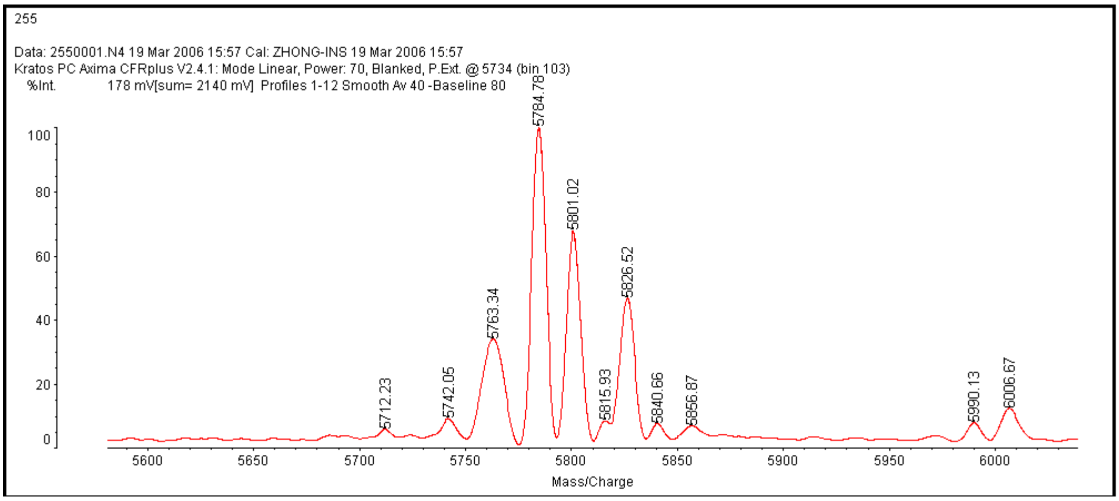

^{1}H NMR of compound **22**

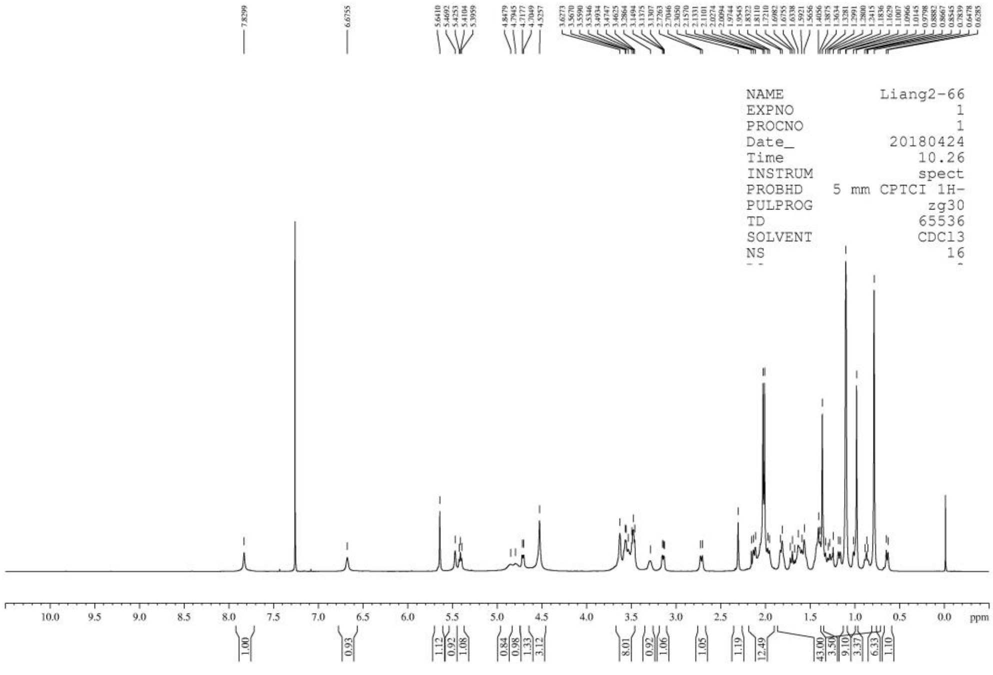

^{13}C NMR of compound **22**

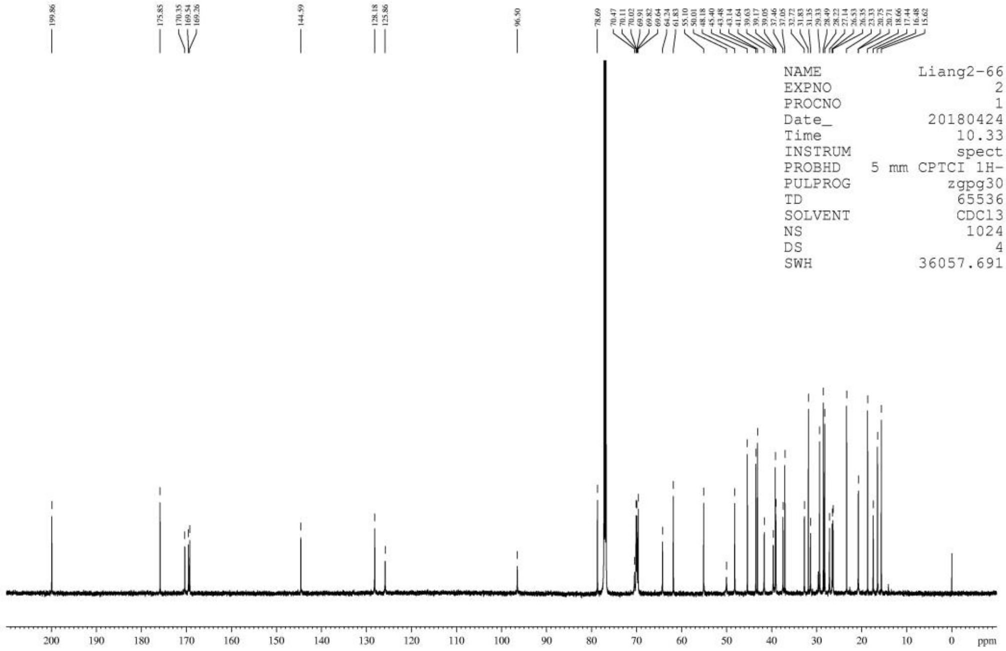

HSQC of compound **22**

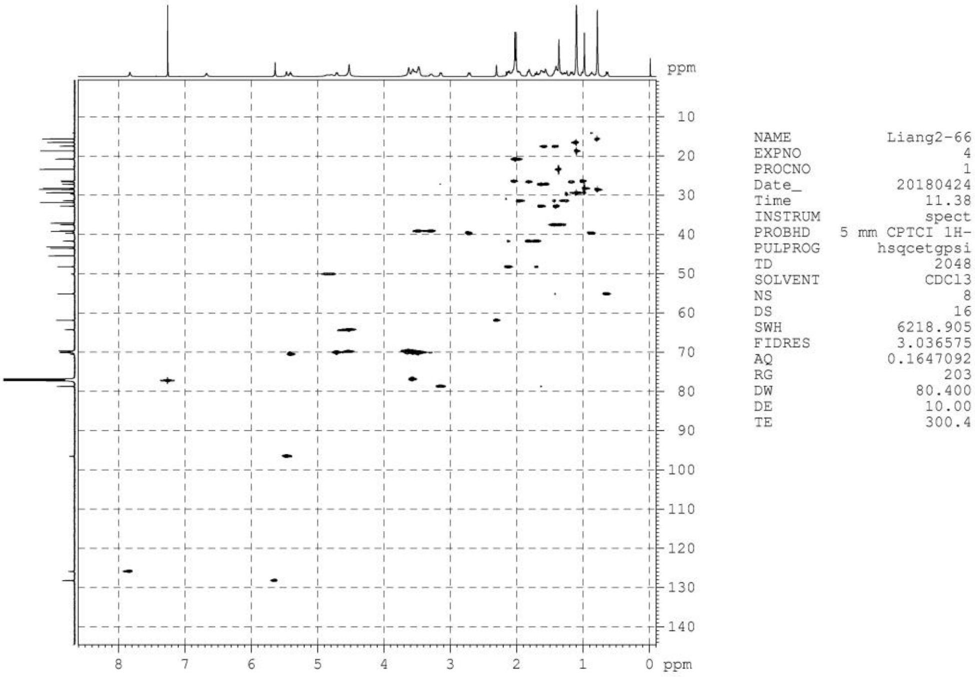

MALDI-TOF of compound **22**

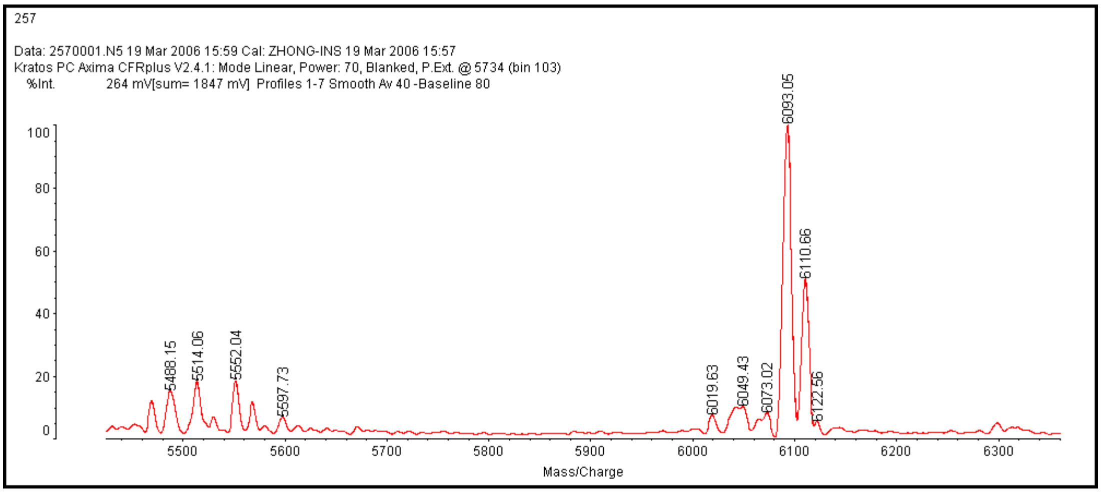

^{1}H NMR of compound **23**

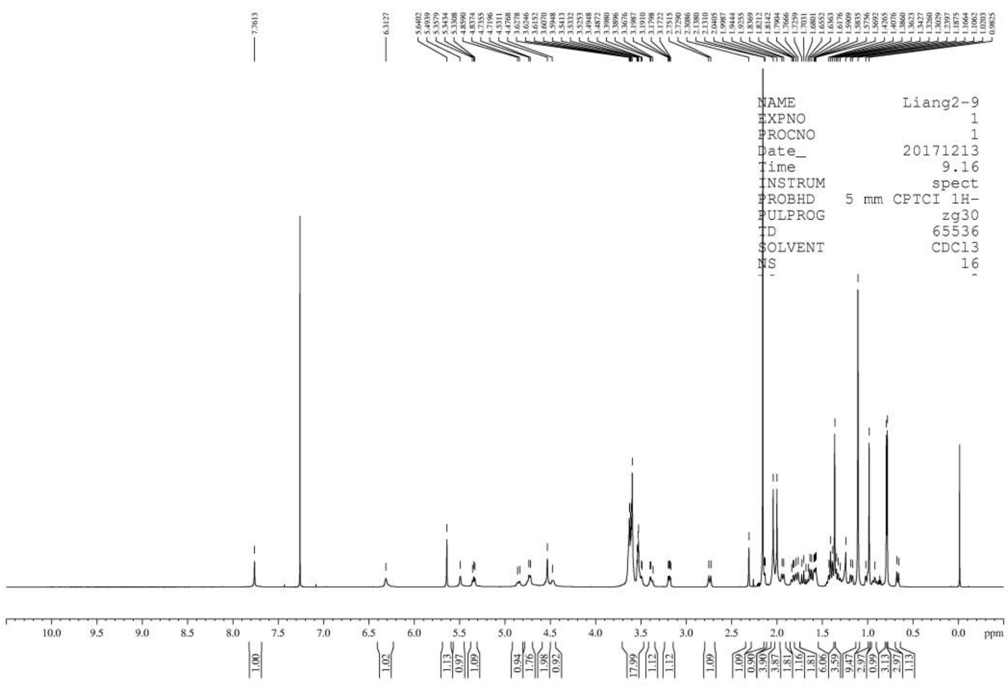

^{13}C NMR of compound **23**

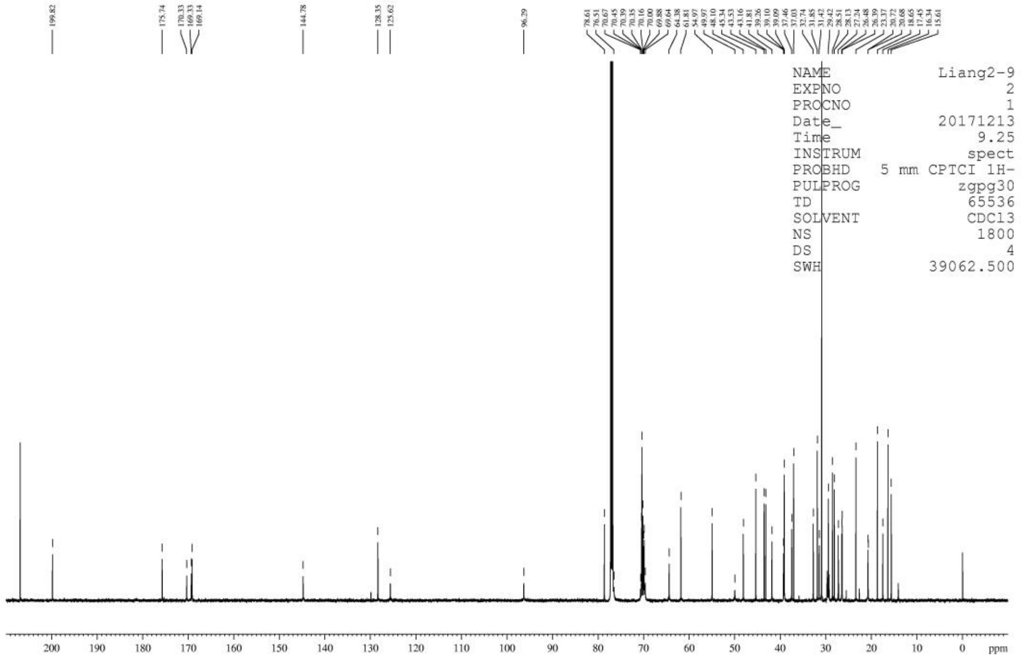

HSQC of compound **23**

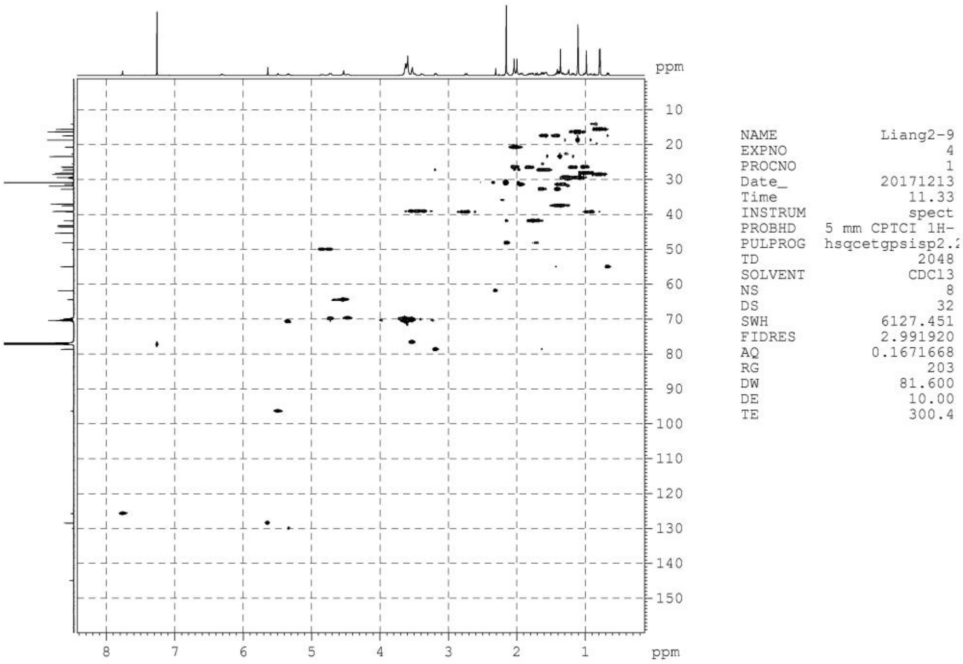

MALDI-TOF of compound **23**

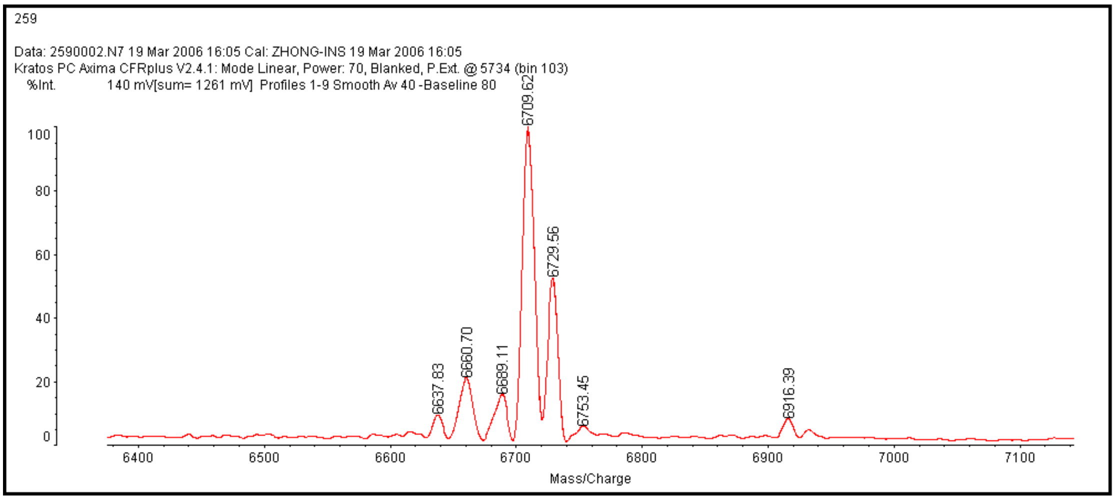

^{1}H NMR of compound **24**

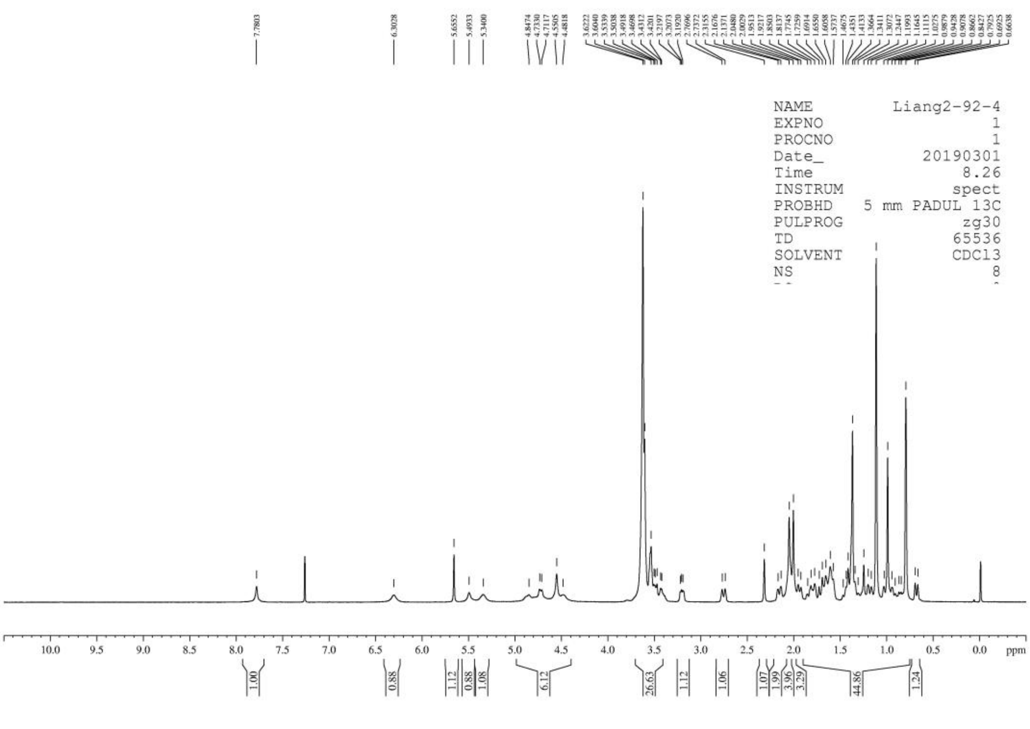

^{13}C NMR of compound **24**

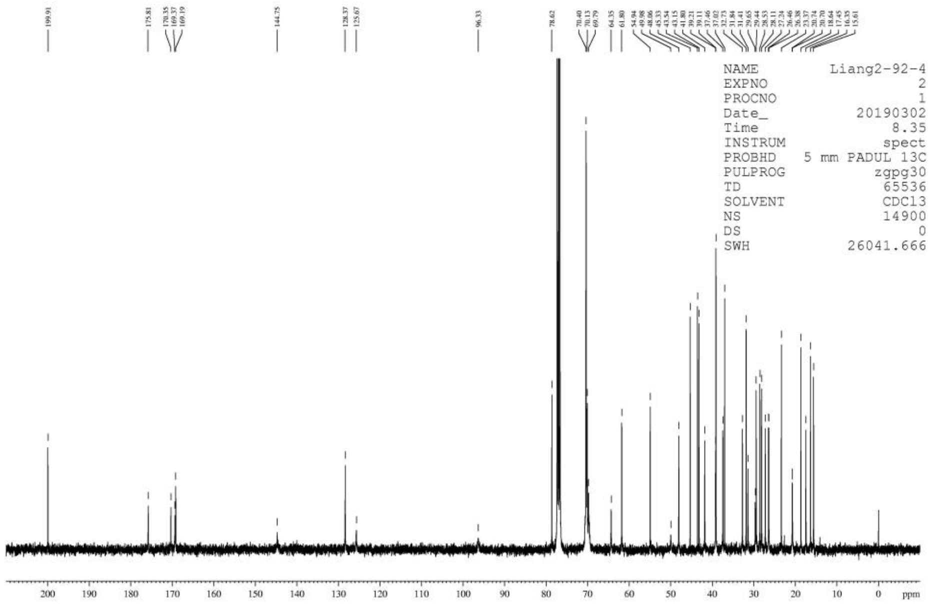

HSQC of compound **24**

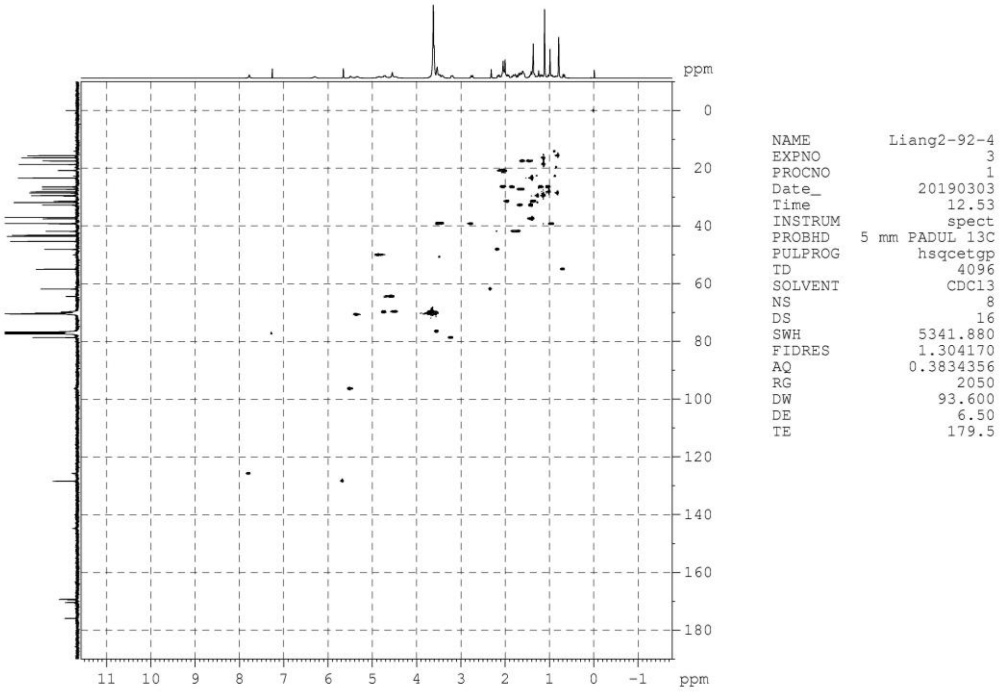

MALDI-TOF of compound **24**

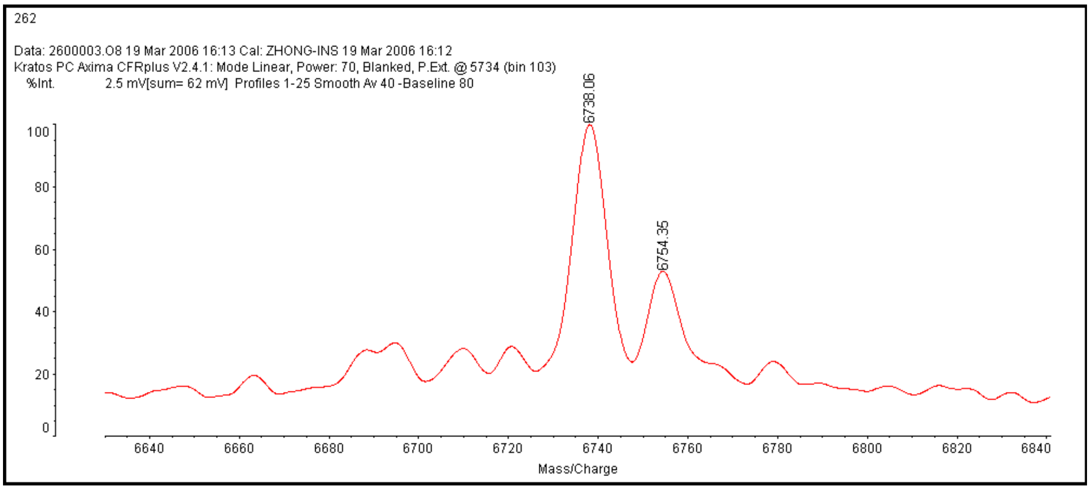

^{1}H NMR of compound **25**

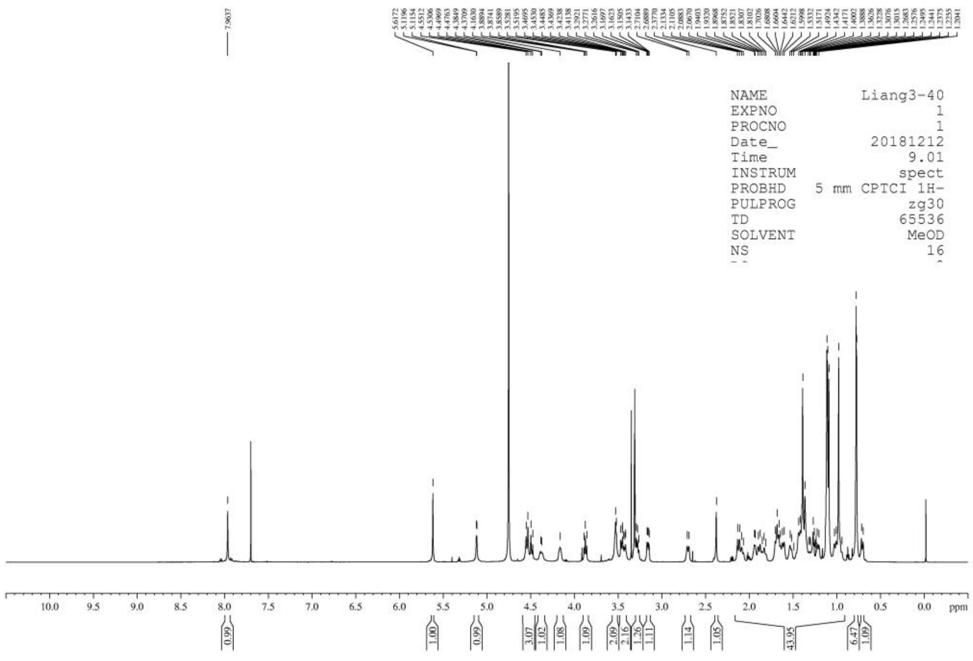

^{13}C NMR of compound **25**

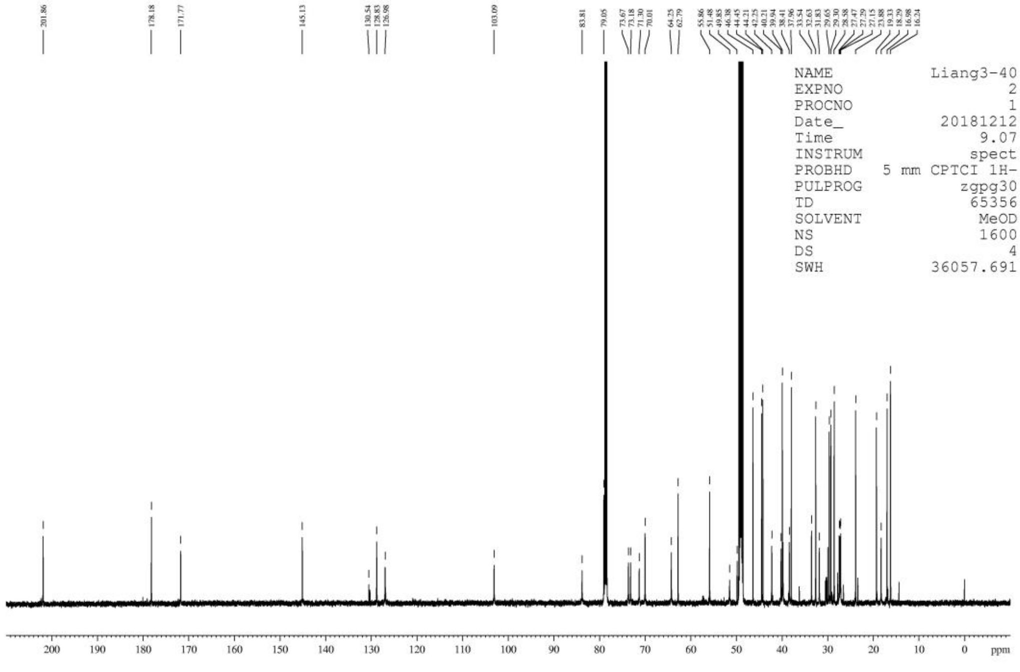

HSQC of compound **25**

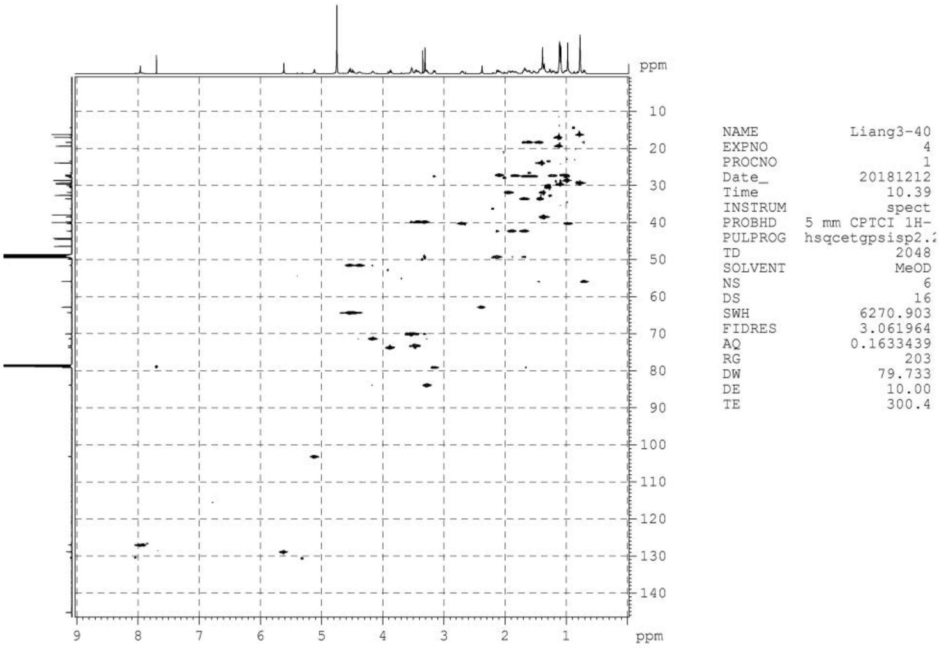

MALDI-TOF of compound **25**

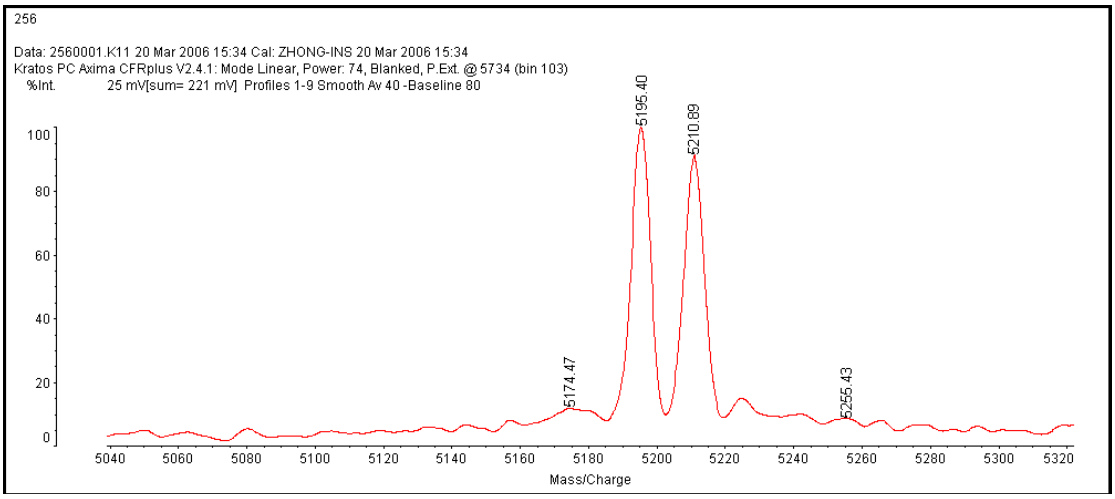

^{1}H NMR of compound **26**

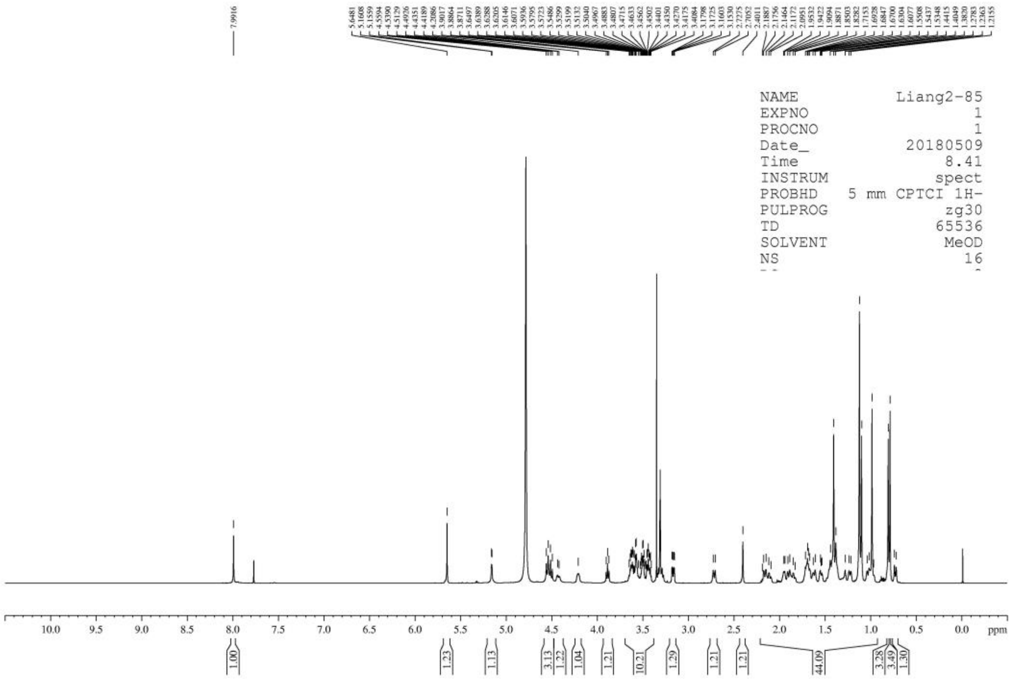

^{13}C NMR of compound **26**

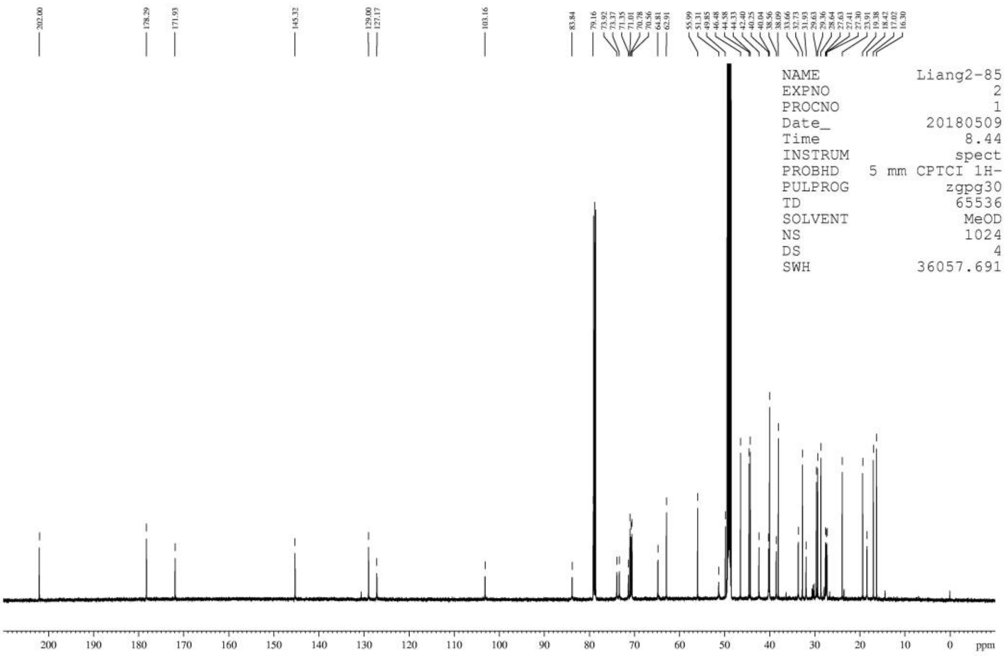

HSQC of compound **26**

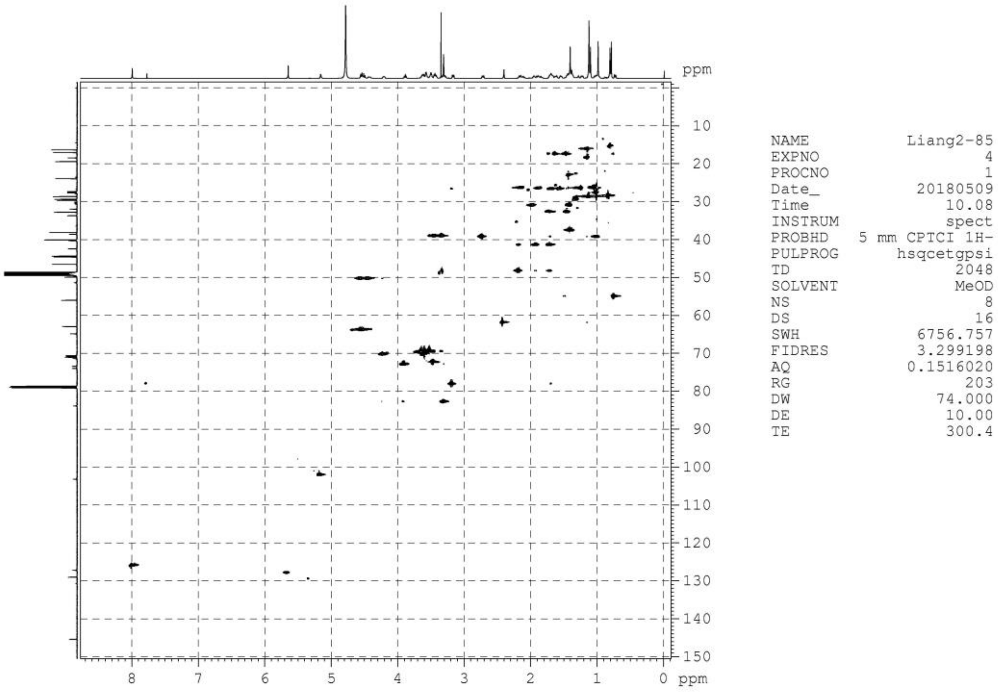

MALDI-TOF of compound **26**

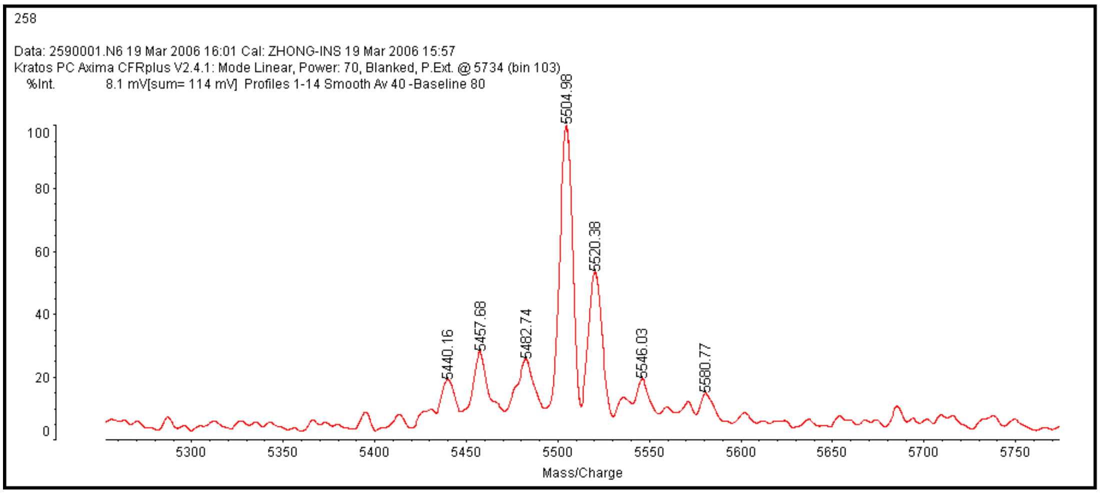

^{1}H NMR of compound **27**

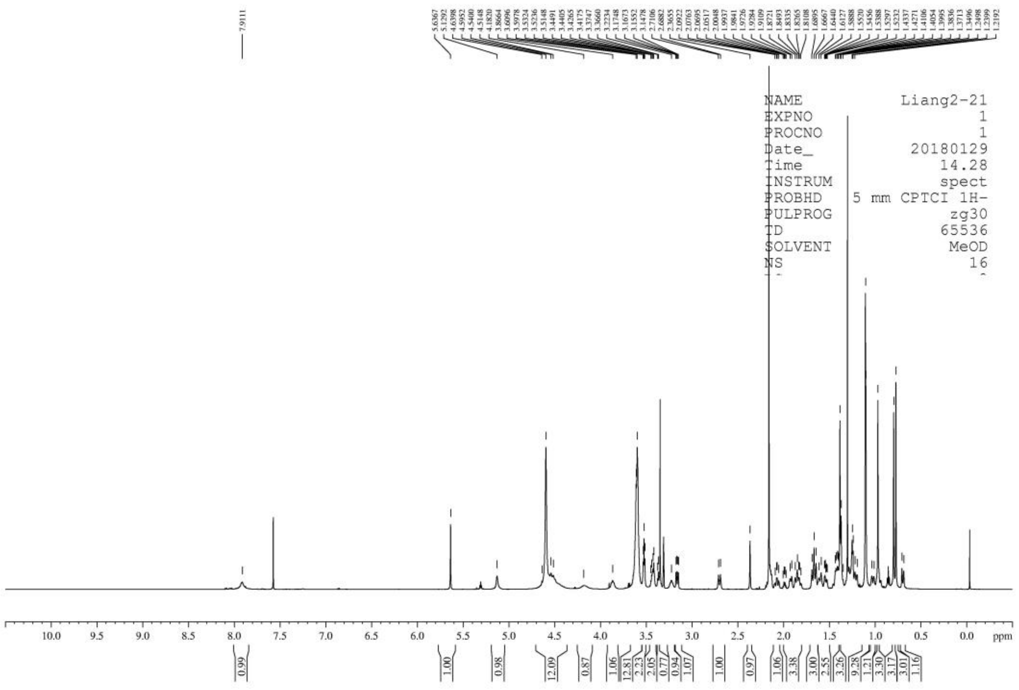

^{13}C NMR of compound **27**

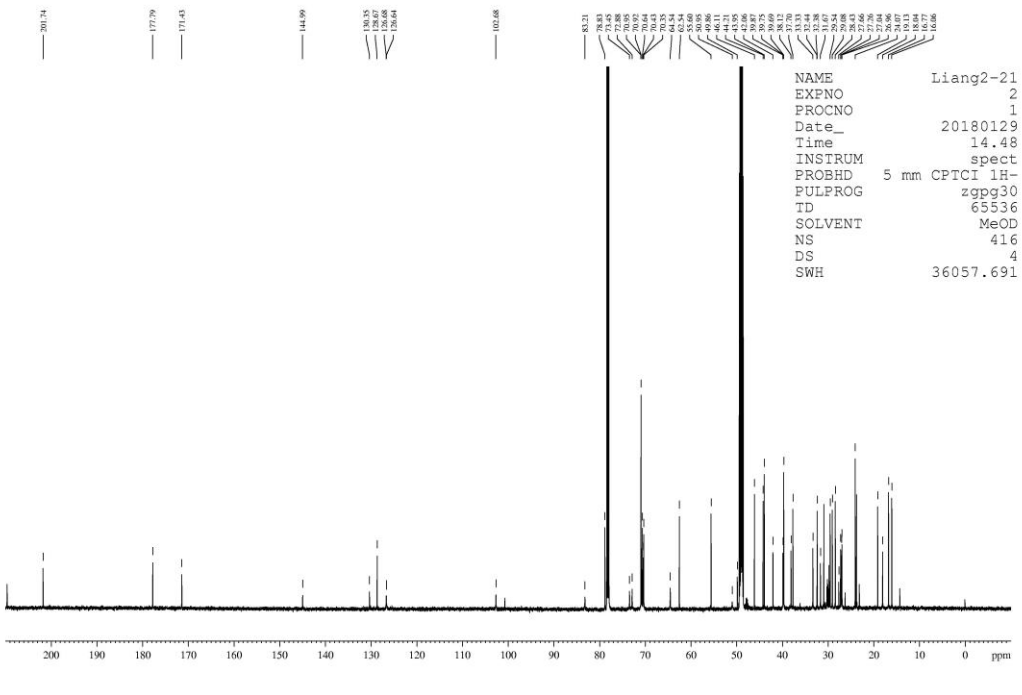

HSQC of compound **27**

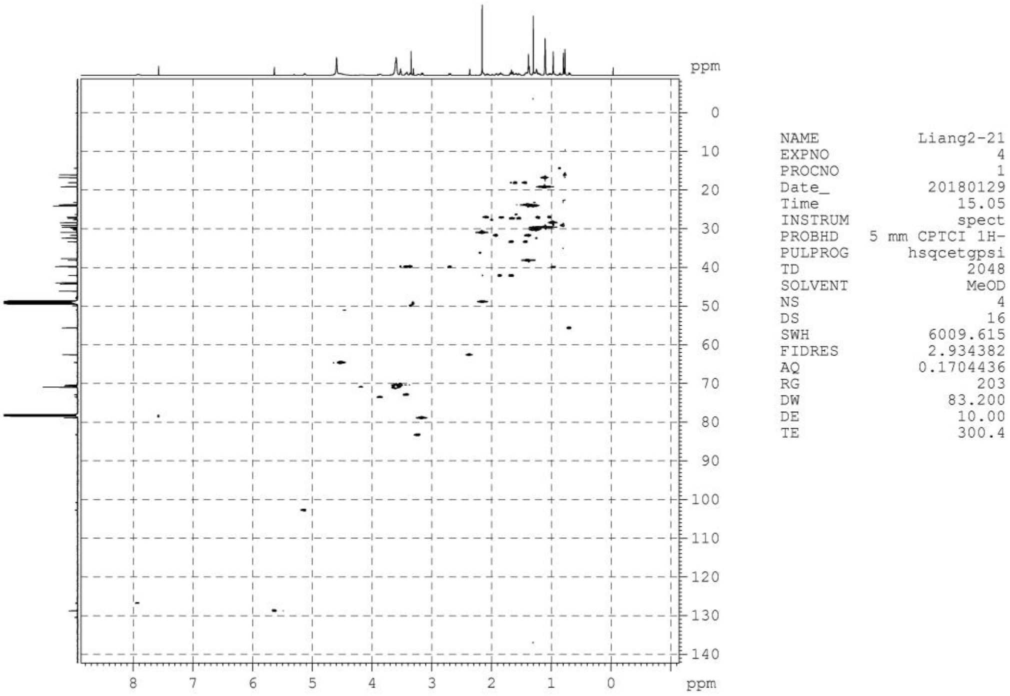

MALDI-TOF of compound **27**

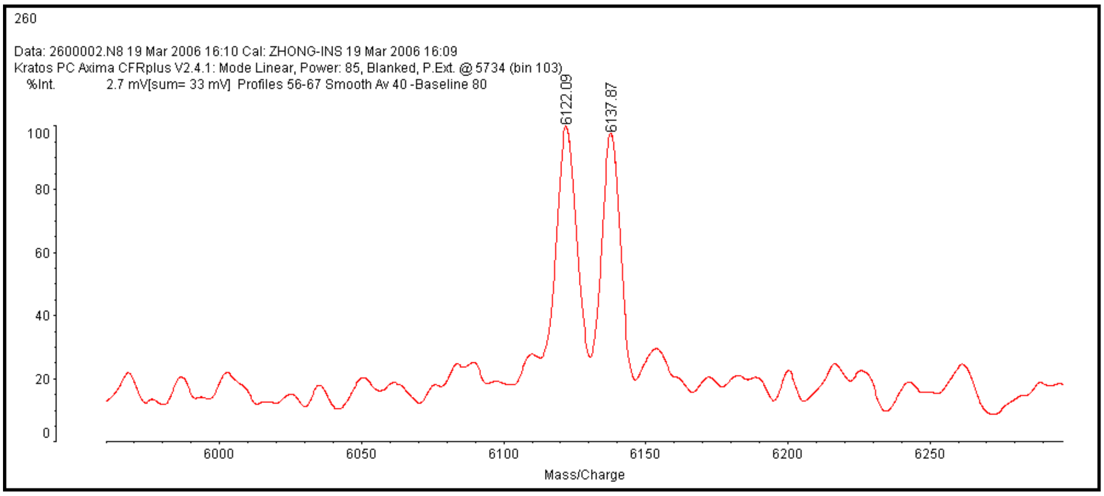

^{1}H NMR of compound **28**

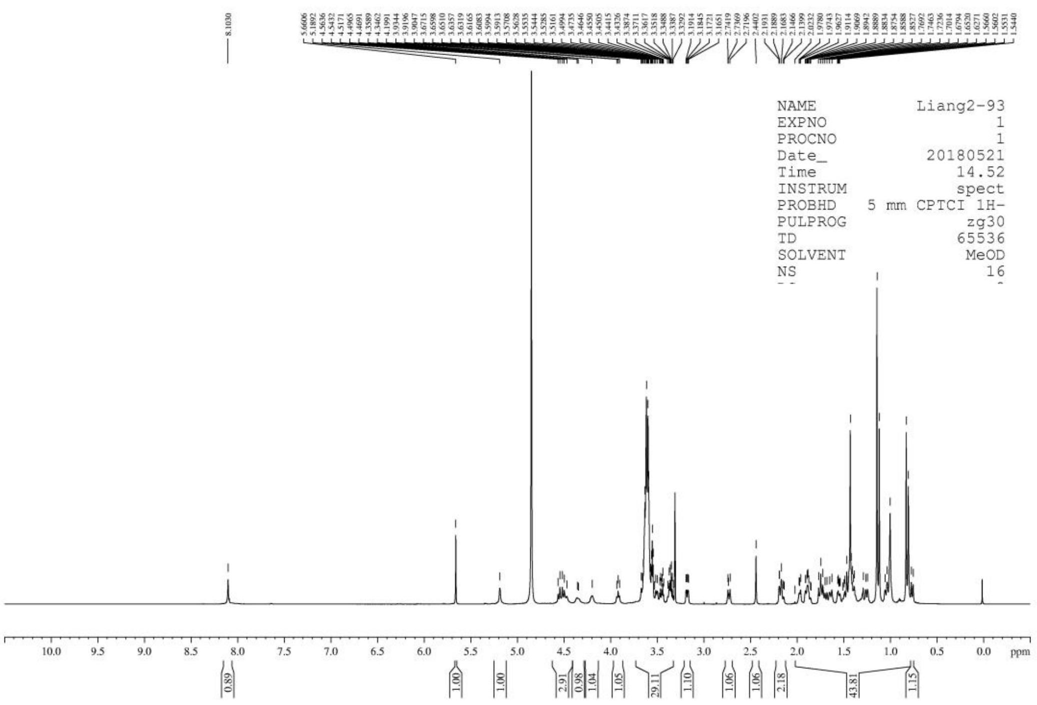

^{13}C NMR of compound **28**

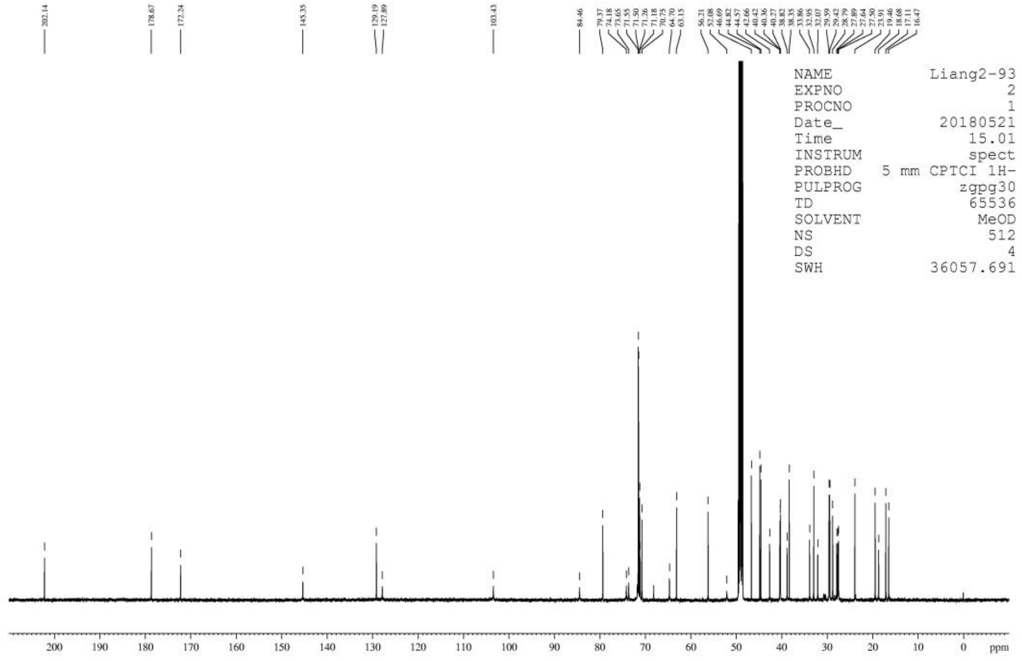

HSQC of compound **28**

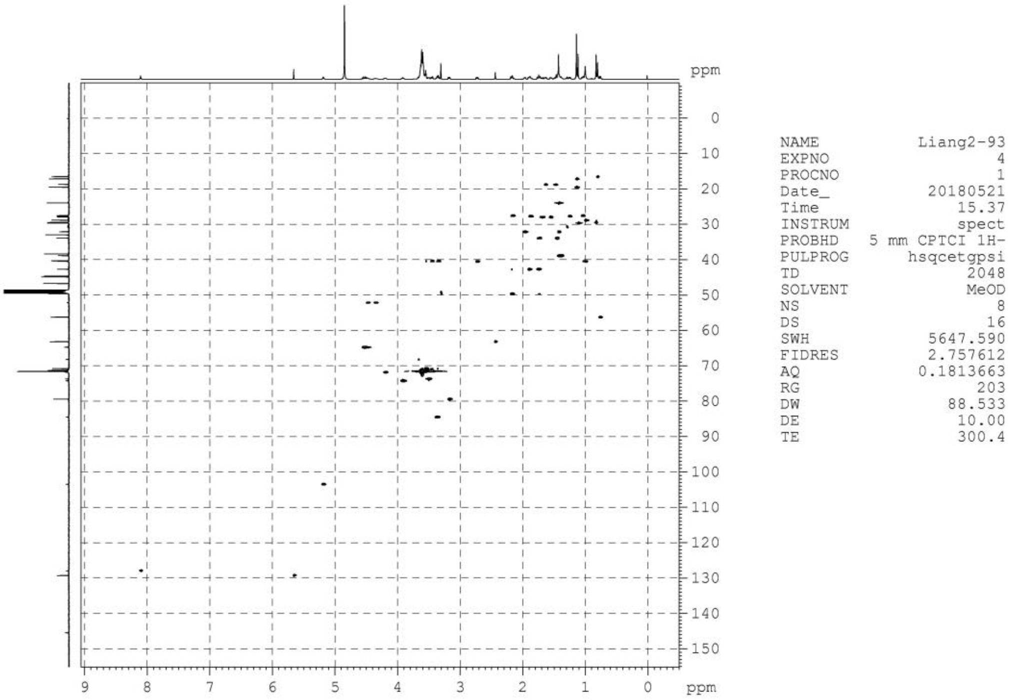

MALDI-TOF of compound **28**

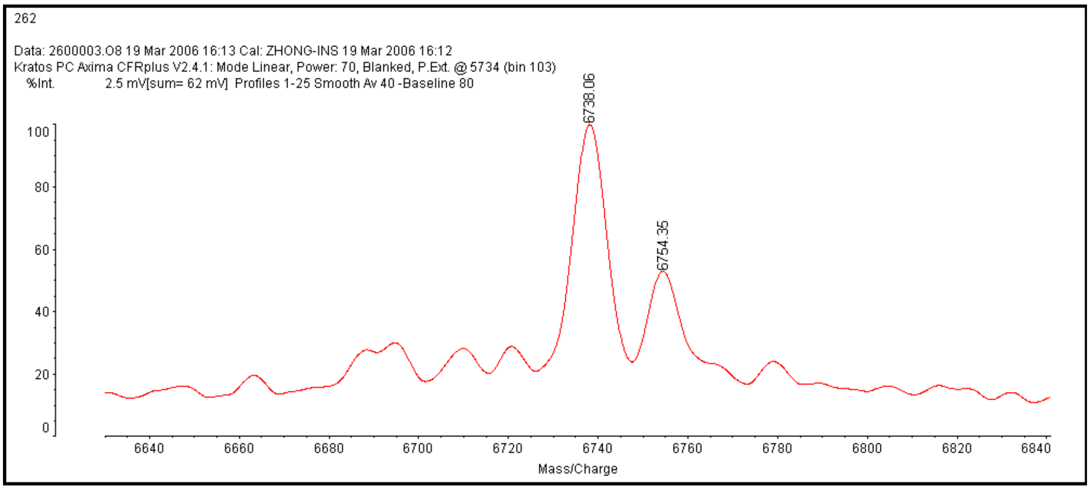

^{1}H NMR of compound **33**

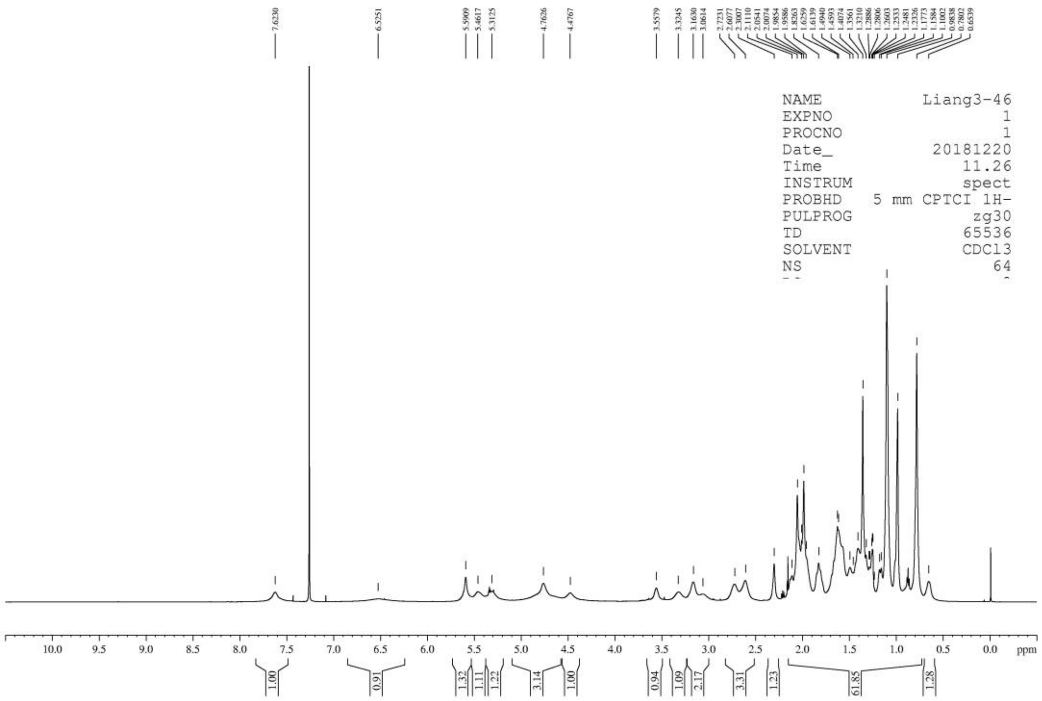

^{13}C NMR of compound **33**

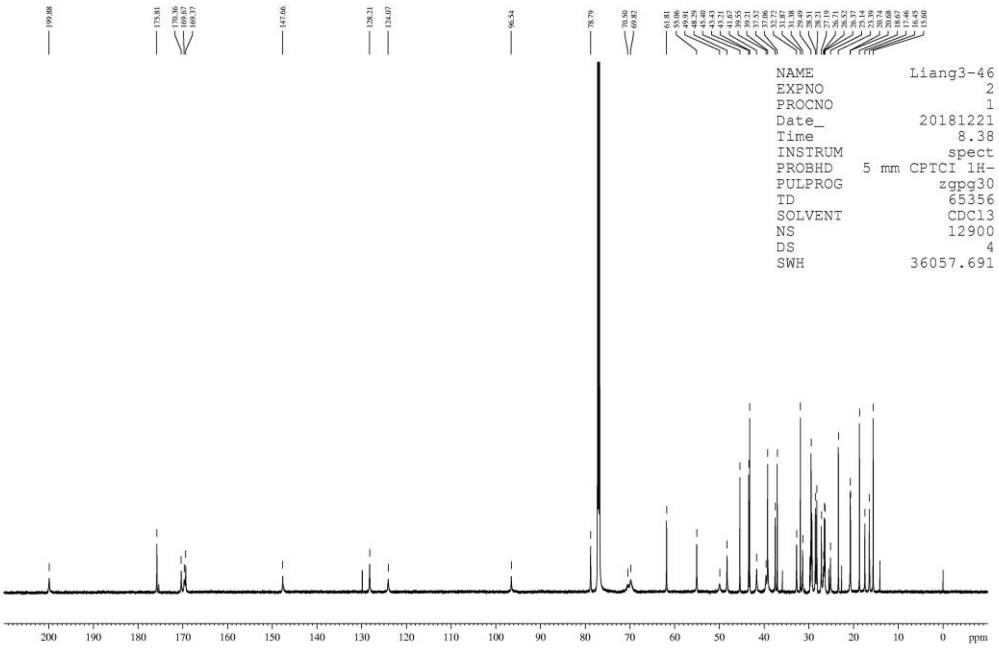

HSQC of compound **33**

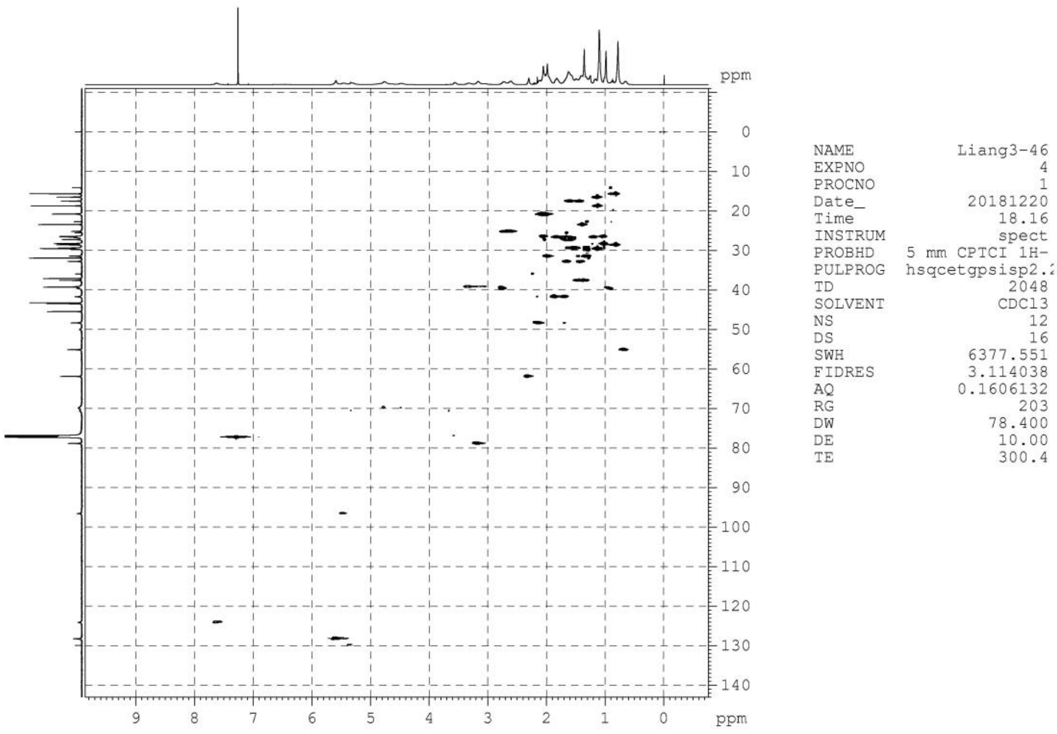

MALDI-TOF of compound **33**

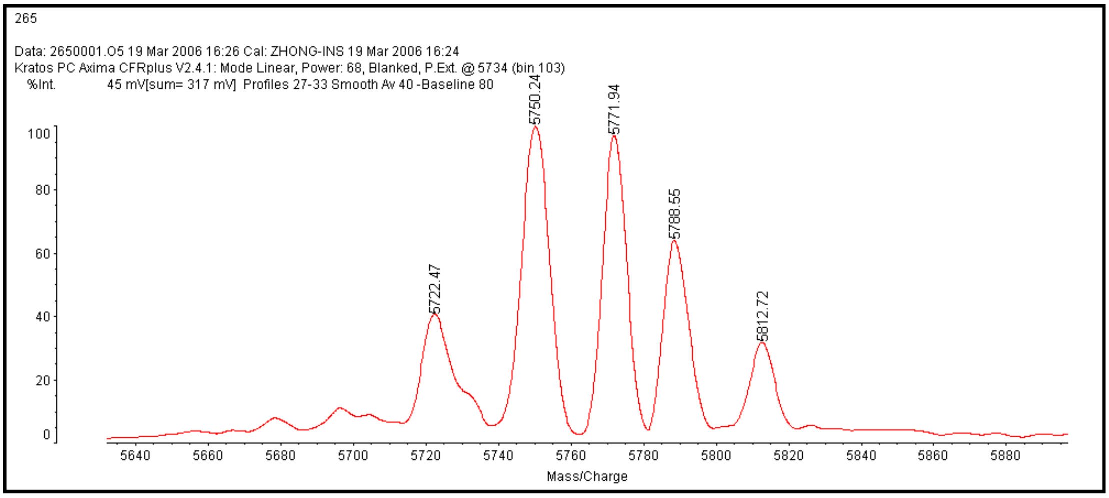

^{1}H NMR of compound **34**

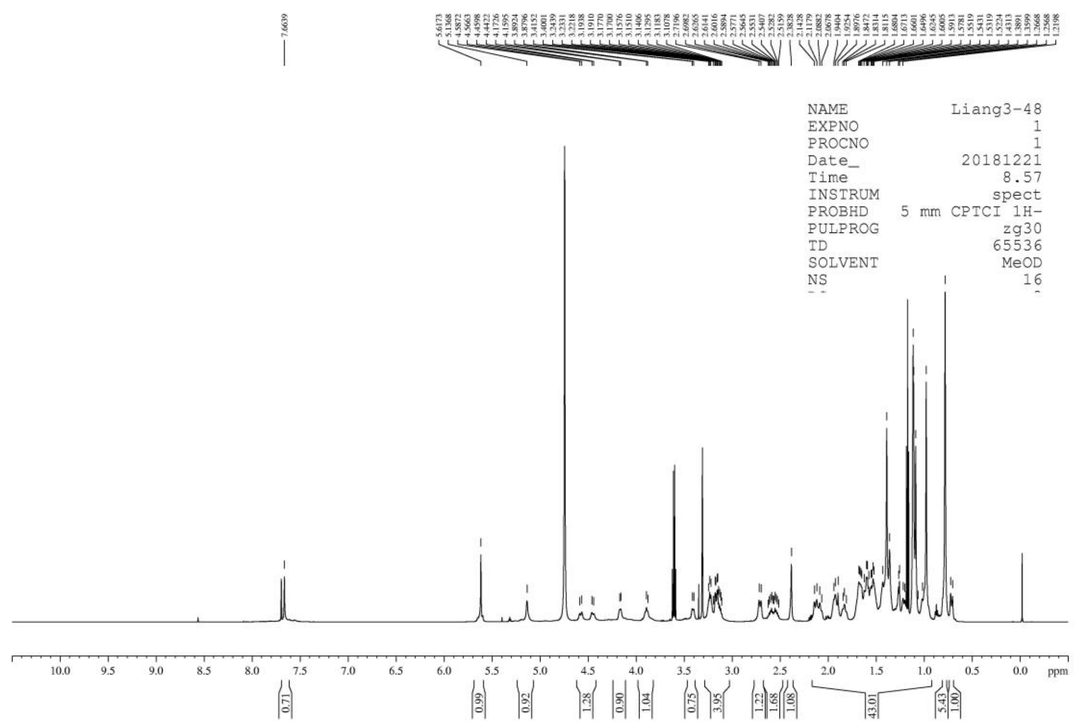

^{13}C NMR of compound **34**

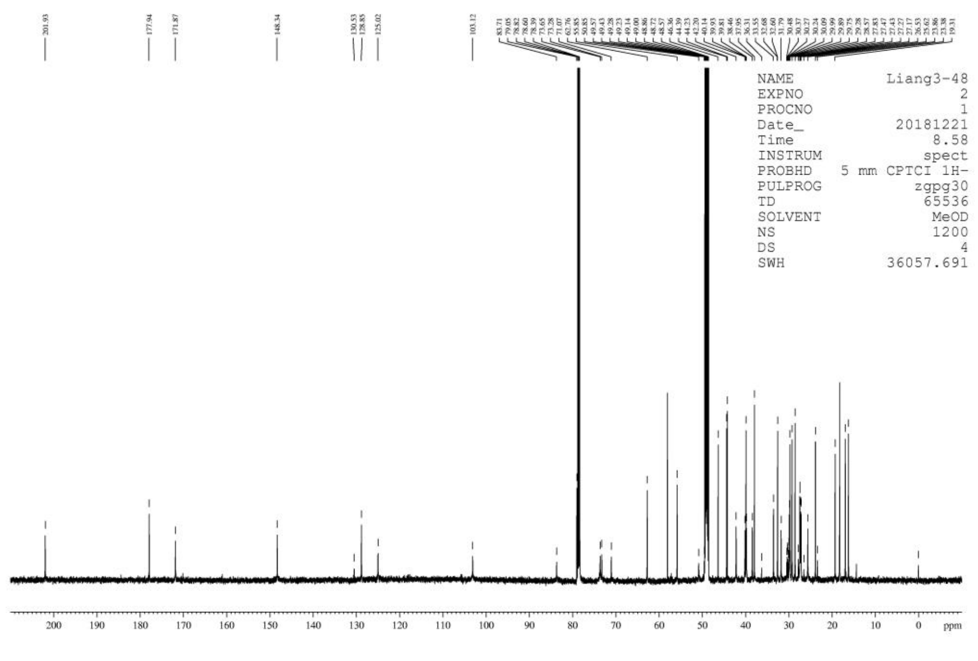

HSQC of compound **34**

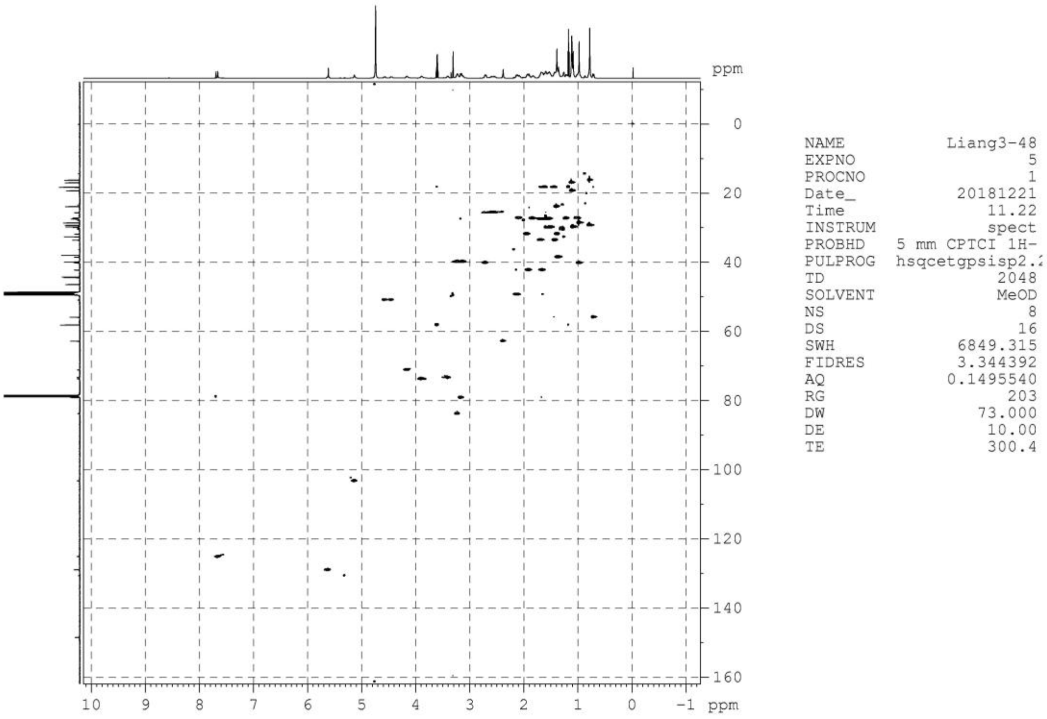

MALDI-TOF of compound **34**

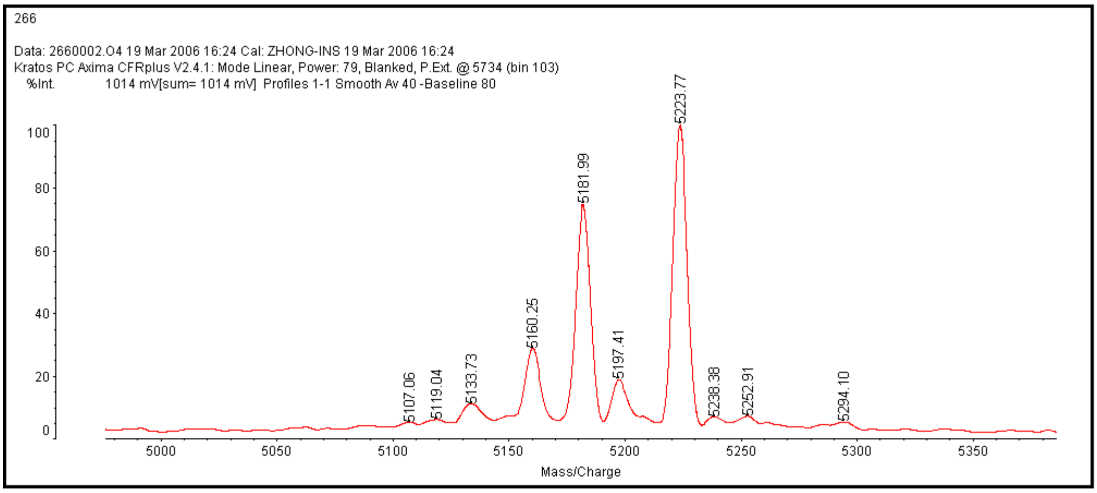

^{1}H NMR of compound **35**

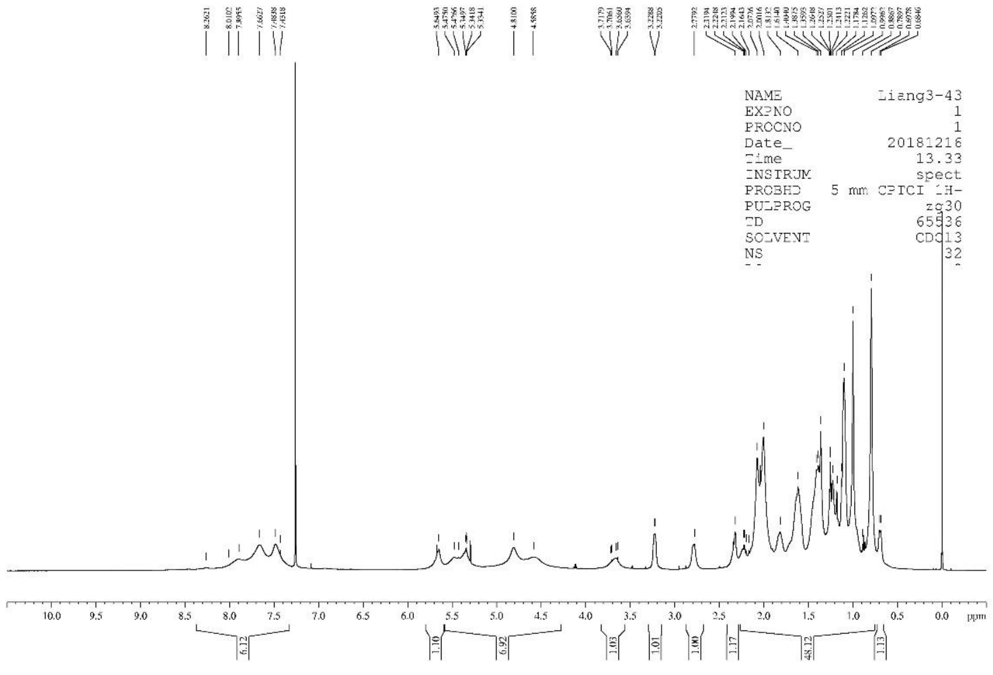

^{13}C NMR of compound **35**

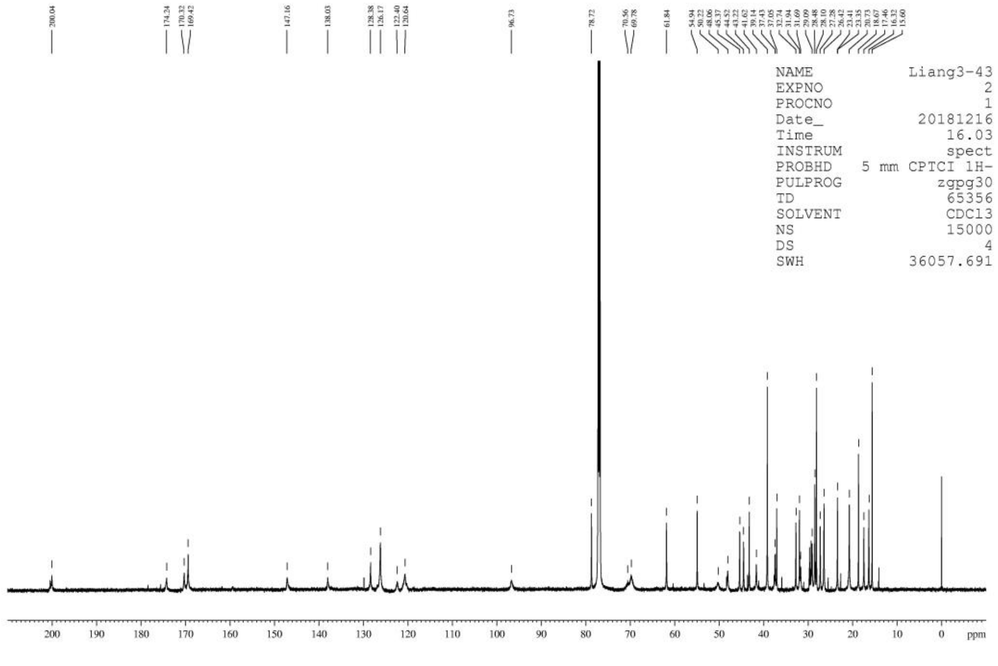

HSQC of compound **35**

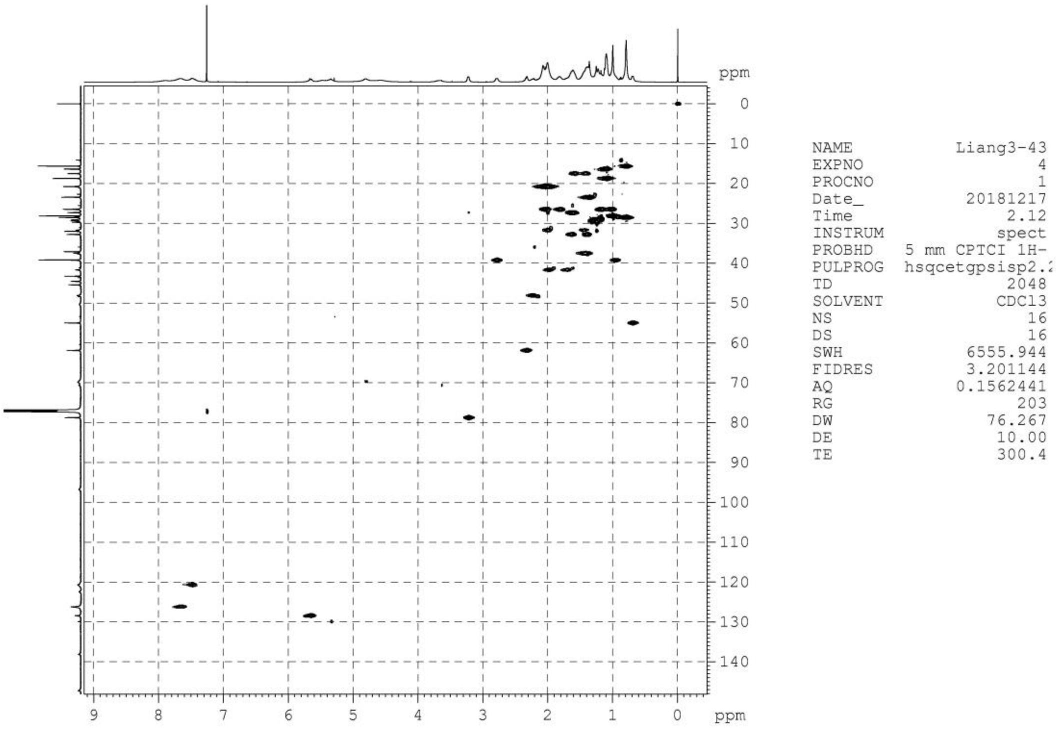

MALDI-TOF of compound **35**

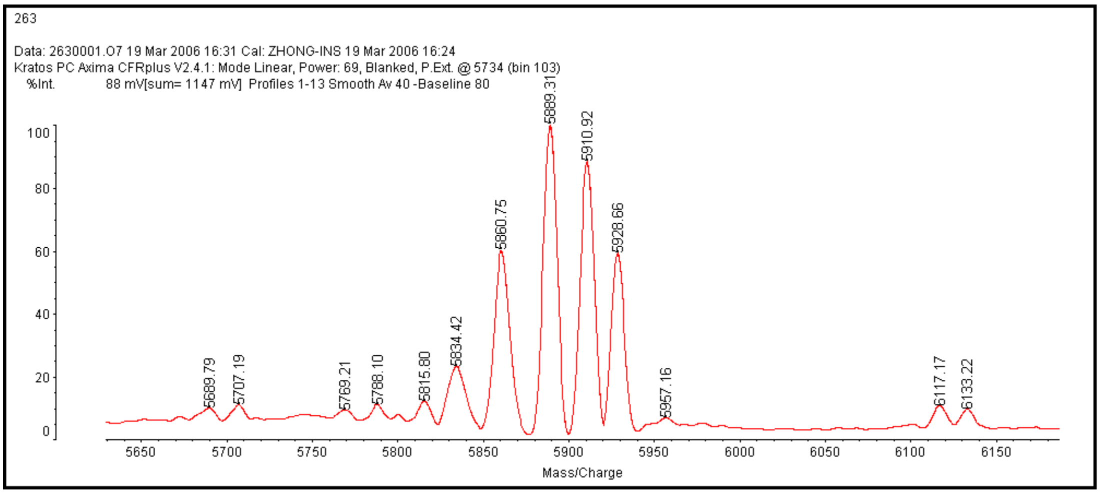

^{1}H NMR of compound **36**

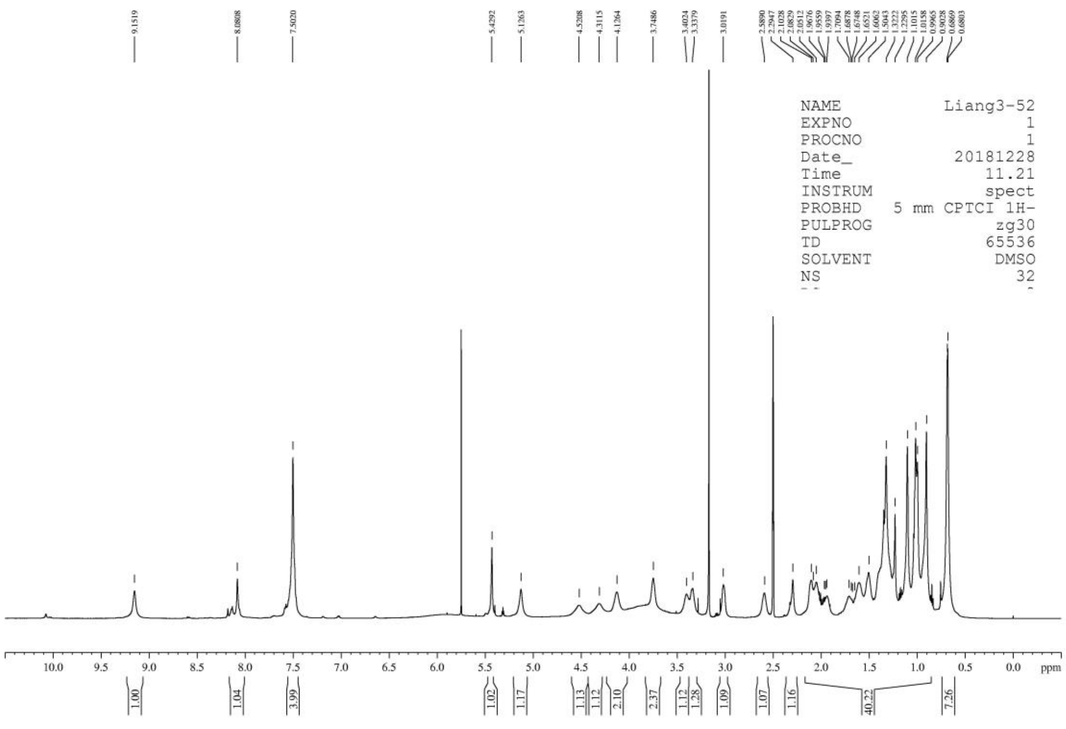

^{13}C NMR of compound **36**

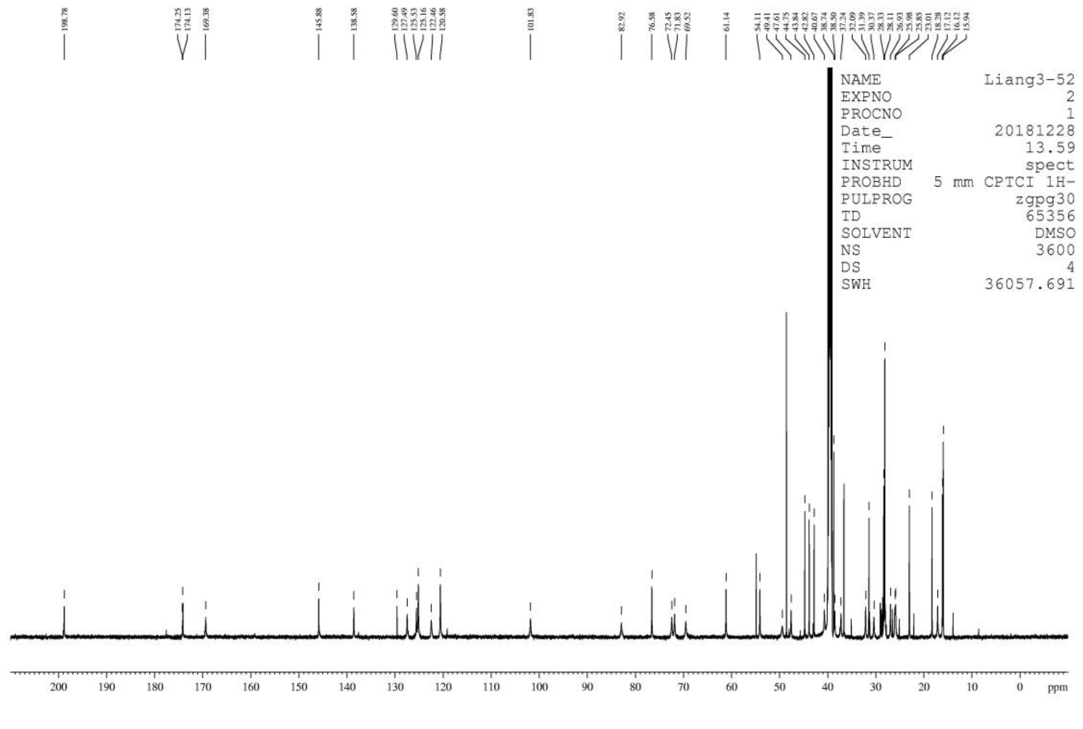

HSQC of compound **36**

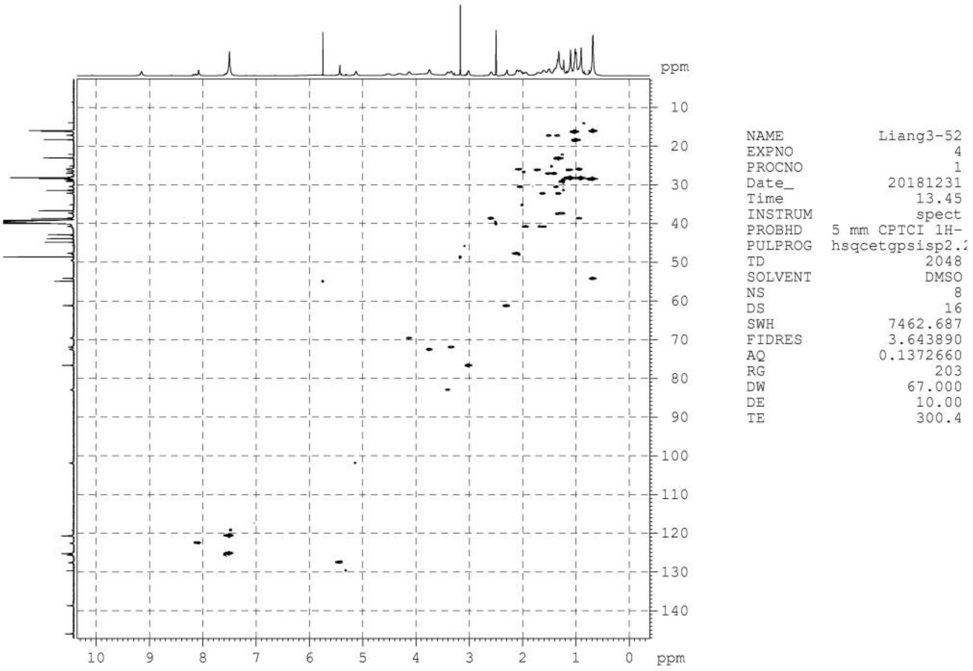

MALDI-TOF of compound **36**

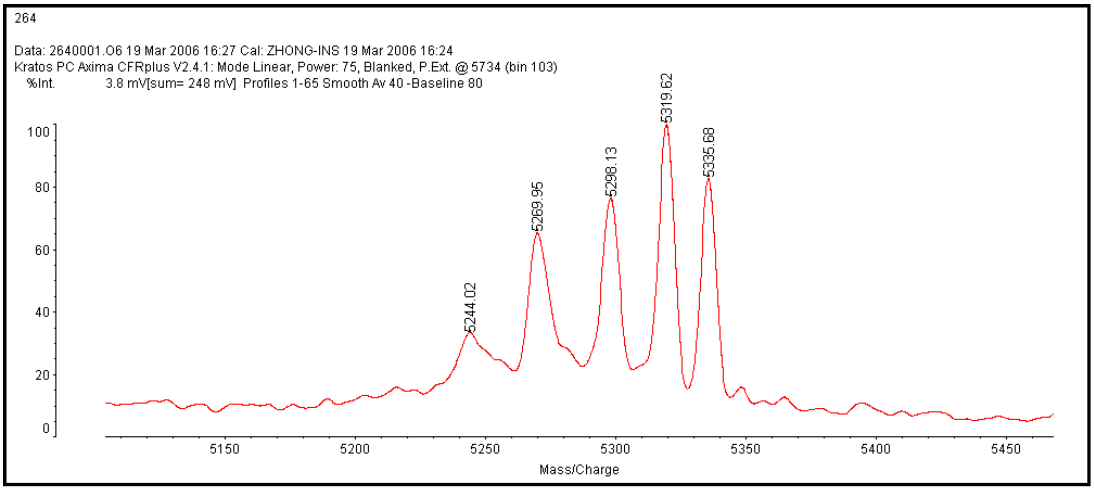

^{1}H NMR of compound **37**

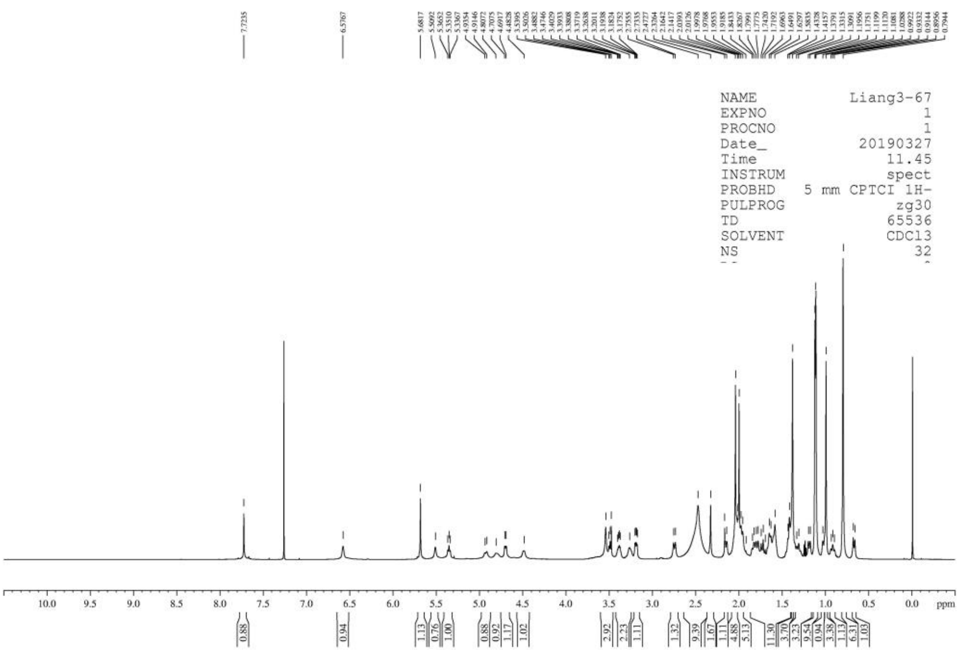

^{13}C NMR of compound **37**

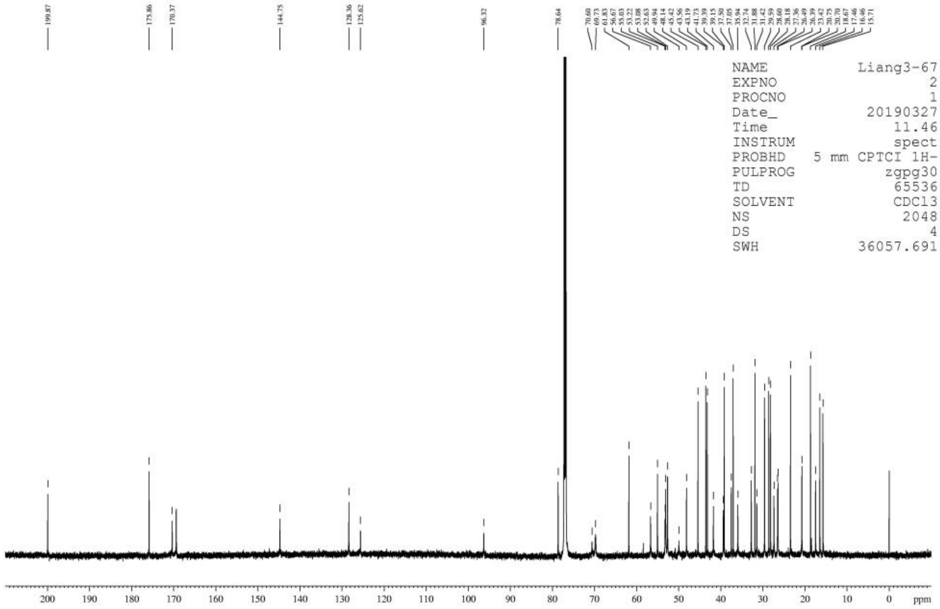

HSQC of compound **37**

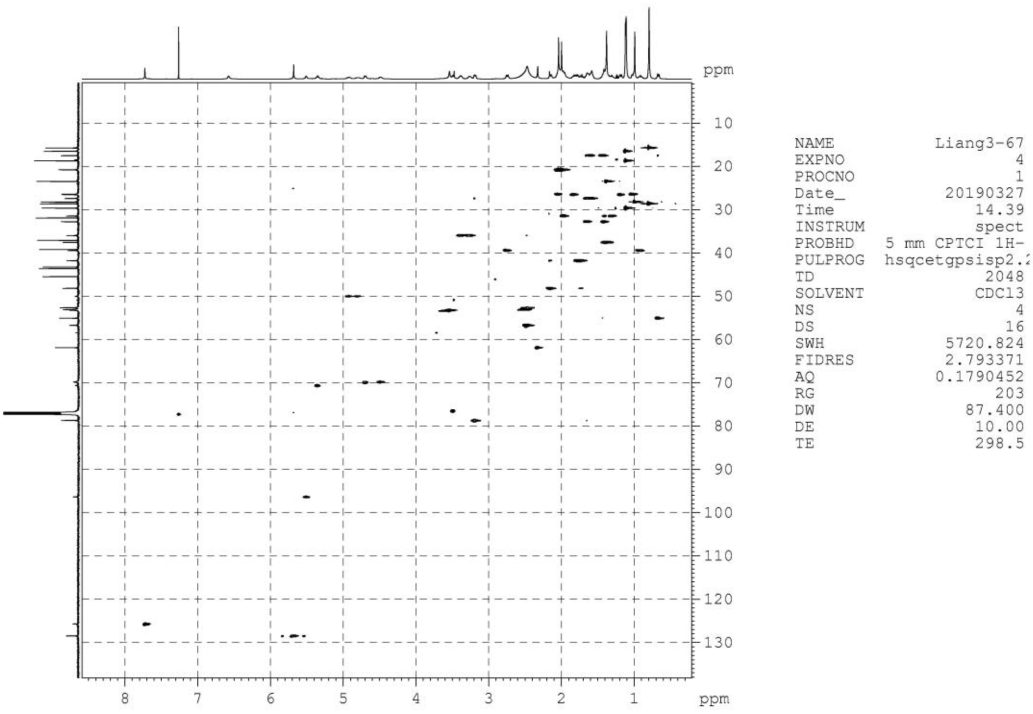

MALDI-TOF of compound **37**

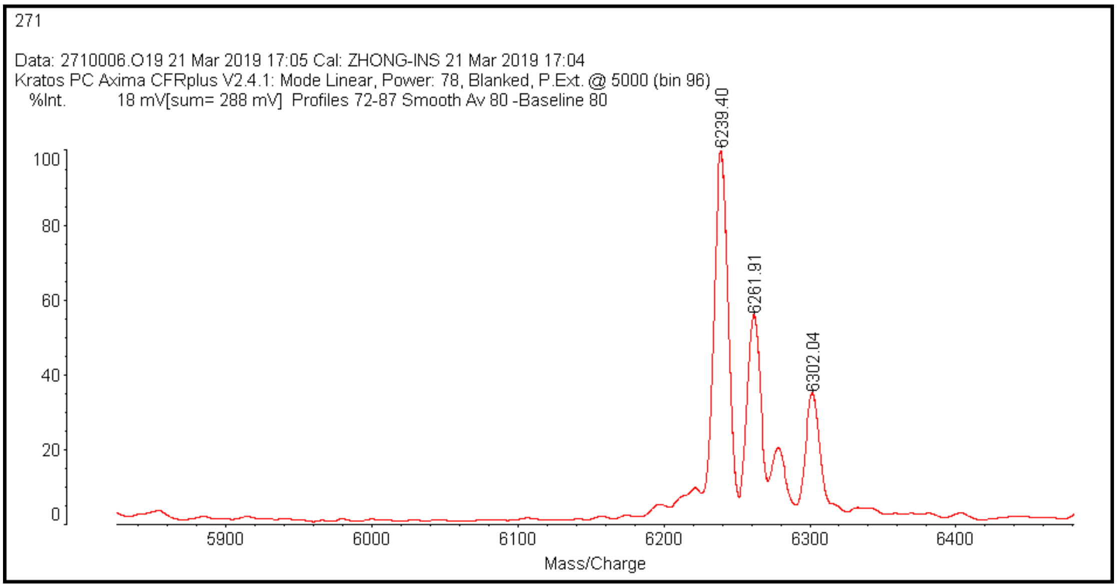

^{1}H NMR of compound **38**

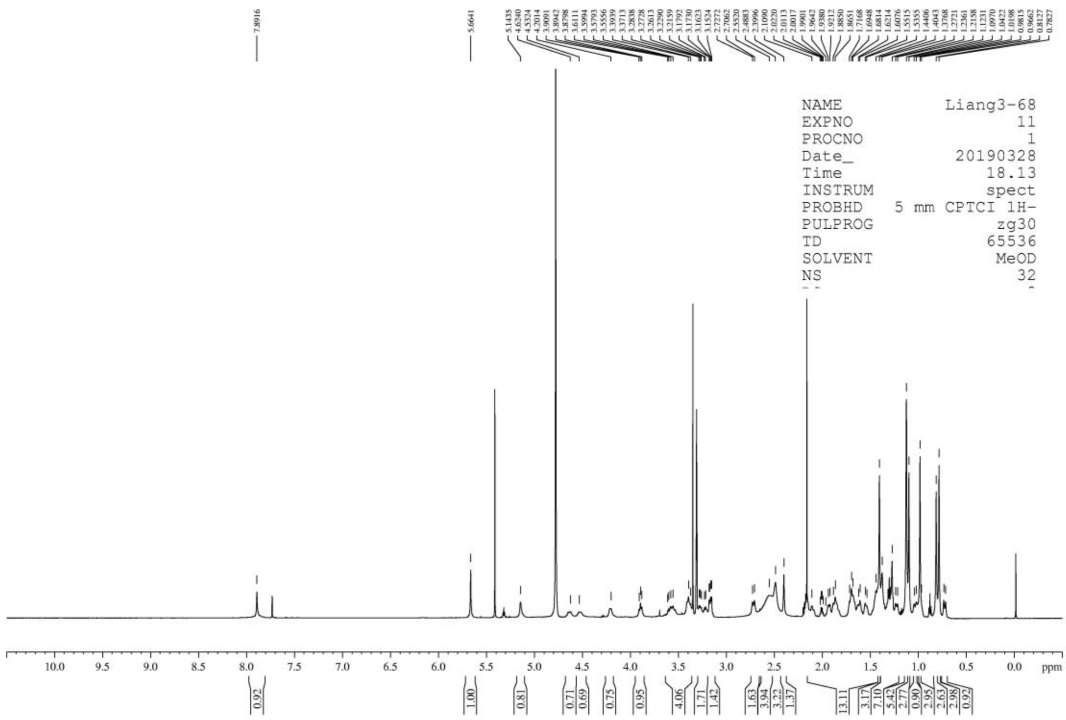

^{13}C NMR of compound **38**

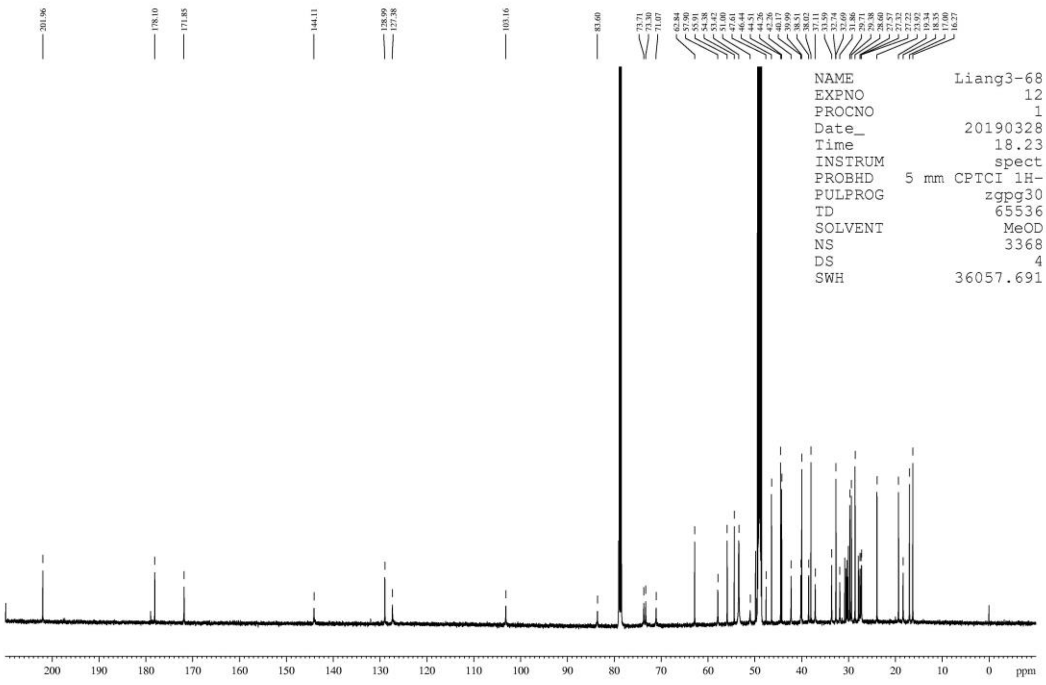

HSQC of compound **38**

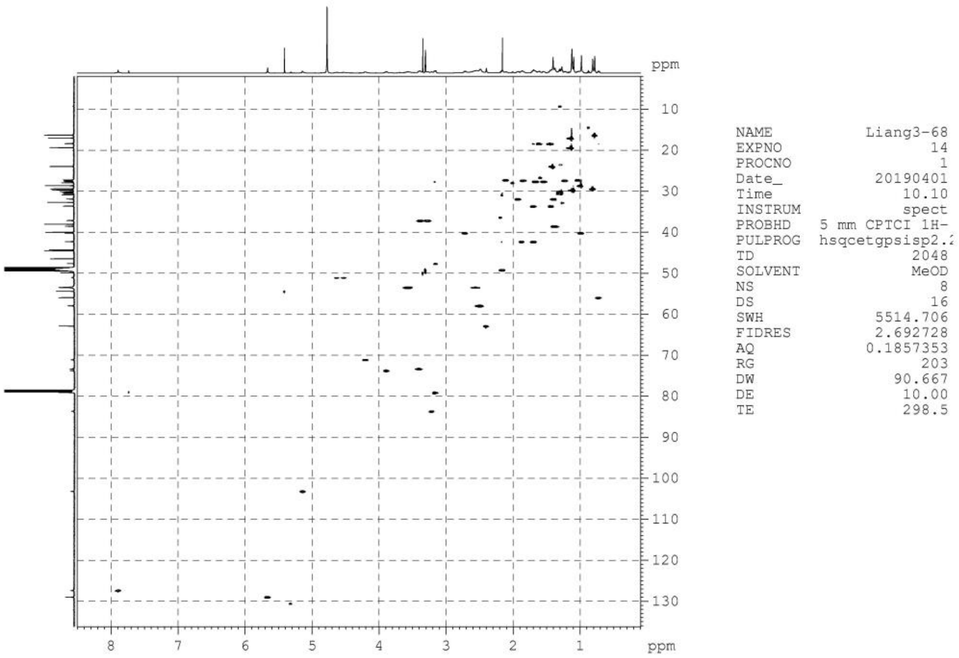

MALDI-TOF of compound **38**

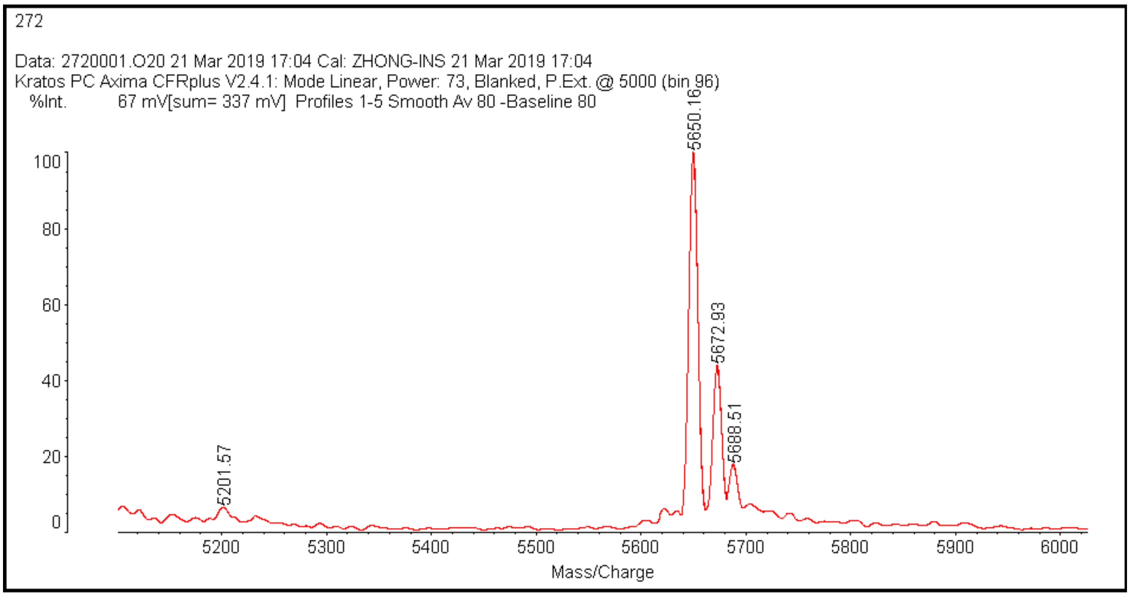

Supplement: Supplementary file 1 [file DataSheet1.pdf]
